# Repeated gain and loss of a single gene modulates the evolution of vascular plant pathogen lifestyles

**DOI:** 10.1126/sciadv.abc4516

**Published:** 2020-11-13

**Authors:** Emile Gluck-Thaler, Aude Cerutti, Alvaro L. Perez-Quintero, Jules Butchacas, Verónica Roman-Reyna, Vishnu Narayanan Madhavan, Deepak Shantharaj, Marcus V. Merfa, Céline Pesce, Alain Jauneau, Taca Vancheva, Jillian M. Lang, Caitilyn Allen, Valerie Verdier, Lionel Gagnevin, Boris Szurek, Gregg T. Beckham, Leonardo De La Fuente, Hitendra Kumar Patel, Ramesh V. Sonti, Claude Bragard, Jan E. Leach, Laurent D. Noël, Jason C. Slot, Ralf Koebnik, Jonathan M. Jacobs

**Affiliations:** 1 Department of Plant Pathology, The Ohio State University, Columbus, OH 43210, USA.; 2 Department of Biology, University of Pennsylvania, Philadelphia, PA 19104, USA.; 3 LIPM, Université de Toulouse, INRAE, CNRS, Université Paul Sabatier, Castanet-Tolosan, France.; 4 Agricultural Biology, Colorado State University, Fort Collins, CO, USA.; 5 Infectious Disease Institute, The Ohio State University, Columbus, OH 43210, USA.; 6 CSIR-Centre for Cellular and Molecular Biology, Hyderabad 500007, India; 7 Department of Entomology and Plant Pathology, Auburn University, Auburn, AL 36849, USA.; 8 IRD, CIRAD, Université Montpellier, IPME, Montpellier, France.; 9 Earth & Life Institute, Université Catholique de Louvain, Louvain-la-Neuve, Belgium.; 10 HM Clause (Limagrain group), Davis, CA, 95618, USA.; 11 Institut Fédératif de Recherche 3450, Plateforme Imagerie, Pôle de Biotechnologie Végétale, Castanet-Tolosan, France.; 12 Department of Plant Pathology, University of Wisconsin–Madison, Madison, WI 53706, USA.; 13 Renewable Resources and Enabling Sciences Center, National Renewable Energy Laboratory, Golden, CO 80401, USA.

## Abstract

Vascular plant pathogens travel long distances through host veins, leading to life-threatening, systemic infections. In contrast, nonvascular pathogens remain restricted to infection sites, triggering localized symptom development. The contrasting features of vascular and nonvascular diseases suggest distinct etiologies, but the basis for each remains unclear. Here, we show that the hydrolase CbsA acts as a phenotypic switch between vascular and nonvascular plant pathogenesis. *cbsA* was enriched in genomes of vascular phytopathogenic bacteria in the family Xanthomonadaceae and absent in most nonvascular species. CbsA expression allowed nonvascular *Xanthomonas* to cause vascular blight, while *cbsA* mutagenesis resulted in reduction of vascular or enhanced nonvascular symptom development. Phylogenetic hypothesis testing further revealed that *cbsA* was lost in multiple nonvascular lineages and more recently gained by some vascular subgroups, suggesting that vascular pathogenesis is ancestral. Our results overall demonstrate how the gain and loss of single loci can facilitate the evolution of complex ecological traits.

## Teaser

A single gene with a dynamic evolutionary history allows plant pathogenic bacteria to spread systemically through their hosts.

## INTRODUCTION

Pathogenic microorganisms cause diseases of animals and plants. Some pathogenic species colonize the host vasculature, which leads to systemic infection, while others remain localized to nonvascular tissues. Complex structural and biochemical differences between vascular and nonvascular tissues suggest that pathogens have multiple distinct adaptations to either environment, yet the genetic and evolutionary bases of these adaptations are largely unknown.

Adaptations often occur through wholesale gain and loss of specific genes, resulting in more rapid evolution compared with incremental changes at the DNA sequence level alone (*1*). In bacteria, gene gain occurs primarily through horizontal gene transfer (HGT), while gene loss or pseudogenization occurs through multiple mechanisms, including transposon-mediated insertions and sequence deletions in open reading frames (*2*–*4*). Especially for loci encoding ecologically relevant traits, gene gain and loss effectively act as phenotypic switches, enabling rapid shifts between what otherwise seem like complex lifestyles (*3*). For example, transitions between plant pathogenic and commensal *Pseudomonas* (*5*), transitions between mutualist and parasitic phenotypes in nitrogen-fixing bacteria (*6*, *7*), and transitions between mutualistic and plant pathogenic *Rhodococcus* (*8*) have all been shown to reproducibly occur through the gain and loss of genomic islands containing multiple genes all contributing to the same phenotype. These rapid evolutionary dynamics have profound implications for our understanding of disease ecology and disease management strategies.

In plants, vascular xylem and nonvascular parenchyma tissues represent distinct niches. Xylem is composed of dead cells with highly reinforced walls organized into cylinders that provide plants with structural integrity and a means of long-distance fluid transport. In contrast, parenchyma tissues are composed of living cells and gas-filled intercellular spaces. Xylem fluid consists primarily of water and mineral nutrients, and many vascular pathogens successfully colonize this plant environment (*9*). Xylem tissue not only runs throughout the plant, enabling the distribution of water from roots to leaves, but also serves as a potential pathway for rapid, systemic transport of pathogens.

The gammaproteobacterial family Xanthomonadaceae includes two major genera, *Xanthomonas* and *Xylella*, that cause vascular diseases of plants. Bacteria in the genus *Xylella* are fastidious, insect-vectored vascular pathogens. *Xanthomonas* is a diverse genus of plant-associated Gram-negative bacterial species that cause vascular and nonvascular diseases of more than 350 monocot and dicot plant hosts (*10*). *Xanthomonas* species are separated into subgroups called pathovars (pv.) based on their phenotypic behavior such as symptom development (e.g., vascular or nonvascular) or host range (*10*). Vascular xanthomonads invade the water-transporting xylem; nonvascular *Xanthomonas* species cause localized symptoms by colonizing the mesophyll. Although often closely related, the genetic determinants distinguishing vascular from nonvascular *Xanthomonas* lineages at the intraspecific level are not clear.

Here, we used *Xanthomonas* as a model to study the etiology of plant vascular pathogenesis because this genus contains multiple independent pairs of strains from the same species (i.e., pathovars) that cause either vascular or nonvascular diseases. This enabled us to disentangle genetic features that are shared due to ancestry and those that may be shared due to common tissue-specific lifestyles. Given the tendency of bacteria to evolve through the gain and loss of genes organized into clusters or genomic islands, we hypothesized that vascular and nonvascular pathogenesis emerge through the gain and loss of small numbers of linked loci. We found evidence supporting the most extreme version of this hypothesis, where transitions between vascular and nonvascular lifestyles are mediated by the repeated gain and loss of a single gene that acts as a phenotypic switch.

## RESULTS

### *cbsA* is significantly associated with vascular pathogenesis

**Fig. 1:**
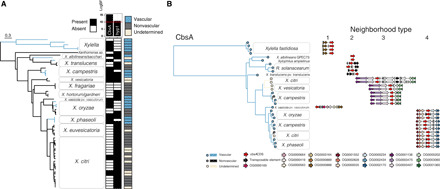
The cellobiohydrolase CbsA is associated with transitions to vascular pathogenic lifestyles in Gram-negative pathogens. (**A**) Highest-ranking associations between OG presence/absences and evolutionary transitions between vascular and nonvascular lifestyles in the Xanthomonadaceae. A genome-based SNP phylogeny is shown to the left, with strains from the same species condensed into clades. A heatmap summarizing, for each strain, the presence (black) or absence (white) of the two gene OGs, CbsA and hyp1, whose distributions are most strongly supported to be dependent on vascular lifestyle status (determined by model testing through the ranking of log Bayes factors; Materials and Methods) is shown to the right of the tree, followed by another heatmap indicating the classification of each strain as either vascular (blue), nonvascular (gray), or undetermined (beige) according to the literature (table S1). Additional figure details can be found in figs. S1 and S5. (**B**) Phylogenetic tree based on CbsA amino acid sequences from strains with whole-genome sequences found in (A), where branches on the tree are color-coded according to pathogenic lifestyle. To the right of each tip is a schematic depicting the neighborhood type in which that particular *cbsA* sequence is found, where the four possible neighborhood types are defined based on conserved synteny (indicated by color-coded gene models corresponding to specific OGs). Vascular bacteria have *cbsA* homologs located in type 1, 2, and 4 neighborhoods, while nonvascular bacteria have *cbsA* homologs found primarily in type 3 neighborhoods. Note that strains of the vascular pathogen *X. campestris* pv. campestris have two copies of *cbsA* located in either type 3 or 4 neighborhoods.

We first identified high-priority candidate genes associated with transitions to vascular and nonvascular lifestyles. We classified annotated proteins from 59 publicly available whole-genome sequences of plant pathogenic Xanthomonadaceae bacteria in the *Xanthomonas* and *Xylella* genera into ortholog groups (OGs). We then conducted an analysis of trait evolution across a single-nucleotide polymorphism (SNP)–based phylogeny where, for each OG, we tested the hypothesis that transitions to vascular or nonvascular lifestyles were dependent on that OG’s presence or absence using BayesTraitsV3 (Fig. 1) (*11*). The phylogenetic relationships between vascular and nonvascular pathovars indicated that xylem pathogenesis is paraphyletic, i.e., not limited to a single clade, an individual Xanthomonas sp., or host plant genus (Fig. 1 and figs. S1 and S2). Instead, vascular diseases of many host plant families are caused by different pathovars across the *Xanthomonas* genus. We identified two OGs whose presence was strongly associated with the distribution of tissue-specific lifestyles (Fig. 1, fig. S1, and tables S1 and S2). One OG (OG0003492, log Bayes factor = 15.19) was highly associated with vascular pathogenesis, while the other (OG0002818, log Bayes factor = 10.51) was associated with nonvascular pathogenesis. For this study, we focused on vascular pathogen-enriched OG0003492, which encodes a cell wall–degrading cellobiohydrolase (EC 3.2.1.4, glycosylhydrolase family GH6) called CbsA (*12*, *13*).

CbsA was present in all taxa classified as vascular with one exception (*Xanthomonas hortorum*) and was absent from most nonvascular taxa. CbsA was also found in some strains with undetermined tissue specificity due to unavailable or conflicting information in the literature (table S1). Phylogenetic analysis of CbsA sequences revealed that within *Xanthomonas*, CbsA sequences form two major clades: the first contains sequences found in vascular, nonvascular (or undetermined) pathogen genomes (found in type 3 neighborhood in Fig. 1B; see below), and the second contains sequences found exclusively in vascular pathogen genomes (found in type 4 neighborhoods in Fig. 1B; see below). All vascular pathogens with a CbsA homolog found in the first clade also have a CbsA homolog found in the second clade, effectively having two copies of the CbsA gene (Fig. 1B and fig. S3). The observation that CbsA sequences from the second clade are found only in vascular pathogen genomes, while sequences from the first clade are found in both vascular and nonvascular pathogen genomes, suggests that sequences from different clades have distinct biological functions.

### Heterologous expression of *cbsA* bestows vascular pathogenesis to a nonvascular pathogen

Because *cbsA* was largely present in vascular and often absent from nonvascular *Xanthomonas* species, we hypothesized that *cbsA* was either (i) gained by vascular *Xanthomonas* species or (ii) lost by nonvascular *Xanthomonas* species. To experimentally test the alternate models, we examined the effects of manipulating *cbsA* on the contrasting tissue-specific behavior of two closely related barley pathogens from the same species: vascular *Xanthomonas translucens* pvs. translucens (Xtt) and nonvascular undulosa (Xtu).

**Fig. 2:**
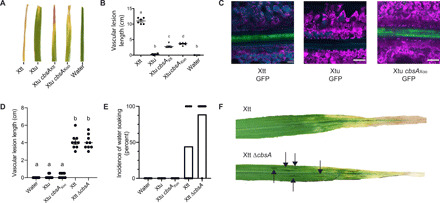
Experimental gain and loss of CbsA facilitates transitions between vascular and nonvascular pathogenic lifestyles. (**A**) Addition of either *cbsA* from vascular *X. translucens* pv. translucens (Xtt) or *cbsA* from vascular *X. oryzae* pv. oryzae (Xoo) to nonvascular *X. translucens* pv. undulosa (Xtu) permits development of chlorotic lesions indicative of vascular disease on barley 21 days post-inoculation (dpi). (**B**) Corresponding vascular lesion lengths, with significant differences among treatments indicated by a to d (*n* = 6, *P* < 0.02). (**C**) Representative confocal images of vascular bundles downstream of leaf lesions on barley 12 dpi with GFP transformed strains demonstrate gain of vascular colonization by Xtu *cbsA*_Xoo_. Green indicates bacterial cells expressing GFP; magenta indicates chlorophyll autofluorescence outlining nonvascular mesophyll cells; cyan indicates autofluorescence outlining xylem cell walls or phenylpropanoid accumulation in mesophyll cells. (**D** and **E**) Lesion lengths or incidence of nonvascular water-soaked lesions were quantified after barley leaf clipping 14 dpi with Xtt ∆*cbsA*. Bars in (E) represent percent leaves showing symptoms with dots included to display individual leaf lesion incidence. (**F**) Images of symptomatic barley leaves infected with Xtt and Xtt ∆*cbsA*, where water-soaked lesions are indicated, with black arrows indicating nonvascular symptom development.

Xtt and Xtu both cause nonvascular bacterial leaf streak (BLS) disease of barley (*14*). However, only Xtt can colonize the xylem, which is required for long-distance bacterial blight (BB) symptom development (Fig. 2, A to C) (*14*, *15*). Upon leaf clipping, only Xtt produces distant vascular BB; meanwhile, Xtu symptoms remain near the site of inoculation (Fig. 2A). Moreover, Xtt strains contain an intact copy of *cbsA*, while *X. translucens* pv. undulosa contains a copy of *cbsA* that is disrupted in the 5′ region by a transposase (fig. S4).

As Xtt has *cbsA*, while Xtu lacks an intact copy, we tested whether the expression of CbsA promotes vascular symptom development in Xtu. Xtu miniTn*7*::*cbsA*_Xtt_, a single insertion variant with an intact copy of *cbsA* from Xtt, caused distant leaf lesions of approximately 4.5 cm (Fig. 2, A and B). Moreover, expression of the characterized CbsA ortholog from the vascular rice pathogen Xoo (Xtu miniTn*7*::*cbsA*_Xoo_) also permitted Xtu to cause distant symptom development consistent with a vascular pathogenic lifestyle. Using green fluorescent protein (GFP)–expressing strains, we reproducibly observed Xtu miniTn7::*cbsA*_Xoo_ inside the xylem similar to Xtt (Fig. 2C). Wild-type (WT) Xtu did not produce vascular symptoms and was not detected in distant xylem vessels (Fig. 2C). Therefore, the gain of *cbsA* from either of two different vascular pathogens is sufficient to promote xylem-mediated colonization and distant infection of leaves by nonvascular Xtu.

### Impact of *cbsA* mutagenesis on vascular pathogenesis is dependent on genetic background

We found that the Xtt ∆*cbsA* mutant was still capable of causing vascular leaf blight, suggesting that other unknown factors support vascular pathogenesis beyond CbsA alone (Fig. 2, D and F). However, while Xtt ∆*cbsA* could still cause systemic symptom development, the mutation of this cellulase altered this strain’s pathogenic behavior by promoting the development of nonvascular, water-soaked lesions adjacent to blight symptoms on 90% of infected leaves compared with only 40% of leaves on plants infected with WT vascular Xtt (Fig. 2, E and F). These water-soaked symptoms are typical of nonvascular disease development in Xtt and Xtu (*14*, *15*). Therefore, while vascular disease development is not completely abolished by *cbsA* mutagenesis, the absence of *cbsA* increased the development of nonvascular disease symptoms.

These results did not match previous reports that *cbsA* deletion mutants in *Xanthomonas oryzae* pv. oryzae and *Ralstonia solanacearum* have reduced systemic virulence and vascular pathogenesis (*16*, *17*). We therefore replicated and expanded upon these previous findings by mutating *cbsA* in *X. oryzae* pv. oryzae and *Xylella fastidiosa* (Xanthomonadaceae). *X. oryzae* pv. oryzae causes BB of rice with systemic symptoms similar to Xtt on barley. *X. fastidiosa*, an insect-vectored, xylem pathogen, is the causal agent of Pierce’s disease of grape and the emerging olive quick decline disease. *X. oryzae* pv. oryzae and *X. fastidiosa* deletion mutants were severely reduced in vascular symptom development, confirming and building upon previous reports (fig. S5) (*16*). The variable effects of mutagenizing *cbsA* in Xtt versus *X. oryzae* pv. oryzae and *X. fastidiosa* indicate that the robustness of vascular phenotypes is lineage dependent within *Xanthomonas*, with certain species likely having multiple determinants in addition to *cbsA* that contribute to vascular pathogenesis.

### The genomic location of *cbsA* alternates between four distinct neighborhoods

**Fig. 3:**
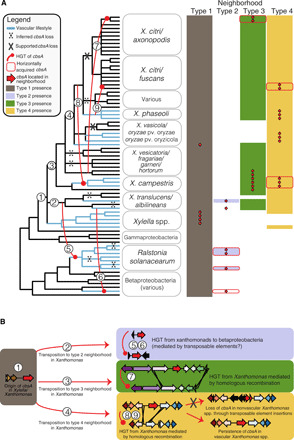
Repeated horizontal transfer, transposition, and gene loss events drive the distribution of *cbsA* in Gram-negative bacteria. (**A**) A 50% majority-rule consensus tree summarizing 81 conserved single-copy ortholog trees is shown to the left, with the names of the 75 individual isolates consolidated into relevant taxonomic groupings. Inferred HGT, transposition, and loss events are drawn and numbered on the tree and further described in (B). The matrix to the right of tree indicates the presence/absence of one of four distinct genomic neighborhood types (shaded/unshaded cells) in which *cbsA* homologs are found within a given genome (presence of *cbsA* indicated by an overlaid red arrow). Note that in many cases, all of the constituent genes making up a specific neighborhood are present in a given genome save for *cbsA* (indicated by the absence of an overlaid red arrow). *cbsA* homologs from *X. albilineans* and *X. ampelinus* were not found associated with a specific type of neighborhood, but were assigned to the type 2 neighborhood column based on the observation that their closest phylogenetic relatives are sequences in type 2 neighborhoods (see Fig. 1B). This tree has been lightly edited for viewing purposes by removing several taxa from outside the Xanthomonadales and can be viewed in its entirety in fig. S3. (**B**) Sequence of inferred evolutionary events numbered corresponding to (A). Genomic neighborhood types are represented by schematics, where gene models are color-coded according to OG. The color-coding of neighborhood types is consistent across both panels.

Across all examined genomes, *cbsA* is found embedded in one of four genomic neighborhood types with conserved gene synteny (Figs. 1B and 3). The localization of *X. fastidiosa*’s and *X. vasicola*’s *cbsA* in type 1 neighborhoods, combined with a lack of evidence suggesting HGT between these two species (Fig. 1B), provides support that *cbsA* was present and organized in a type 1 context in the last common ancestor of *Xanthomonas* and *Xylella*. Based on this inference, it is likely that *cbsA* was then re-located into type 2, 3, and 4 neighborhoods through separate cis-transposition events as *Xanthomonas* spp. diversified. The timing of transposition events 3 and 4 is uncertain due to the lack of resolution in species-level relationships, but likely occurs near to where indicated on the species tree (Fig. 3). Within the gammaproteobacteria, all known vascular pathogens in our dataset have a copy of *cbsA* localized in the context of type 1, 2, or 4 neighborhoods. Within *Xanthomonas*, sequences from the clade of CbsA homologs found in both vascular and nonvascular pathogens are located in type 3 neighborhoods, while sequences from the clade of CbsA homologs found exclusively in vascular pathogen genomes are located in type 4 neighborhoods, further supporting the hypothesis that sequences belonging to either of these two clades have separate functions (Fig. 2).

### *cbsA* has been independently gained by lineages now displaying vascular lifestyles

*cbsA* and varying lengths of adjacent sequence experienced three horizontal transfers in the *Xanthomonas* genus mediated by homologous recombination events in flanking gene neighborhoods (events 7 to 9 in Fig. 3; figs. S6 to S8). Two transfers from what was likely the ancestor of the vascular pathogen *X. phaseoli* are coincident with the emergence of vascular lifestyles in xylem-adapted *X. campestris* pv. campestris and potentially xylem-colonizing *X. citri* pv. phaseoli and occurred within the context of type 4 neighborhoods (events 8 and 9, Fig. 3; figs. S6 to S8). The third transfer occurred in the context of a type 3 neighborhood, where neither the donor lineage of *X. vesicatoria* nor the recipient lineage of *X. citri* has been reported to be capable of vascular pathogenesis.

### *cbsA* was horizontally transferred from vascular gamma- to betaproteobacteria

We found additional evidence that *cbsA* was horizontally transferred from gammaproteobacterial Xanthomonadaceae to the betaproteobacterial xylem plant pathogens *R. solanacearum* and *Xylophilus ampelinus* (Fig. 3). *cbsA* sequences in both *X. translucens* pv. translucens and *R. solanacearum* are flanked on one or both sides by transposable elements (Fig. 1B), providing a plausible mechanism for mediating horizontal transfer through transposition between these distant lineages. However, we could not test this specific hypothesis with confidence because the phylogenies of the transposable elements in question are complex and contain signatures of extensive horizontal transfer between strains.

### *cbsA* has been repeatedly lost from lineages now displaying nonvascular lifestyles

At least 10 losses of *cbsA* are required to parsimoniously explain its distribution across the beta- and gammaproteobacteria when taking into account all HGT events supported by phylogenetic hypothesis testing (Fig. 3 and tables S4 to S6). While most of the losses are inferred using parsimony criteria (e.g., losses in nonvascular strains of *X. hortorum* and *X. fragariae*; Materials and Methods), several *cbsA* pseudogenes present in extant species directly support the hypothesis of repeated, independent losses through distinct inactivation mechanisms. For example, *cbsA* was independently pseudogenized in the nonvascular *X. translucens* pv. undulosa and *X. sacchari* through sequence deletions in its 5′ coding region (figs. S4 and S6). In contrast, transposable elements have disrupted the 5′ region of *cbsA* in nonvascular *X. oryzae* pv. oryzicola and are present in the type 4 neighborhoods of certain nonvascular *X. citri* subsp. citri and *X. fuscans* subsp. aurantifolii isolates that lack a copy of *cbsA* (fig. S6). These examples of multiple, independent disruptions to *cbsA* in lineages displaying nonvascular lifestyles suggest that nonvascular pathogenesis convergently evolved through repeated gene loss.

## DISCUSSION

**Fig. 4:**
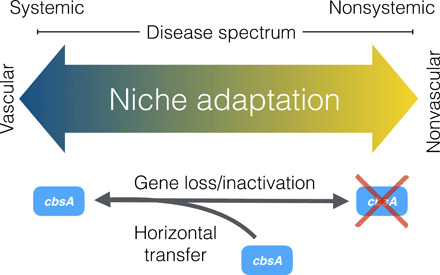
The evolution of vascular and nonvascular pathogenesis in plant-associated *Xanthomonas* bacteria is driven by the gain and loss of *cbsA*. Our combined phenotypic and phylogenetic analyses support a model where vascular and nonvascular pathogenesis exist as two points on the same evolutionary continuum that is traversed by either the acquisition or loss of a single cellobiohydrolase, *cbsA*.

Systemic pathogens traverse host veins to move long distances, leading to life-threatening systemic infections. In contrast, nonvascular pathogens remain restricted to the site of infection, triggering localized symptom development with far fewer implications for host health. Although complex differences between these modes of infection suggest that they have radically different origins, the results we present here suggest that vascular and nonvascular pathogenesis are two points on an evolutionary continuum, a finding with important implications for understanding and predicting pathogen evolution (Fig. 4). By integrating comparative genomic, phylogenetic, and functional genetic analyses, we found evidence that vascular and nonvascular plant pathogenic lifestyles emerge from the repeated gain and loss of a single gene that can act as a phenotypic switch.

Our functional and phylogenetic results suggest that *cbsA* contributes to the evolution of *Xanthomonas* vascular pathogenicity, but to varying extent depending on the species considered. The function of CbsA remains enigmatic, but CbsA could hypothetically promote movement via degradation of pit membranes and/or a nutrient source by the release of cellobiose from cellulose. Xylem-specific pathogens, including *X. fastidiosa*, *X. oryzae* pv. oryzae, and *R. solanacearum,* require CbsA for vascular pathogenesis, whereas Xtt, which induces both vascular and nonvascular disease symptoms, appears to use other factors beyond CbsA to colonize xylem vasculature. That the phenotypic outcomes of CbsA acquisition are dependent on genetic background suggests that there exist multiple evolutionary routes to vascular pathogenesis and highlights the particularities of specific host-pathogen interactions. Nevertheless, the preponderance of phenotypic and phylogenetic evidence supports the hypothesis that *cbsA* was present in the last common ancestor of *Xanthomonas* and *Xylella* and has since played not only a historical but also possibly a contemporary role in driving the emergence and reemergence of tissue-specific behavior in the Xanthomonadaceae.

While we document repeated gains and losses of *cbsA,* the conditions that favor phenotypes resulting from either its presence or absence remain to be determined. Although *cbsA* homologs are among the highest expressed genes during xylem pathogenesis (*9*, *18*), and are required for vascular pathogenesis in several species (fig. S5), the contributions of CbsA to pathogen fitness remain unclear. Current theory suggests that there may be a fitness cost to retaining this gene and the vascular lifestyle it enables, given that CbsA induces immune responses and can prime the plant against *Xanthomonas* infection (*16*). Furthermore, cell wall degradation products, such as the CbsA enzymatic biproduct cellobiose, could act as a danger-associated molecular pattern in the plant mesophyll and may induce plant defenses through WRKY transcription factors (*19*). We therefore speculate that *cbsA*’s absence may be selected for to dampen recognition by the host and/or the elicitation of host immunity; however, these hypotheses remain to be tested.

Gene loss is a fundamental mechanism of adaptation (*20*). Especially for loci with large effects such as *cbsA*, only a minimal number of loss events are required to incur appreciable changes to phenotype. Adaptive phenotypes arising through loss of function may emerge over shorter time scales compared with adaptive phenotypes arising through gains in function, as genes typically have more mutational opportunities for losing functions than for gaining functions (*21*). Even within our own limited dataset, we observed multiple mutational routes in the form of sequence deletions and transposable element insertions that led to the convergent loss of *cbsA* in different nonvascular pathogen lineages, which suggests that nonvascular phenotypes readily emerge in the Xanthomonadaceae.

Although there may be fewer mutational routes for gaining gene functions compared with losing them, our phylogenetic analyses revealed that rates of gain and loss may be influenced by latent patterns in genome architecture, such as the conservation of synteny. Homologous recombination in bacteria is typically studied within species and is considered to be important for maintaining genetic diversity in what would otherwise be clonal lineages (*22*). Less considered are the impacts of homologous recombination across species. Our results add to a growing body of literature suggesting that, while perhaps less common than intraspecific homologous recombination (*23*, *24*), interspecific gene exchange facilitated by homologous recombination at syntenic loci is an important mechanism of adaptation (*25*). All three *cbsA* HGT events within *Xanthomonas* occurred through homologous recombination in syntenic neighborhoods flanking *cbsA* presence/absence polymorphisms, and two of these resulted in the reversal of an ancestral loss event (Fig. 2), suggesting that synteny conservation potentiates not only gene gain but also the reversal of lineage-specific gene loss. By effectively increasing an individual strain’s ability to access cross-species pan-genomic material, the conservation of synteny is likely to be an important accelerator of ecological adaptation.

Overall, our study provides an integrated evolutionary and functional framework for studying the genetic bases of transitions between vascular and nonvascular pathogen lifestyles (Fig. 4). Our experiments demonstrate that the acquisition of *cbsA* is sufficient for long-distance systemic pathogenesis in specific *Xanthomonas* pathogens. Conversely, the loss of *cbsA*, while not necessary to abolish vascular disease development, is sufficient for the development of nonvascular disease symptoms. We add to a growing body of literature that suggests that transitions between distinct bacterial ecotypes may be mediated by the recurrent gain and loss of few loci (*5*, *8*). Although it remains to be determined how the processes of rapid gene gain and loss affect vascular and nonvascular evolution in other pathogenic microbes, our work suggests that these evolutionary events play an important role in shaping bacterial adaptation to specific host tissues.

## MATERIALS AND METHODS

### Comparative genomics for identification of vascular pathogen-specific genes

Using Orthofinder v2.2.3 (*26*), we first created OGs from all annotated amino acid sequences derived from 171 complete and 8 partially complete publicly available assemblies from the Xanthomonadaceae and representative lineages across the beta- and gammaproteobacteria to obtain a comprehensive comparative genomic dataset (table S1). Consensus functional annotations for each OG were obtained by determining the most frequent protein family domain present among the members of the OG using InterProScan version 5.25-64.0 (*27*). Predicted proteins across all genomes were classified into 36,905 OGs using Orthofinder (table S2) (*26*).

Genomes were classified as vascular, nonvascular, or unknown on the basis of available information in the literature (table S1). The *Xanthomonas* species included xylem and parenchyma pathogens that infect diverse dicot and monocot crops such as rice, wheat, barley, cabbage, tomato, citrus, and common bean. A distant vascular grape and citrus Xanthomonadaceae bacterium, *X. fastidiosa*, was also analyzed.

For analyses limited to the Xanthomonadaceae, we built a more resolved SNP-based parsimony tree using kSNP3 (*28*) from a set of publicly available complete and annotated genomes from different species in the Xanthomonadaceae family (optimum kmer size = 21; table S1). Using the kSNP3 as a reference, associations were identified between the presence/absence of each OG in the analyzed genomes and the vascular/nonvascular trait using BayesTraits V3 (*11*). The likelihood that both traits (vascularity versus gene presence) evolved independently was compared to the likelihood they evolved dependently. Evidence of dependent evolution was assessed as log Bayes factors = 2(log marginal likelihood dependent model – log marginal likelihood independent model), and genes with a log Bayes factor > 10 were considered to have strong evidence of dependent evolution.

### Bacterial strains and growth conditions

The bacterial strains used in this study are listed in table S7. *Escherichia coli* strains were grown at 37°C in lysogenic broth (LB) medium. *E. coli* bearing the pUC4K plasmid was grown on LB medium at 37°C. When needed, the antibiotic kanamycin (Km) was used at the concentration of 50 μg/ml. *X. translucens* or *X. oryzae* cells were grown at 28°C on solid nutrient agar, liquid nutrient broth, or peptone-sucrose–rich media (*15*). When necessary, media were supplemented with gentamicin (15 μg/ml), Km (25 μg/ml), or spectinomycin (50 μg/ml). See table S7 for specific strains used in this study. *X. fastidiosa* subsp. *fastidiosa* TemeculaL WT (*24*) and *X. fastidiosa* subsp. *fastidiosa* str. TemeculaL Δ*cbsA* mutant were used in this study (table S7). Strains were cultured on PW (periwinkle wilt) agar media (*29*), modified by removing phenol red and using bovine serum albumin (1.8 g/liter) (Gibco Life Sciences Technology), for 7 days at 28°C from −80°C glycerol stocks, and subcultured onto fresh PW agar medium plates for another 7 days at 28°C before use. All assays were performed using the subcultured *X. fastidiosa* strains. PD3 broth media and phosphate-buffered saline (PBS) buffer were used for suspending cells in liquid.

### Recombinant DNA techniques

Total genomic and plasmid DNA were isolated by standard methods. *E. coli*, *Xanthomonas* species, and *X. fastidiosa* were transformed as previously described (*15*, *16*, *29*). To construct complementation vectors of *cbsA*_Xtt_ and *cbsA*_Xoo_, the gene regions including the native promoters were polymerase chain reaction (PCR)–amplified from *X. translucens* pv. translucens str. UPB886. Each was cloned into pUC18miniTn7T to create pUC18miniTn7T::*cbsA*_Xtt_ and pUC18miniTn7T::*cbsA4*_Xoo_ (*30*). For gene expression, *X. translucens* pv. undulosa strains were transformed with miniTn*7* plasmids and pTNS1 to promote transposition and single gene insertion, and each was confirmed as described (*30*). The *X. translucens* pv. translucens UPB886 and *X. oryzae* pv. oryzae BXO43 Δ*cbsA* mutants were created using *sacB* counter-selection with the vector pK18mobsacB. The upstream and downstream regions of *cbsA* were amplified using the primers *cbsA* up F/R and *cbsA* down F/R for *X. translucens* pv. translucens and *X. oryzae* pv. oryzae (table S8). Upstream and downstream fragments were either fused and cloned into pK18mobsacB by Gibson Assembly (New England Biolabs, Ipswich, MA, USA) following the manufacturer’s recommendations or cloned by traditional restriction enzyme digestion and ligation, respectively (*12*, *31*). The construct was inserted into the target strain (*X. translucens* pv. translucens UPB886 or *X. oryzae* pv. oryzae BXO43) using electroporation as previously described (*12*, *15*), and the first genomic recombination event was selected on NA + Km. A second recombination event was screened for sucrose and Km sensitivity on NA + 10% sucrose, and the *cbsA* deletion was confirmed using PCR (table S8). We were unable to insert *cbsA* via miniTn*7* into the ∆*cbsA* mutant of *X. translucens* pv. translucens strain UPB886 for complementation. We therefore sequenced *X. translucens* pv. translucens ∆*cbsA* with long-read PacBio sequencing. There were no notable differences in sequence between WT UPB886 and the ∆*cbsA* mutant beyond the absence of *cbsA* (fig. S9). For visualization of bacteria by fluorescence microscopy, *Xanthomonas* bacteria (table S7) were transformed with vectors for GFP expression (pNEO-GFP) (*32*). See tables S7 and S8 for specific strains and primers, respectively, used in this study.

The deletion of *cbsA* in *X. fastidiosa* strain TemeculaL (locus ID PD0529) was performed as described elsewhere (*29*). Briefly, to obtain the targeting construct for site-directed mutagenesis, the upstream and downstream regions (905 and 968 base pairs, respectively) immediately flanking the *cbsA* gene were amplified using pairs of primers containing overlapping nucleotides with the Km resistance cassette present in the pUC4K plasmid (tables S7 and S8). The upstream and downstream regions of *cbsA* were fused to the Km resistance cassette through overlap-extension PCR, as detailed in (*29*). The purified PCR product was used for transforming WT strain through natural competence directly. Briefly, *X. fastidiosa* TemeculaL cells were suspended to OD_600_ of 0.25 (~10^8^ cells/ml) in PD3 broth (*29*), and 10 μl of this suspension was spotted together with 10 μl of the targeting construct on a PD3 agar plate. After 5 days of growth at 28°C, cells were suspended into 1 ml of PD3 broth and plated into PW + Km agar for selection of mutants obtained through homologous recombination. Successful deletion of *cbsA* was confirmed through PCR (primers shown in table S8). In summary, nonamplification of an internal sequence of *cbsA*, and amplification of an internal sequence of the upstream region and the Km resistance cassette, confirmed deletion of *cbsA* in *X. fastidiosa* TemeculaL (WT was included as control). The obtained Δ*cbsA* mutant was stored as 25% glycerol stocks at −80°C until use. The Gel/PCR DNA Fragments Extraction Kit (IBI Scientific) was used for purification of PCR products and agarose gel fragments when needed. PCRs were performed using a standard protocol with the iProof High-Fidelity PCR Kit (Bio-Rad) in an S1000 thermal cycler (Bio-Rad).

### Plant growth conditions, inoculation methods, and live imaging with confocal microscopy

Barley (*Hordeum vulgare* L. cv. Morex) were grown in growth chambers with cycles of 16 hours of light per day at 22° to 24°C. Rice (*Oryza sativa*) were grown in growth chambers with 16 hours of light per day at 28°C 70% relative humidity or in the greenhouse. Plant seeds were directly germinated in potting mix. For barley, one leaf per plant was inoculated by leaf-clipping 7 to 10 days after seeds were planted with a water-based inoculum (OD_600_ = 0.1) or water as a control as previously described (*15*, *33*). For rice, one leaf per Nipponbare plants (3 weeks old) was clip-inoculated with *X. oryzae* or mutant (resuspension in water of OD_600_ = 0.1). Disease symptoms were assessed using at least five replications per condition with each experiment, and each experiment was repeated at least three times. Symptom development was evaluated 21 or 15 days post-inoculation (dpi), respectively. For evaluation of enhanced water soaking adjacent to lesions in *X. translucens* pv. translucens ∆*cbsA* mutant compared to WT UPB886, symptoms were measured at 14 dpi. Statistical significance was evaluated using an analysis of variance (ANOVA) or Student’s *t* test.

For *X. fastidiosa* inoculation, plants were inoculated with PBS buffer (*n* = 6), *X. fastidiosa* WT (*n* = 9), or Δ*cbsA* (*n* = 9). Disease severity and disease incidence were recorded weekly for 10 time points after first symptom appearance (8 weeks after inoculation). Briefly, disease incidence was considered as the percentage of plants showing at least one symptomatic leaf out of the total plants inoculated. Disease severity was calculated for each plant by counting symptomatic leaves and total number of leaves [(symptomatic leaves/total leaves) × 100] for each plant. The area under the disease progress curve (AUDPC) was calculated by the midpoint rule method (Campbell and Madden 1990): AUDPC = Σ *in* − 1 [(*yi* + *yi* + 1)/2] (*ti* + 1 − *ti*), where *n* = number of times disease assessment was performed, *y* = score of severity for each plant, and *t* = time of assessment.

For bacterial localization, barley plant leaves were inoculated as above. Whole-leaf tissue was imaged 5 to 14 dpi with a Leica SP2 AOBS (Wetzlar, Germany) laser scanning confocal microscope with ×40 oil objective. Barley leaves were cut directly adjacent to the inoculation zone for asymptomatic plants and immediately downstream of symptoms for symptomatic plants. Plant tissue was mounted onto a glass slide with water and covered with a glass coverslip. A 488-nm laser was used for GFP excitation, and emitted fluorescence was collected between 505 and 540 nm. The 405- and 633-nm lasers were used for autofluorescence, and emitted fluorescence was collected between 410 and 460 nm to define plant cell structures and between 650 and 700 nm for chlorophyll. Three to six plants were examined per biological replicate per treatment over three total biological replicates. Representative confocal images represent maximal projections calculated from 15 to 25 confocal planes acquired in the *z* dimension (increments of at least 0.5 mm).

### Phylogenetic analyses

To decrease redundancy among strain- or species-specific genomes in our dataset while maintaining sample power, we built a preliminary 50% majority-rule consensus tree based on the maximum likelihood (ML) phylogenies of 139 amino acid alignments of single-copy orthologs. We used this tree to guide our selection of at most 3 representative genomes from each *Xanthomonas* pathovar, ultimately arriving at a final dataset of 86 genomes (table S1). Using this de-replicated genomic dataset, we then built a final 50% majority-rule consensus tree based on 81–amino acid–based ML phylogenies of single-copy orthologs that had greater than 60% average bootstrap support, our rationale being that consolidating multiple gene trees with high support increases the robustness of species-level phylogenetic inference (*34*). We rooted the final consensus tree at the bifurcation between the beta- and gammaproteobacteria.

All nucleotide and amino acid alignments were generated using MAFFT v7.047 with options “--auto” for automatic selection of best alignment strategy (*35*) and trimmed using trimAL v1.4 with options “-automated1” for heuristic method selection and “-gt 0.25” for removing all sites with gaps in ≥75% of sequences (*36*). Sequences with gaps in ≥30% of sites were removed. All ML trees were built using IQTREE v1.6.9 with option “-m MFP” to find the best-fitting model of sequence evolution (*37*). Majority-rule consensus trees were built using RAxML v8.2.11 (*38*).

### Analysis of *cbsA* homologs and neighboring genomic regions

To determine the precise mechanisms and relative order through which *cbsA* was gained and lost from the genomes in our dataset, we analyzed the evolutionary history and structural features of all gene neighborhoods that flank *cbsA*. Using custom scripts, we first explored the gene neighborhoods surrounding *cbsA* homologs (±15 kb) in the 86-genome dataset for conserved synteny, as defined by orthogroup content conservation. For each of the four conserved neighborhood types that we identified, we then re-searched all genomes for regions composed of these genes, thus identifying all instances of each neighborhood in each genome, regardless of whether *cbsA* was present or not (table S3). In doing so, we could then leverage phylogenetic evidence from flanking genes to support or reject competing hypotheses of gene duplications, HGTs, and losses that may have resulted in *cbsA*’s extant distribution.

We built nucleotide-based ML phylogenies of *cbsA* and the genes from each neighborhood type and manually reconciled their evolutionary histories with the consensus species tree using a combination of parsimony-based gene tree-species tree reconciliation and likelihood-based phylogenetic testing (figs. S6 to S8 and tables S4 to S6). To robustly root the *cbsA* tree for reconciliation analysis, we first retrieved the top 1000 hits in the National Center for Biotechnology Information (NCBI) nr protein database (last accessed: 3 September 2018) to the *cbsA* sequence in *X. campestris* (accession: WP_076057318) and used them to build a midpoint rooted ML tree (available on the Figshare repository: 10.6084/m9.figshare.8218703). This tree was then used as a reference to root subsequent ML trees that focused only on this study’s clade of *cbsA* sequences of interest. We additionally built an ML tree with *cbsA* sequences from the full 179 genome dataset to verify the final topology of the *cbsA* tree built with the 86 genome de-replicated dataset (available on the Figshare repository: 10.6084/m9.figshare.8218703). All other gene trees were midpoint rooted.

All genomic regions were further annotated for transposable elements with BLAST using the ISFinder database to ensure a comprehensive structural annotation of mobile elements (*39*). Nucleotide sequences of the genomic regions that were missing *cbsA* were searched using BLASTn with a *cbsA* query to ensure that any missing or incomplete *cbsA* coding regions were identified. The mixture model and hidden Markov model from the PhyML package were used to detect homologous recombination breakpoints in the untrimmed nucleotide alignments that were then manually inspected and refined if necessary (*40*).

### Phylogenetic hypothesis testing

In each tree with a topology that suggested HGT, we compared the likelihood of the most likely tree obtained through a standard ML search (representing the hypothesis of HGT) with the likelihood of a constrained tree where sequences were forced to adhere to a topology that would be expected under a scenario of vertical inheritance (representing the hypothesis of no HGT). In this way, we could probabilistically assess whether a scenario of vertical inheritance or HGT best explained the observed sequence data. We used the approximately unbiased (AU) test with 100,000 resamplings using the RELL method (*41*) as implemented in IQTREE v1.6.9 (*37*) to identify the most likely tree among a set of constrained and optimal trees. The null hypothesis that the constrained tree had the largest observed likelihood was rejected at α ≤ 0.05. Practically, this meant that we inferred HGT by showing that the constrained ML tree was significantly worse (smaller log likelihood) than the optimal ML tree. Constrained ML tree searches were conducted using IQTREE v1.6.9 (*37*) by supplying a trimmed nucleotide alignment and a noncomprehensive, multifurcating constraint tree specifying the monophyly of particular sequences of interest to which the resulting ML tree was forced to adhere to (figs. S4 to S6; see tables S4 to S6 for all constraint criteria).

### Data visualization

All phylogenetic trees were visualized using ETE3 v3.0.0b32 (*42*). All genomic regions were visualized using Easyfig (*43*).

## Supplementary Material

http://advances.sciencemag.org/cgi/content/full/6/46/eabc4516/DC1

Tables S1 to S8

Adobe PDF - abc4516_SM.pdf

Adobe PDF - abc4516_Figure_S7.pdf

Adobe PDF - abc4516_Figure_S8.pdf

Repeated gain and los of a single gene modulates the evolution of vascular plant pathogen lifestyles

## SUPPLEMENTARY MATERIALS

Supplementary material for this article is available at http://advances.sciencemag.org/cgi/content/full/6/46/eabc4516/DC1

## Appendix

View/request a protocol for this paper from *Bio-protocol*.
