# Supplementary material for: Repeated gain and loss of a single gene modulates the evolution of vascular plant pathogen lifestyles
Source: Sci Adv. 2020 Nov 13;6(46):eabc4516. doi: 10.1126/sciadv.abc4516 (PMC7673761; doi:10.1126/sciadv.abc4516)
Supplement: http://advances.sciencemag.org/cgi/content/full/6/46/eabc4516/DC1 [file supp_6_46_eabc4516__index.html]

Science Advances | Science AdvancesAAASSearchScience AdvancesMenu

## Supplementary Materials

# Repeated gain and loss of a single gene modulates the evolution of vascular plant pathogen lifestyles

Emile Gluck-Thaler, Aude Cerutti, Alvaro L. Perez-Quintero, Jules Butchacas, Verï¿½nica Roman-Reyna, Vishnu Narayanan Madhavan, Deepak Shantharaj, Marcus V. Merfa, Cï¿½line Pesce, Alain Jauneau, Taca Vancheva, Jillian M. Lang, Caitilyn Allen, Valerie Verdier, Lionel Gagnevin, Boris Szurek, Gregg T. Beckham, Leonardo De La Fuente, Hitendra Kumar Patel, Ramesh V. Sonti, Claude Bragard, Jan E. Leach, Laurent D. Noï¿½l, Jason C. Slot, Ralf Koebnik, Jonathan M. Jacobs

Download Supplement

**The PDF file includes:**

- Supplementary Materials and Methods
- Figs. S1 to S6 and S9
- Legends for figs. S7 and S8
- References

**Other Supplementary Material for this manuscript includes the following:**

- Fig. S7
- Fig. S8
- Tables S1 to S8

**Files in this Data Supplement:**

- Adobe PDF - abc4516\_SM.pdf
- Adobe PDF - abc4516\_Figure\_S7.pdf
- Adobe PDF - abc4516\_Figure\_S8.pdf
- abc4516\_Tables\_S1\_to\_S8.xlsx
