# Supplementary material for: Repeated gain and loss of a single gene modulates the evolution of vascular plant pathogen lifestyles
Source: Sci Adv. 2020 Nov 13;6(46):eabc4516. doi: 10.1126/sciadv.abc4516 (PMC7673761; doi:10.1126/sciadv.abc4516)
Supplement: http://advances.sciencemag.org/cgi/content/full/6/46/eabc4516/DC1 [file supp_6_46_eabc4516__1.pdf]

Science Advances
AAAS

advances.sciencemag.org/cgi/content/full/6/46/eabc4516/DC1

Supplementary Materials for

Repeated gain and loss of a single gene modulates the evolution of vascular plant pathogen lifestyles

Emile Gluck-Thaler, Aude Cerutti, Alvaro L. Perez-Quintero, Jules Butchacas, Verónica Roman-Reyna, Vishnu Narayanan Madhavan, Deepak Shantharaj, Marcus V. Merfa, Céline Pesce, Alain Jauneau, Taca Vancheva, Jillian M. Lang, Caitilyn Allen, Valerie Verdier, Lionel Gagnevin, Boris Szurek, Gregg T. Beckham, Leonardo De La Fuente, Hitendra Kumar Patel, Ramesh V. Sonti, Claude Bragard, Jan E. Leach, Laurent D. Noël, Jason C. Slot, Ralf Koebnik*, Jonathan M. Jacobs*

*Corresponding author. Email: jacobs.1080@osu.edu (J.M.J.); koebnik@gmx.de (R.K.)

Published 13 November 2020, *Sci. Adv.* **6**, eabc4516 (2020)
DOI: 10.1126/sciadv.abc4516

The PDF file includes:

Figs. S1 to S6 and S9
Legends for figs. S7 and S8
References

Other Supplementary Material for this manuscript includes the following:

(available at advances.sciencemag.org/cgi/content/full/6/46/eabc4516/DC1)

Tables S1 to S8

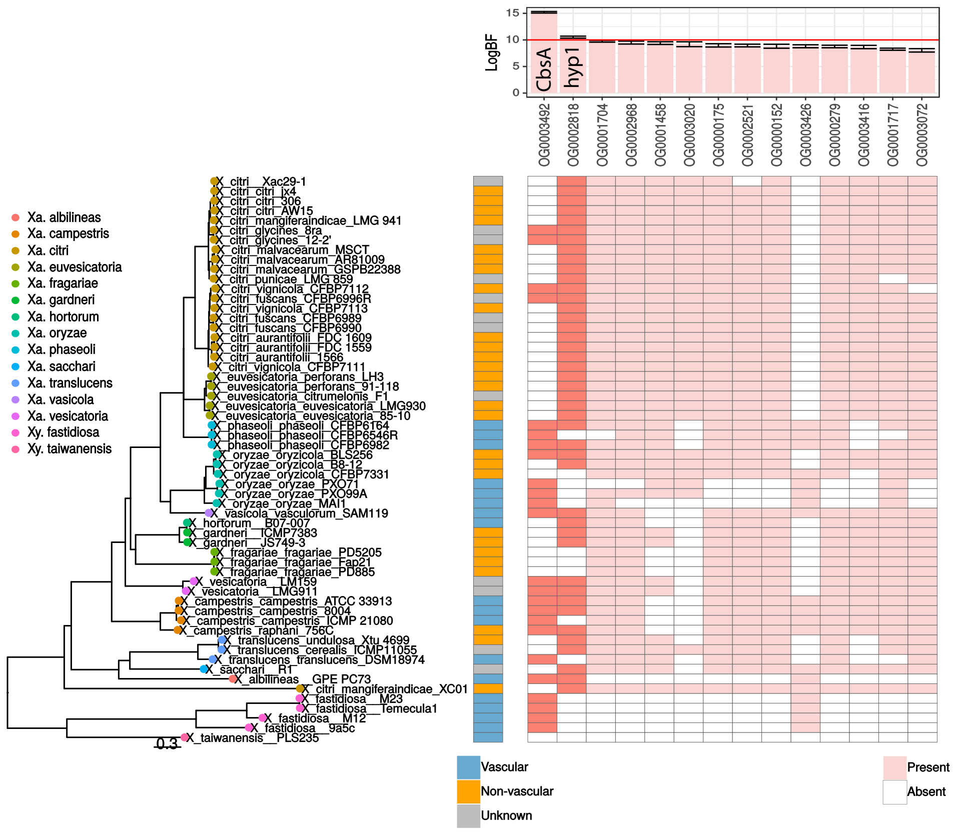

Figure S1. Evolutionary relationship between vascular pathogenesis and a conserved cell wall-degrading enzyme in *Xanthomonas* bacteria. This figure is a modified Fig. 1 with detailed strain information for genomes analyzed. To explore the association of the vascular/non-vascular lifestyle a set of publicly available complete and annotated genomes from different species in the *Xanthomonadaceae* family was analyzed. A pan genome SNP-based parsimony tree was built using kSNP3 (optimum kmer size = 21)(*26*). Genomes were classified as vascular (blue), non-vascular (yellow) or unknown (gray) based on available information in the literature. Ortholog groups for all annotated proteins were identified using Orthofinder (*44*), and a parsimony tree was generated based on pan-genome SNPs using KSNP3. Associations were identified between the presence/absence of each orthologue group in the analyzed genomes and the vascular/non-vascular trait according to the phylogeny using BayesTraitsV3. The likelihood that both traits (vascularity vs. gene presence) evolved dependently was compared to the likelihood they evolved dependently. Evidence of dependent evolution was assessed as Log Bayes Factors = 2(log marginal likelihood dependent model – log marginal likelihood independent model). Gene groups that were determined to evolve dependent on vascularity with very strong evidence (logBF >10; dark red) are shown, as well as the next top 12 genes below the threshold (light red), genes are marked in red when present in a given strain. One gene group (OG0003492; CbsA) was commonly found in vascular strains, and the other (OG0002818; hypothetical) was more common in non-vascular genomes.

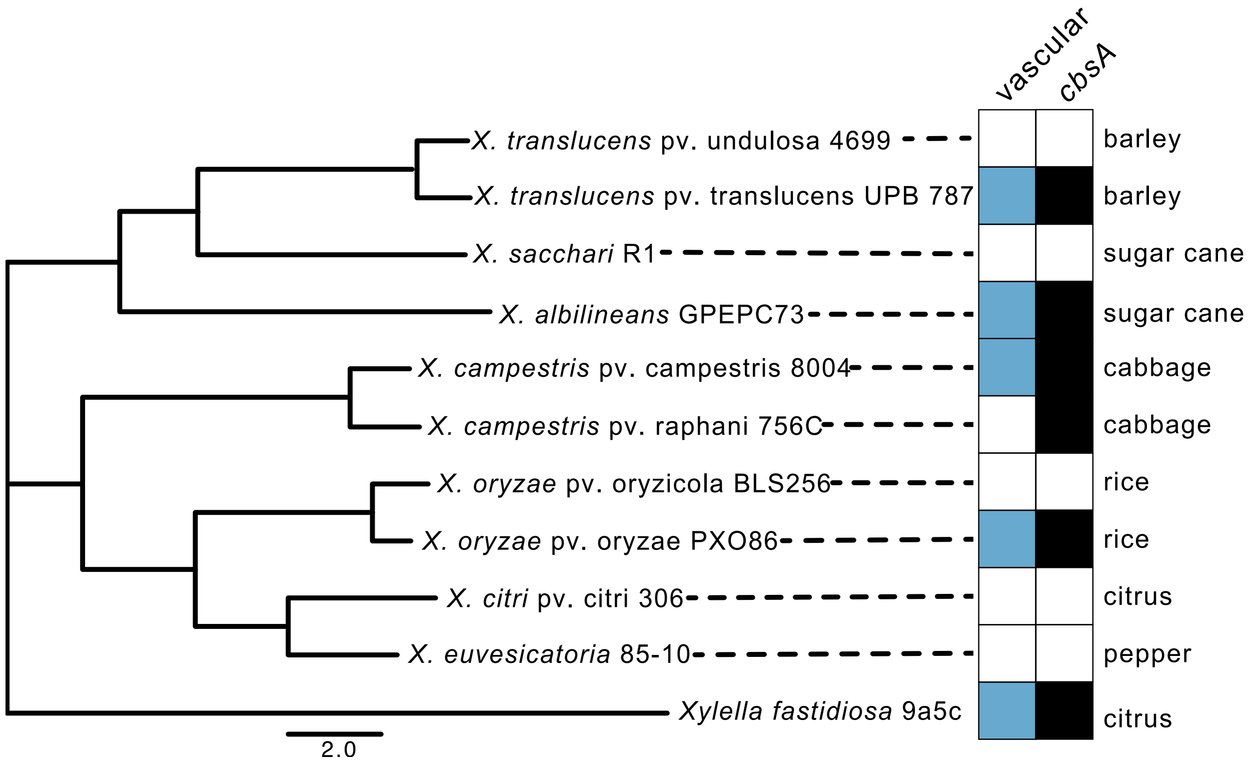

Figure S2. Vascular, xylem pathogenesis strongly correlates with presence of *cbsA* but not host species. A phylogenetic tree was created based on representative xanthomonad genomes from NCBI with Average Nucleotide Identity (ANI) (http://enve-omics.ce.gatech.edu/g-matrix/). Vascular, xylem-colonizing bacteria are denoted in blue. Black boxes identify genomes with a *cbsA* homolog. Primary, characterized host for each pathogen is listed to the right of the boxes.

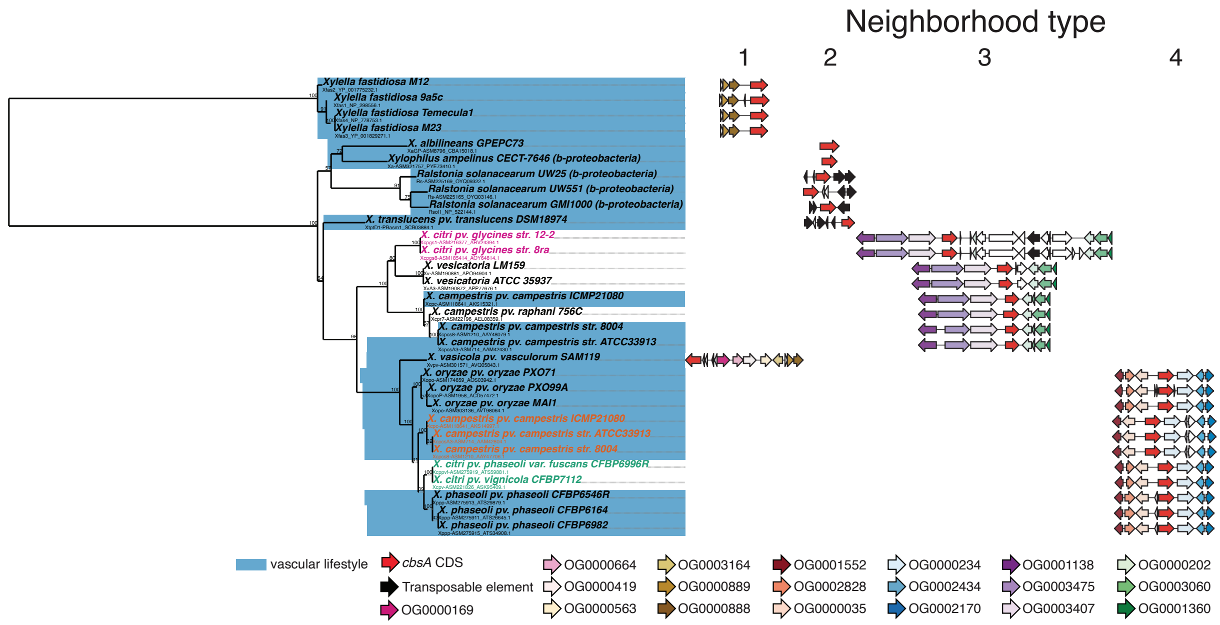

Figure S3. The evolution and genomic context of *cbsA*. To the left is a nucleotide-based maximum likelihood phylogeny of *cbsA* homologs retrieved from the genome database from this paper (see Table S1). Bootstrap support values (out of 100) are indicated above each bipartition. Each tip of the tree lists the full name of the isolate from which the sequence was retrieved in addition to the sequence's accession number. To the right are schematics of the four distinct types of gene neighborhoods in which *cbsA* sequences are found. The colored species names (fuchsia, green and orange) signify horizontal transfer events. All schematics are drawn to scale within each column. Genes belonging to orthogroups of interest are color-coded (see legend at bottom), while all other intervening genes are left blank.

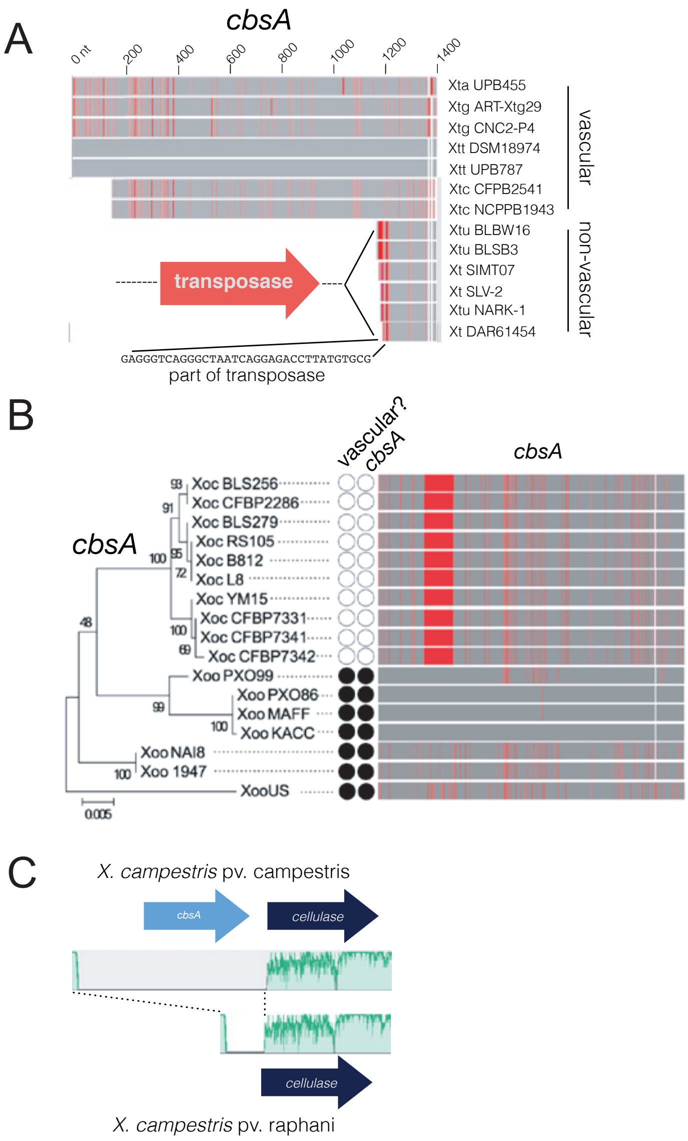

Figure S4. Specific inactivation events for *cbsA* homologs in *Xanthomonas* spp. *cbsA* genes or genomic regions were aligned from vascular and non-vascular A) *Xanthomonas translucens*, B) *Xanthomonas oryzae* and C) *Xanthomonas campestris* with A&B) MAFFT alignment (www.benchling.com) and C) MAUVE. A&B) Gray and red signify the same or different respective nucleotide in the alignment. *cbsA* homologs were independently interrupted by three independent events: A) insertion, B) small deletion and C) complete gene loss. B) For *Xanthomonas oryzae* pathovars, the presence of CbsA (black circles) was strongly correlated with vascular (black circles) *X. oryzae* pv. oryzae genomes but absent from non-vascular (white circles) *X. oryzae* pv. oryzae. C) *X. campestris* pv. campestris is vascular, while *X. campestris* pv. raphani is non-vascular. Green signifies level of identity between a given nucleotide sequences.

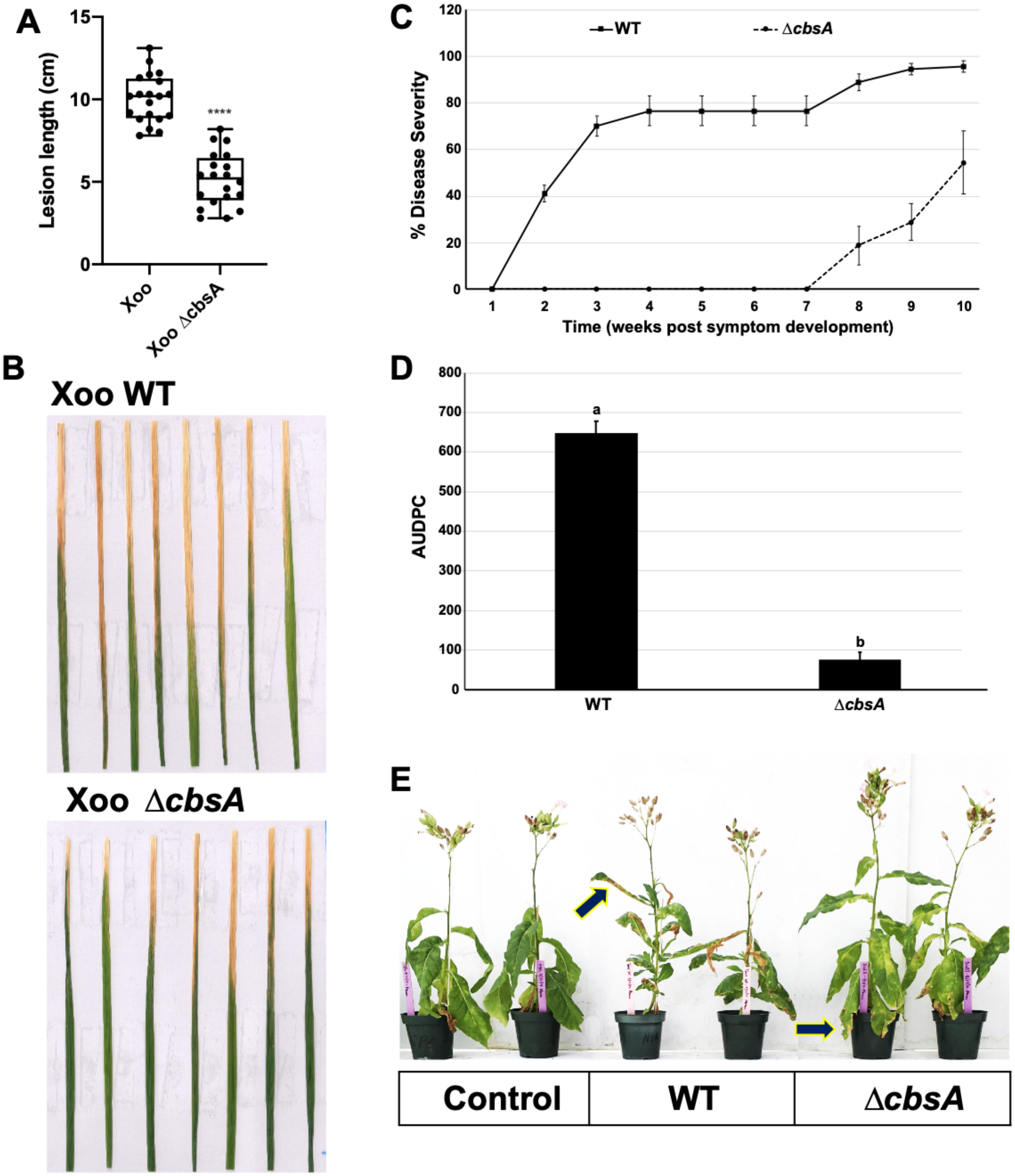
A
Lesion length (cm)
15
10
5
0
****
Xoo
Xoo ΔcbsA
B
Xoo WT
Xoo ΔcbsA
C
WT
ΔcbsA
% Disease Severity
120
100
80
60
40
20
0
1 2 3 4 5 6 7 8 9 10
Time (weeks post symptom development)
D
AUDPC
800
700
600
500
400
300
200
100
0
a
b
WT
ΔcbsA
E
Control
WT
ΔcbsA

Figure S5. Mutation of *cbsA* negatively affects virulence in *X. oryzae* pv. oryzae and *Xylella fastidiosa.* A) Rice plants (cv. Nipponbare) were inoculated with *X. oryzae* pv. oryzae wild-type or Δ*cbsA* mutant. A-B) Lesion lengths were measured and imaged 15 days post inoculation (Photo Credit: V. Narayanan Madhavan, CSIR). Lesion length was compared with Student's *t*-test ($P<0.0001$). C-E) Disease severity progression over time in inoculated tobacco plants. *Xylella fastidiosa* subsp. *fastidiosa* strain TemeculaL (WT) and mutant Δ*cbsA* were inoculated into *Nicotiana tabacum* L. cv. Petit Havana SR1 plants (PBS mock inoculation used as control). Leaf scorch symptoms were recorded for measurements of disease incidence and severity once a week during ten weeks after appearance of the first disease symptoms. At the final time point of evaluation, disease incidence in TemeculaL WT reached 100%, compared to mutant Δ*cbsA* reaching 66%; while disease severity reached 95% in WT and 54% in Δ*cbsA*. The mutant Δ*cbsA* showed delay of leaf scorch symptom development, with symptom appearance at the seventh week onwards and mostly restricted to lower leaves close to the inoculation point. Data represent means and standard errors from one experiment (n=9 for WT and Δ*cbsA*). D) Mean AUDPC per treatment group (WT and Δ*cbsA*). AUDPC was calculated using data from disease severity over ten weeks after first disease symptom appearance. AUDPC was lower for plants inoculated with Δ*cbsA*, in comparison to WT-inoculated plants. Data represent means and standard errors. Statistical significance was calculated using Tukey-Kramer HSD ($P<0.05$) (Statistical software JMP 15.0.1, 2015 SAS Inst. Inc., Cary, NC). E) Representative image of leaf scorch symptoms in WT- and Δ*cbsA*-inoculated plants, as well as control plants (PBS-inoculated; Photo Credit: D. Shantharaj, Auburn). Arrows in figures point to symptomatic leaves, which were distributed throughout the entire plant in WT-inoculated plants, and were mainly restricted to basal and middle leaves in Δ*cbsA*-inoculated plants.

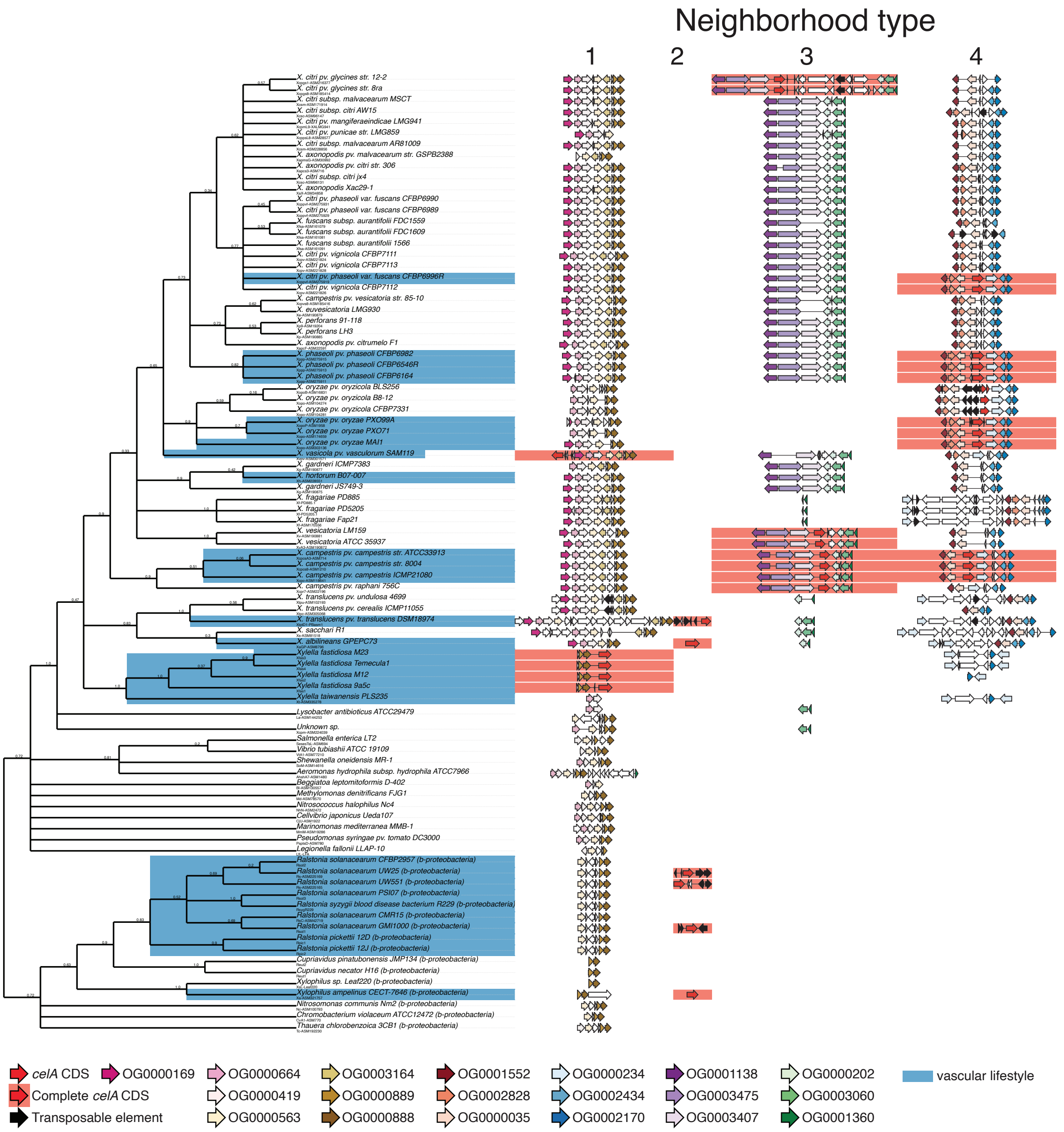

Figure S6. The distribution of *cbsA* loci across beta- and gamma-proteobacteria (unedited version of Figure 1). Shown to the left is a majority rule consensus tree based on 81 maximum likelihood trees of single copy orthologs that summarizes species relationships among 86 bacteria examined in this study. Each bifurcation in the consensus tree is present in at least 50% of the single copy ortholog trees. Branch support values indicate internode certainty (ranging from 0-1), which quantifies the degree of conflict associated with a given bipartition across all 81 constituent trees. To the right of the tree is a graphic summarizing the distribution of the four distinct neighborhoods in which *cbsA* is found across each genome, in all cases whether *cbsA* is present or not. All neighborhood schematics are drawn to scale within each column. Genes

belonging to orthogroups of interest are color-coded (see legend at bottom), while all other intervening genes are left blank.

See additional file attached (too large for supplemental document).

Figure S7. Mid-point rooted, nucleotide-based maximum likelihood phylogenies of all genes in the type 4 *cbsA* neighborhood. Bootstrap support values (out of 100) are indicated above each bipartition. Each tip of the tree lists the full name of the isolate from which the sequence was retrieved in addition to the sequence's accession number. Tree tips associated with sequences from *X. campestris* are colored orange, while tips associated with sequences from *X. citri* pv. phaseoli*, X. citri* pv. vignicola and *X. fuscans* are colored green. In topologies suggesting horizontal gene transfer (HGT), colored sequences were forced to be monophyletic in order to generate constrained topologies that would be expected under a scenario of vertical inheritance for phylogenetic hypothesis testing (Methods; Tables S4-5). A schematic of a *cbsA* gene neighborhood from *X. oryzae* pv. oryzae strain MAI1 is drawn above each tree, and a black vertical triangle indicates the current gene tree being displayed. Boundaries of the inferred homologous recombination events (Methods) are indicated by dashed lines, and are colored green for the HGT from the *X. phaseoli* clade to *X. campestris* and orange for the HGT from the *X. phaseoli* clade to *X. citri* pv. vignicola CFBP7112 and *X. citri* pv. phaseoli var. fuscans CFBP6996R. a) OG0001552. b) OG0002828. c) OG0000035 and 5' UTR, partition 2. d) OG0000035 and 5' UTR, partition 1. e) OG0000234. f) OG0002434. g) OG0002170.

See additional file attached (too large for supplemental document).

Figure S8. Mid-point rooted, nucleotide-based maximum likelihood phylogenies of all genes in the type 3 *cbsA* neighborhood, with midpoint rooting. Bootstrap support values (out of 100) are indicated above each bipartition. Each tip of the tree lists the full name of the isolate from which the sequence was retrieved in addition to the sequence's accession number. Tree tips associated with sequences from *X. citri* pv. glycines*, X. citri* pv. punicae*, X. citri* subsp. malvacearum*, X. citri* pv. mangiferaeindicae*, X. citri* subsp. citri and *X. axonopodis* pv. citri are colored pink. In topologies suggesting horizontal gene transfer (HGT), colored sequences were forced to be monophyletic in order to generate constrained topologies that would be expected under a scenario of vertical inheritance for phylogenetic hypothesis testing (Methods; Table S6). A schematic of a *cbsA* gene neighborhood taken from *X. citri* pv. glycines *str. 8a* is drawn above each tree, and a black vertical triangle indicates the current gene being viewed. The boundaries of the inferred homologous recombination event (Methods) from *X. vesicatoria* to *X. citri* pv. glycines is indicated by black dashed lines. A black bracket indicates the boundaries of a 9-gene region that was likely inserted into the *cbsA* neighborhood after the HGT event. a) OG0003189. b) OG0003864. c) OG0001138. d) OG0003475. e) OG0003407. f) OG0006923. g) OG0000202. h) OG0012116. i) OG0006653. j) OG0004064. k) OG0005040. l) OG0015184. m) OG0004674. n) OG0005551. o) OG0003060. p) OG0001360. q) OG0000926. r) OG0002126. s) OG0001639. t) OG0001483. u) OG0001080. v) OG0000801, partition 2. w) OG0000801, partition 1. x) OG0001155. y) OG0001453.

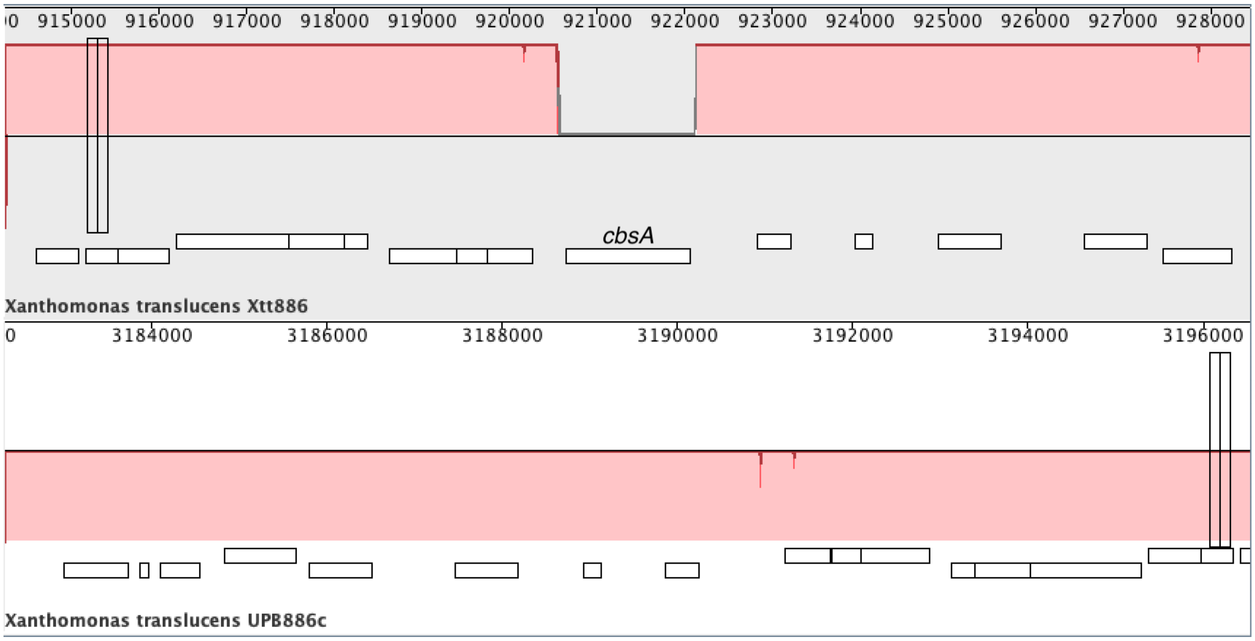

Fig. S9. Complete, whole genome sequencing validation of *X. translucens* pv. translucens Δ*cbsA*. We were unable to create a miniTn*7*::*cbsA* complementation of *X. translucens* pv. translucens UPB886 by transformation or conjugation. Therefore, we performed whole genome sequencing to define the Δ*cbsA* mutation in UPB886. Genomic DNA from *X. translucens* pv. translucens Δ*cbsA* extracted with QIAGEN Genomic-tips 100G kit and sequenced by Psomagen, Inc using Pacbio RSII 20Kb SMRTbell. Assembly was done using Flye software with the parameters --pacbio-raw –g 5m (*45*). Genome annotation was done with Prokka (*46*). Genome comparisons and variant call was done using Mauve and NUCmer alignments (*47*). A MAUVE genome alignment of wild-type *X. translucens* UPB886 (Xtt886, top) compared to *X. translucens* pv. translucens Δ*cbsA* (UPB886c, bottom) demonstrates that the Δ*cbsA* gene was completely deleted by *sacB* mutagenesis for the *cbsA* loci (*48*, *49*). Red signifies level of homology between sequences with specific open reading frames in boxes below for each genome. The empty gray space above signifies no sequence identity and demonstrates the deletion for the Xtt UPB886 Δ*cbsA* mutant below.

Tables S1-S8. See additional file attached for supplemental tables.

REFERENCES AND NOTES

1. J. Iranzo, Y. I. Wolf, E. V. Koonin, I. Sela, Gene gain and loss push prokaryotes beyond the homologous recombination barrier and accelerate genome sequence divergence. *Nat. Commun.* **10**, 5376 (2019).

2. E. V. Koonin, Y. I. Wolf, Genomics of bacteria and archaea: The emerging dynamic view of the prokaryotic world. *Nucleic Acids Res.* **36**, 6688–6719 (2008).

3. A. T. Maurelli, R. E. Fernández, C. A. Bloch, C. K. Rode, A. Fasano, "Black holes" and bacterial pathogenicity: A large genomic deletion that enhances the virulence of *Shigella* spp. and enteroinvasive *Escherichia coli*. *Proc. Natl. Acad. Sci. U.S.A.* **95**, 3943–3948 (1998).

4. C.-H. Kuo, H. Ochman, Deletional bias across the three domains of life. *Genome Biol. Evol.* **1**, 145–152 (2009).

5. R. A. Melnyk, S. S. Hossain, C. H. Haney, Convergent gain and loss of genomic islands drive lifestyle changes in plant-associated *Pseudomonas*. *ISME J.* **13**, 1575–1588 (2019).

6. S. S. Porter, J. Faber-Hammond, A. P. Montoya, M. L. Friesen, C. Sackos, Dynamic genomic architecture of mutualistic cooperation in a wild population of *Mesorhizobium*. *ISME J.* **13**, 301–315 (2019).

7. K. G. Nandasena, G. W. O'Hara, R. P. Tiwari, J. G. Howieson, Rapid in situ evolution of nodulating strains for *Biserrula pelecinus* L. through lateral transfer of a symbiosis island from the original Mesorhizobial inoculant. *Appl. Environ. Microbiol.* **72**, 7365–7367 (2006).

8. E. A. Savory, S. L. Fuller, A. J. Weisberg, W. J. Thomas, M. I. Gordon, D. M. Stevens, A. L. Creason, M. S. Belcher, M. Serdani, M. S. Wiseman, N. J. Grünwald, M. L. Putnam, J. H. Chang, Evolutionary transitions between beneficial and phytopathogenic *Rhodococcus* challenge disease management. *eLife* **6**, e30925 (2017).

9. J. M. Jacobs, L. Babujee, F. Meng, A. Milling, C. Allen, The in planta transcriptome of *Ralstonia solanacearum*: Conserved physiological and virulence strategies during bacterial wilt of tomato. *MBio* **3**, e00114-12 (2012).

10. M.-A. Jacques, M. Arlat, A. Boulanger, T. Boureau, S. Carrère, S. Cesbron, N. W. G. Chen, S. Cociancich, A. Darrasse, N. Denancé, M. Fischer-Le Saux, L. Gagnevin, R. Koebnik, E. Lauber, L. D. Noël, I. Pieretti, P. Portier, O. Pruvost, A. Rieux, I. Robène, M. Royer, B. Szurek, V. Verdier, C. Vernière, Using ecology, physiology, and genomics to understand host specificity in *Xanthomonas*. *Annu. Rev. Phytopathol.* **54**, 163–187 (2016).

11. D. Barker, M. Pagel, Predicting functional gene links from phylogenetic-statistical analyses of whole genomes. *PLOS Comput. Biol.* **1**, e3 (2005).

12. L. Tayi, S. Kumar, R. Nathawat, A. S. Haque, R. V. Maku, H. K. Patel, R. Sankaranarayanan, R. V. Sonti, A mutation in an exoglucanase of *Xanthomonas oryza*e pv. oryzae, which confers an endo mode of activity, affects bacterial virulence, but not the induction of immune responses, in rice. *Mol. Plant Pathol.* **19**, 1364–1376 (2018).

13. G. T. Beckham, J. Ståhlberg, B. C. Knott, M. E. Himmel, M. F. Crowley, M. Sandgren, M. Sørlie, C. M. Payne, Towards a molecular-level theory of carbohydrate processivity in glycoside hydrolases. *Curr. Opin. Biotechnol.* **27**, 96–106 (2014).

14. C. Bragard, E. Singer, A. Alizadeh, L. Vauterin, H. Maraite, J. Swings, *Xanthomonas translucens* from small grains: Diversity and phytopathological relevance. *Phytopathology* **87**, 1111–1117 (1997).

15. C. Pesce, J. M. Jacobs, E. Berthelot, M. Perret, T. Vancheva, C. Bragard, R. Koebnik, Comparative genomics identifies a novel conserved protein, HpaT, in proteobacterial type III secretion systems that do not possess the putative translocon protein HrpF. *Front. Microbiol.* **8**, 1177 (2017).

16. G. Jha, R. Rajeshwari, R. V. Sonti, Functional interplay between two *Xanthomonas oryzae* pv. oryzae secretion systems in modulating virulence on rice. *Mol. Plant Microbe Interact.* **20**, 31–40 (2007).

17. H. Liu, S. Zhang, M. A. Schell, T. P. Denny, Pyramiding unmarked deletions in *Ralstonia solanacearum* shows that secreted proteins in addition to plant cell-wall-degrading enzymes contribute to virulence. *Mol. Plant Microbe Interact.* **18**, 1296–1305 (2005).

18. J. F. González, G. Degrassi, G. Devescovi, D. De Vleesschauwer, M. Höfte, M. P. Myers, V. Venturi, A proteomic study of *Xanthomonas oryzae* pv. oryzae in rice xylem sap. *J. Proteomics* **75**, 5911–5919 (2012).

19. C. de Azevedo Souza, S. Li, A. Z. Lin, F. Boutrot, G. Grossmann, C. Zipfel, S. C. Somerville, Cellulose-derived oligomers act as damage-associated molecular patterns and trigger defense-like responses. *Plant Physiol.* **173**, 2383–2398 (2017).

20. R. Albalat, C. Cañestro, Evolution by gene loss. *Nat. Rev. Genet.* **17**, 379–391 (2016).

21. A. K. Hottes, P. L. Freddolino, A. Khare, Z. N. Donnell, J. C. Liu, S. Tavazoie, Bacterial adaptation through loss of function. *PLOS Genet.* **9**, e1003617 (2013).

22. B. J. Shapiro, J. Friedman, O. X. Cordero, S. P. Preheim, S. C. Timberlake, G. Szabó, M. F. Polz, E. J. Alm, Population genomics of early events in the ecological differentiation of bacteria. *Science* **336**, 48–51 (2012).

23. C.-L. Huang, P.-H. Pu, H.-J. Huang, H.-M. Sung, H.-J. Liaw, Y.-M. Chen, C.-M. Chen, M.-B. Huang, N. Osada, T. Gojobori, T.-W. Pai, Y.-T. Chen, C.-C. Hwang, T.-Y. Chiang, Ecological genomics in *Xanthomonas*: The nature of genetic adaptation with homologous recombination and host shifts. *BMC Genomics* **16**, 188 (2015).

24. N. Potnis, P. P. Kandel, M. V. Merfa, A. C. Retchless, J. K. Parker, D. C. Stenger, R. P. P. Almeida, M. Bergsma-Vlami, M. Westenberg, P. A. Cobine, L. De La Fuente, Patterns of inter- and intrasubspecific homologous recombination inform eco-evolutionary dynamics of *Xylella fastidiosa*. *ISME J.* **13**, 2319–2333 (2019).

25. E. A. Newberry, R. Bhandari, G. V. Minsavage, S. Timilsina, M. O. Jibrin, J. Kemble, E. J. Sikora, J. B. Jones, N. Potnis, Independent evolution with the gene flux originating from multiple *Xanthomonas* species explains genomic heterogeneity in *Xanthomonas perforans*. *Appl. Environ. Microbiol.* **85**, e00885-19 (2019).

26. D. M. Emms, S. Kelly, OrthoFinder: Solving fundamental biases in whole genome comparisons dramatically improves orthogroup inference accuracy. *Genome Biol.* **16**, 157 (2015).

27. P. Jones, D. Binns, H.-Y. Chang, M. Fraser, W. Li, C. McAnulla, H. McWilliam, J. Maslen, A. Mitchell, G. Nuka, S. Pesseat, A. F. Quinn, A. Sangrador-Vegas, M. Scheremetjew, S.-Y. Yong, R. Lopez, S. Hunter, InterProScan 5: Genome-scale protein function classification. *Bioinformatics* **30**, 1236–1240 (2014).

28. S. N. Gardner, T. Slezak, B. G. Hall, kSNP3.0: SNP detection and phylogenetic analysis of genomes without genome alignment or reference genome. *Bioinformatics* **31**, 2877–2878 (2015).

29. P. P. Kandel, H. Chen, L. De La Fuente, A short protocol for gene knockout and complementation in *Xylella fastidiosa* shows that one of the Type IV pilin paralogs (PD1926) is needed for twitching while another (PD1924) affects pilus number and location. *Appl. Environ. Microbiol.* **84**, e01167-18 (2018).

30. K.-H. Choi, H. P. Schweizer, mini-Tn7 insertion in bacteria with single *att*Tn7 sites: Example *Pseudomonas aeruginosa*. *Nat. Protoc.* **1**, 153–161 (2006).

31. L. Tayi, R. Maku, H. K. Patel, R. V. Sonti, Action of multiple cell wall–degrading enzymes is required for elicitation of innate immune responses during *Xanthomonas oryzae* pv. *oryzae* infection in rice. *Mol. Plant Microbe Interact.* **29**, 599–608 (2016).

32. S.-W. Han, C.-J. Park, S.-W. Lee, P. C. Ronald, An efficient method for visualization and growth of fluorescent *Xanthomonas oryzae* pv. *oryzae in planta*. *BMC Microbiol.* **8**, 164 (2008).

33. B. Yang, A. Bogdanove, Inoculation and virulence assay for bacterial blight and bacterial leaf streak of rice. *Methods Mol. Biol.* **956**, 249–255 (2013).

34. L. Salichos, A. Rokas, Inferring ancient divergences requires genes with strong phylogenetic signals. *Nature* **497**, 327–331 (2013).

35. K. Katoh, D. M. Standley, MAFFT multiple sequence alignment software version 7: Improvements in performance and usability. *Mol. Biol. Evol.* **30**, 772–780 (2013).

36. S. Capella-Gutierrez, J. M. Silla-Martínez, T. Gabaldón, trimAl: A tool for automated alignment trimming in large-scale phylogenetic analyses. *Bioinformatics* **25**, 1972–1973 (2009).

37. L.-T. Nguyen, H. A. Schmidt, A. von Haeseler, B. Q. Minh, IQ-TREE: A fast and effective stochastic algorithm for estimating maximum-likelihood phylogenies. *Mol. Biol. Evol.* **32**, 268–274 (2015).

38. A. Stamatakis, RAxML version 8: A tool for phylogenetic analysis and post-analysis of large phylogenies. *Bioinformatics* **30**, 1312–1313 (2014).

39. P. Siguier, J. Perochon, L. Lestrade, J. Mahillon, M. Chandler, ISfinder: The reference centre for bacterial insertion sequences. *Nucleic Acids Res.* **34**, D32–D36 (2006).

40. B. Boussau, L. Guéguen, M. Gouy, A mixture model and a hidden markov model to simultaneously detect recombination breakpoints and reconstruct phylogenies. *Evol. Bioinform Online* **5**, 67–79 (2009).

41. H. Shimodaira, An approximately unbiased test of phylogenetic tree selection. *Syst. Biol.* **51**, 492–508 (2002).

42. J. Huerta-Cepas, F. Serra, P. Bork, ETE 3: Reconstruction, analysis, and visualization of phylogenomic data. *Mol. Biol. Evol.* **33**, 1635–1638 (2016).

43. M. J. Sullivan, N. K. Petty, S. A. Beatson, Easyfig: A genome comparison visualizer. *Bioinformatics* **27**, 1009–1010 (2011).

44. D. M. Emms, S. Kelly, OrthoFinder: Phylogenetic orthology inference for comparative genomics. *Genome Biol.* **20**, 238 (2019).

45. M. Kolmogorov, J. Yuan, Y. Lin, P. A. Pevzner, Assembly of long, error-prone reads using repeat graphs. *Nat. Biotechnol.* **37**, 540–546 (2019).

46. T. Seemann, Prokka: Rapid prokaryotic genome annotation. *Bioinformatics* **30**, 2068–2069 (2014).

47. G. Marçais, A. L. Delcher, A. M. Phillippy, R. Coston, S. L. Salzberg, A. Zimin, MUMmer4: A fast and versatile genome alignment system. *PLOS Comput. Biol.* **14**, e1005944 (2018).

48. A. C. E. Darling, B. Mau, F. R. Blattner, N. T. Perna, Mauve: Multiple alignment of conserved genomic sequence with rearrangements. *Genome Res.* **14**, 1394–1403 (2004).

49. V. Roman-Reyna, E. K. Luna, C. Pesce, T. Vancheva, C. Chang, J. Ziegle, C. Bragard, R. Koebnik, J. M. Lang, J. E. Leach, J. M. Jacobs, Genome resource of barley bacterial blight and leaf streak pathogen *Xanthomonas translucens* pv. translucens strain UPB886. *Plant Dis.* **104**, 13–15 (2020).
