# Supplementary material for: Repeated gain and loss of a single gene modulates the evolution of vascular plant pathogen lifestyles
Source: Sci Adv. 2020 Nov 13;6(46):eabc4516. doi: 10.1126/sciadv.abc4516 (PMC7673761; doi:10.1126/sciadv.abc4516)

a) OG0003189 (carboxylesterase)

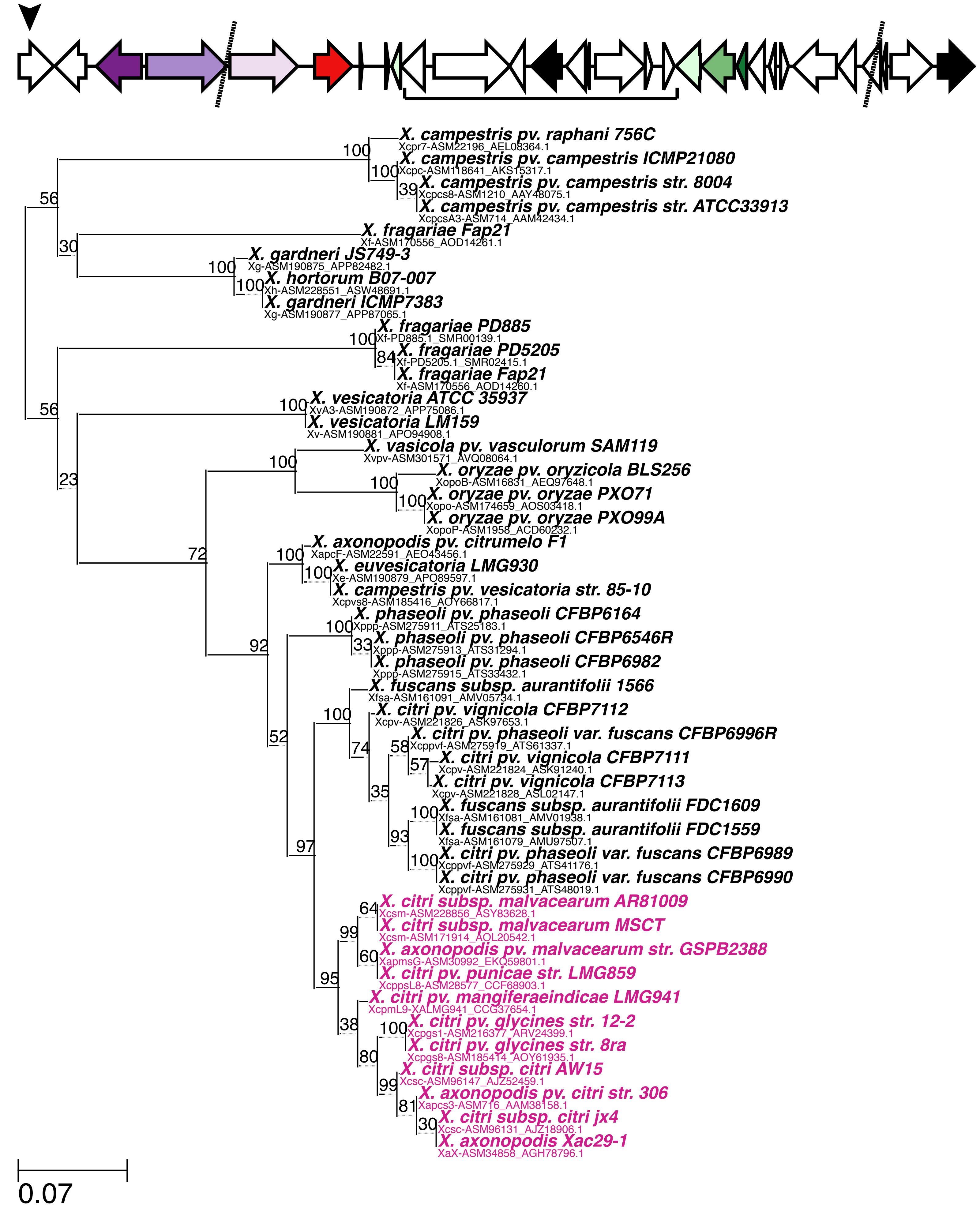

b) OG0003864 (function unknown)

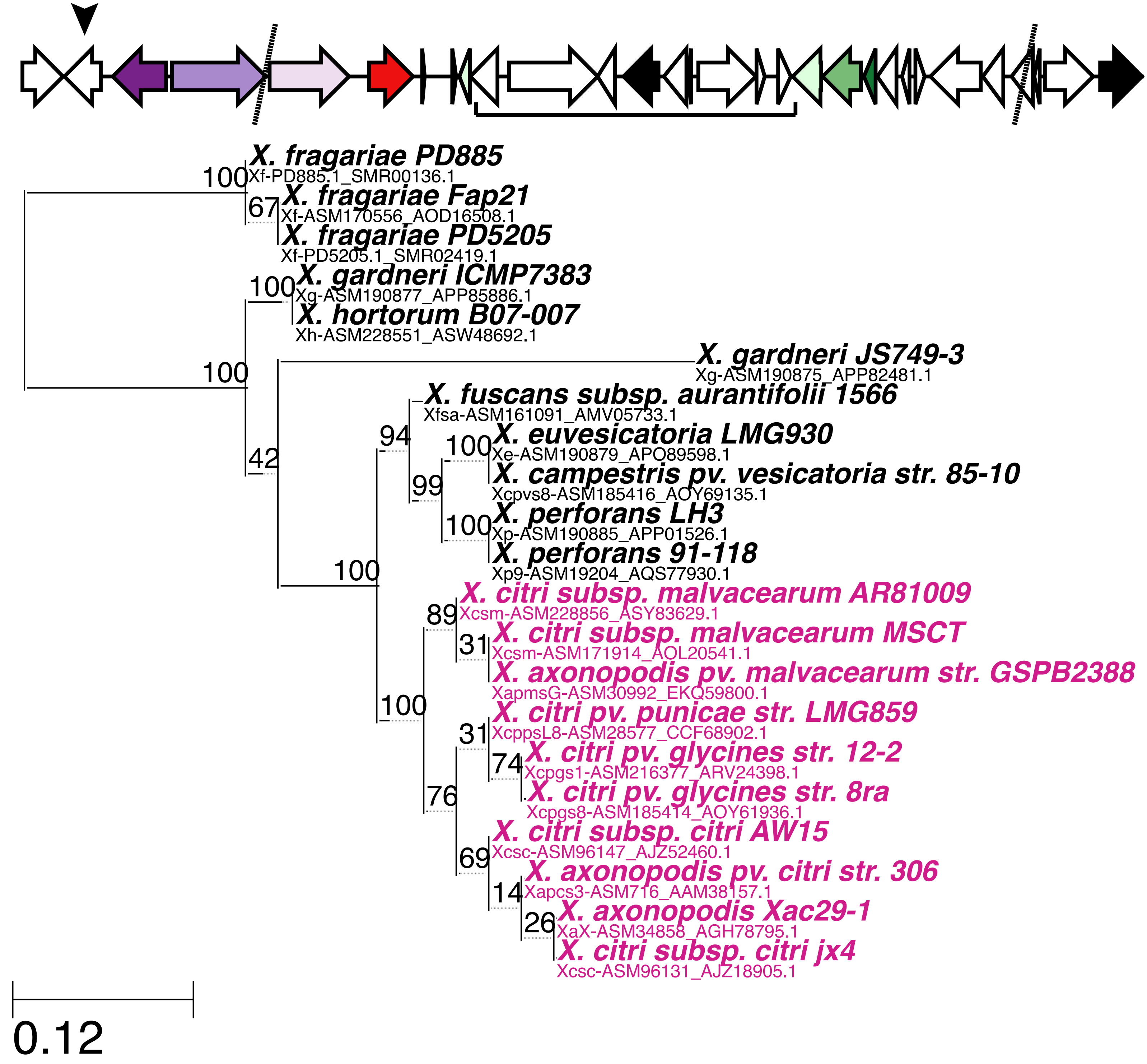

c) OG0001138 (alpha-glucosidase)

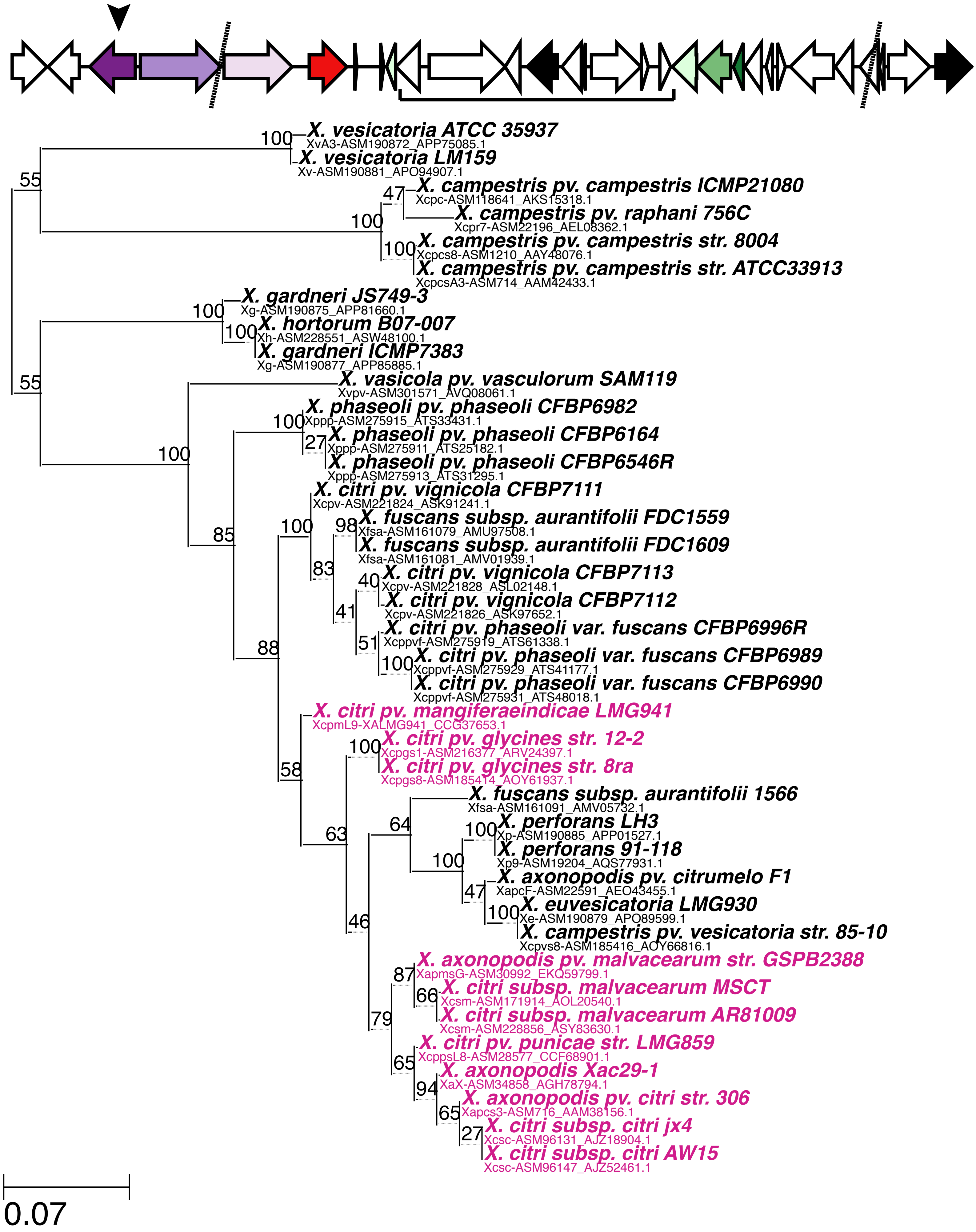

d) OG0003475 (hydrolase family 2, sugar binding)

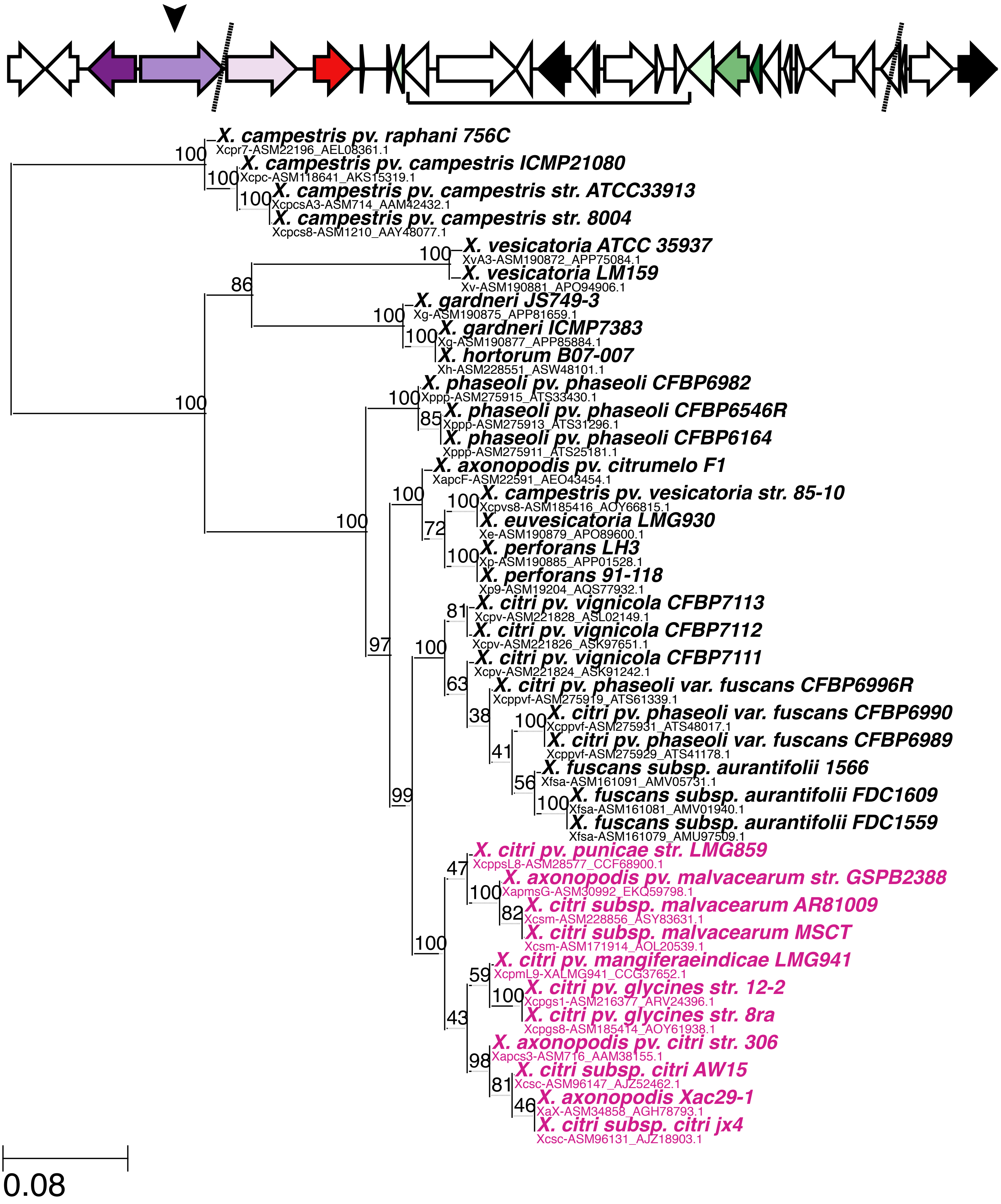

e) OG0003407 (tonB-dependent receptor)

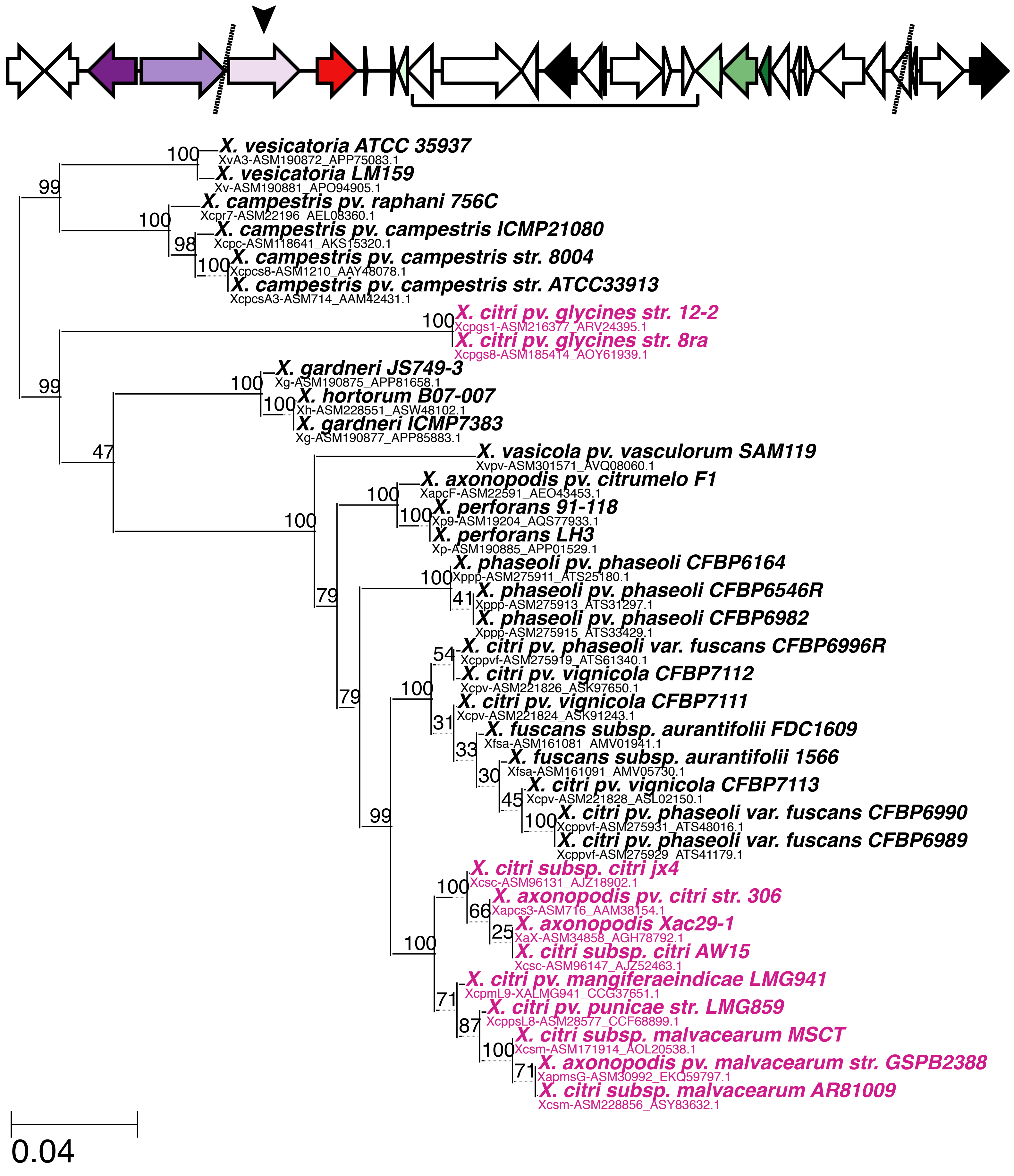

f) OG0006923 (cytochrome p450 domain-containing protein)

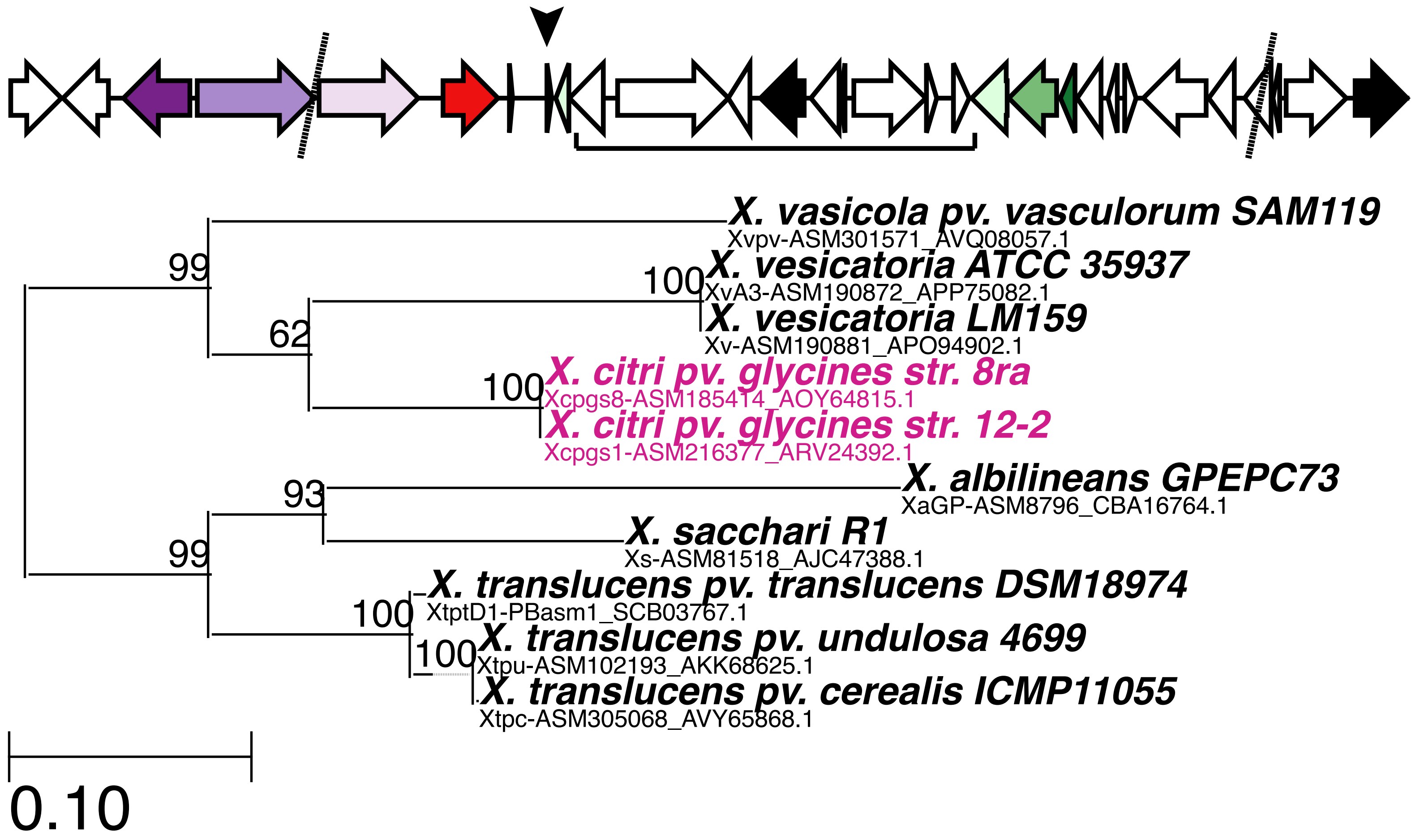

g) OG0000202 (transcription regulator, LacI family)

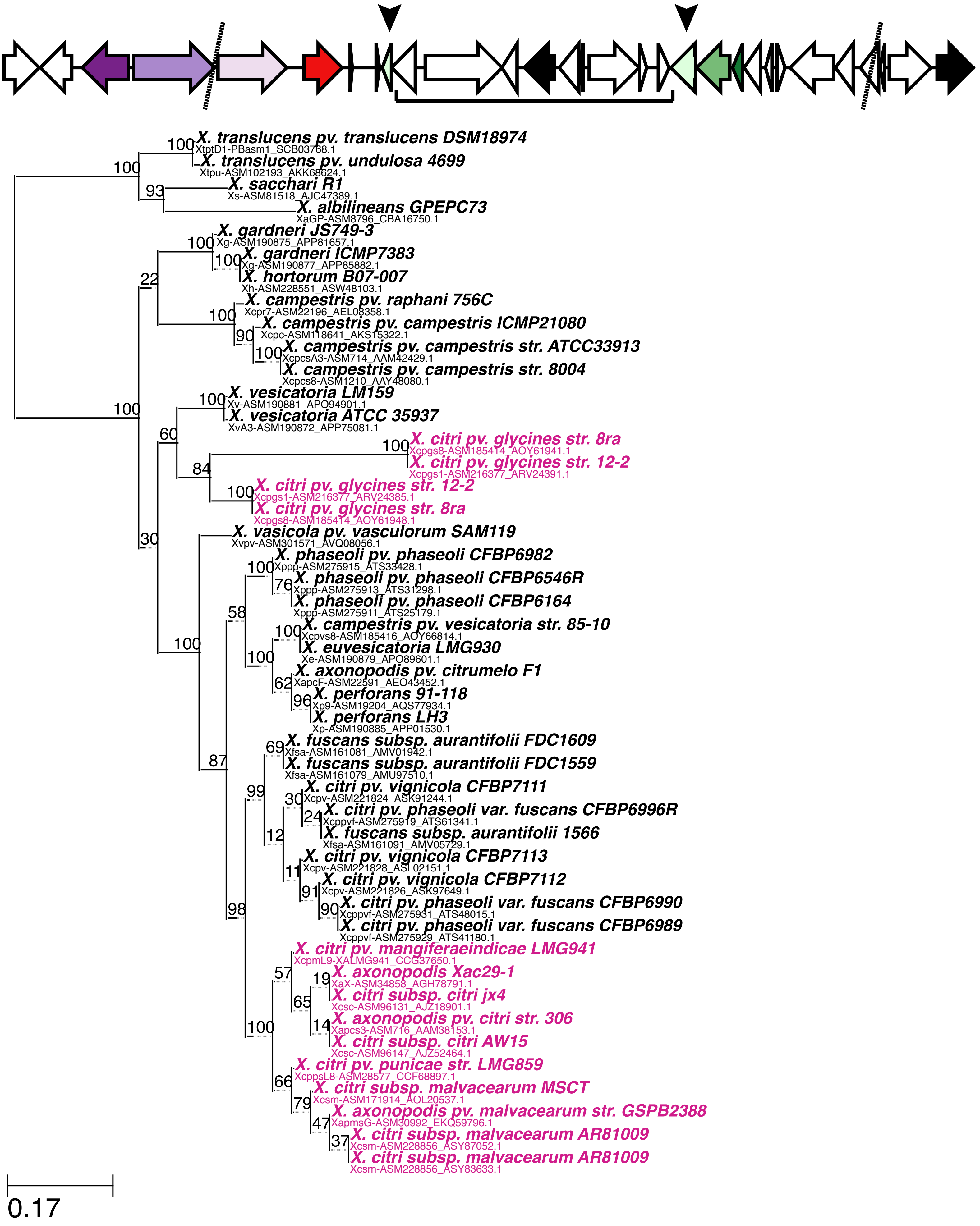

h) OG0012116 (response regulator)

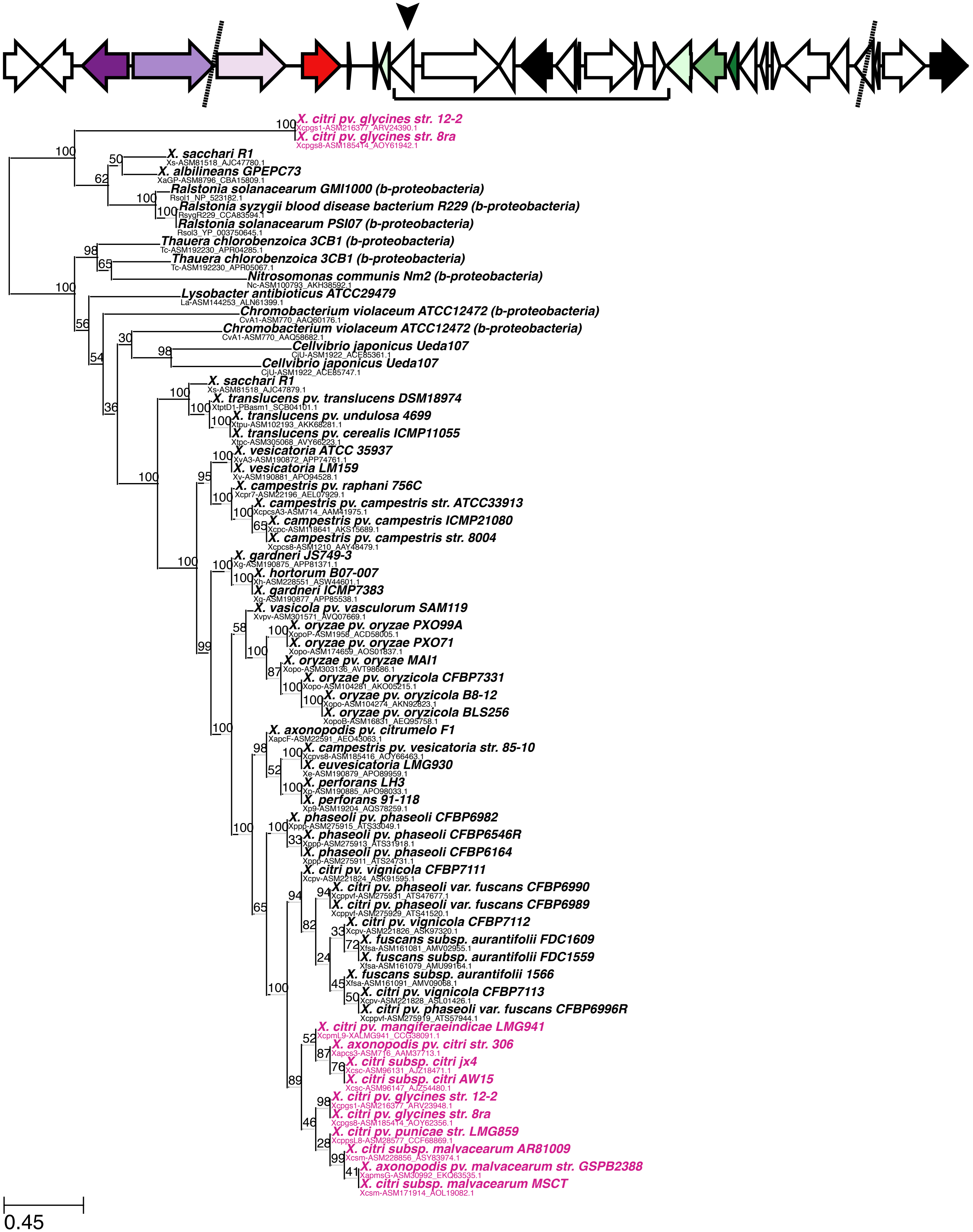

i) OG0006653 (histidine kinase)

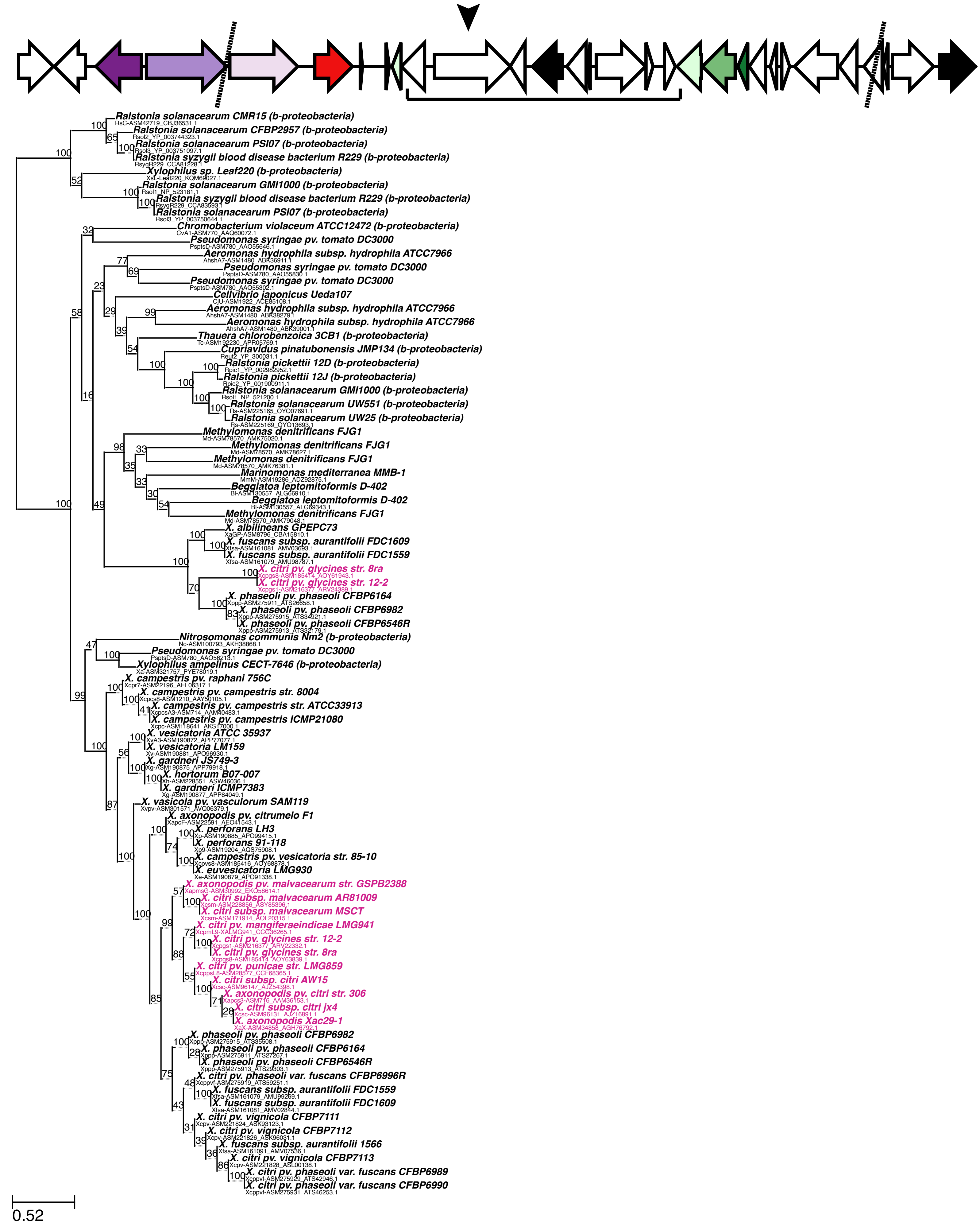

j) OG0004064 (response regulator)

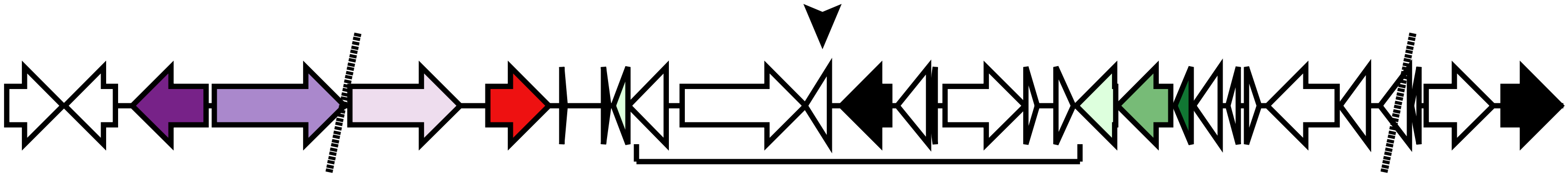

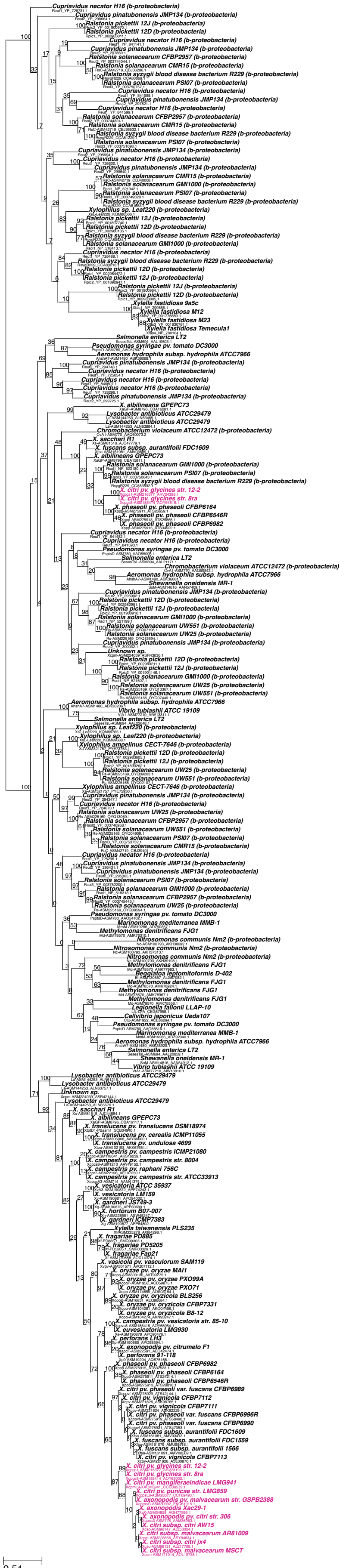

k) OG0005040 (function unknown)

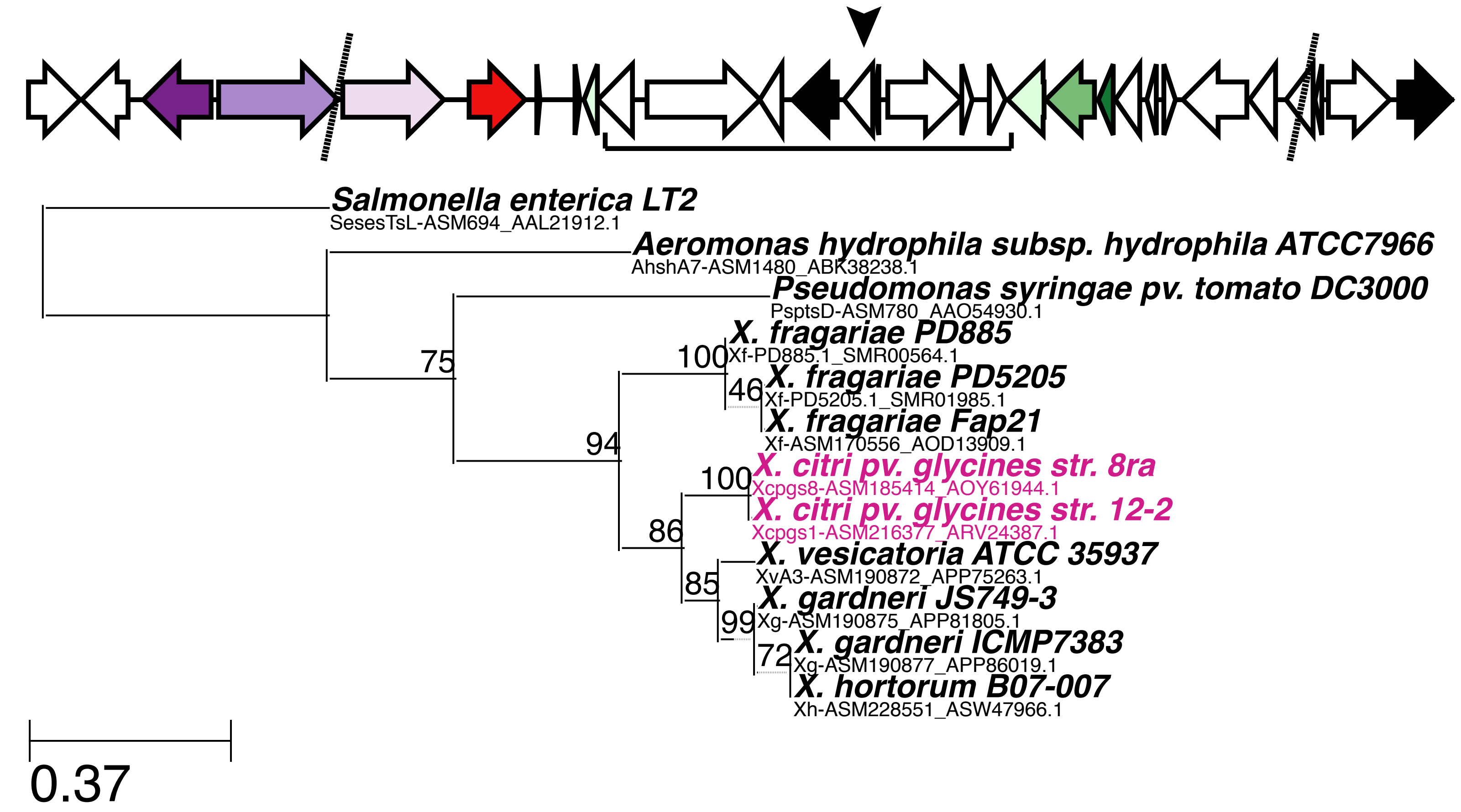

I) OG0015184 (function unknown)

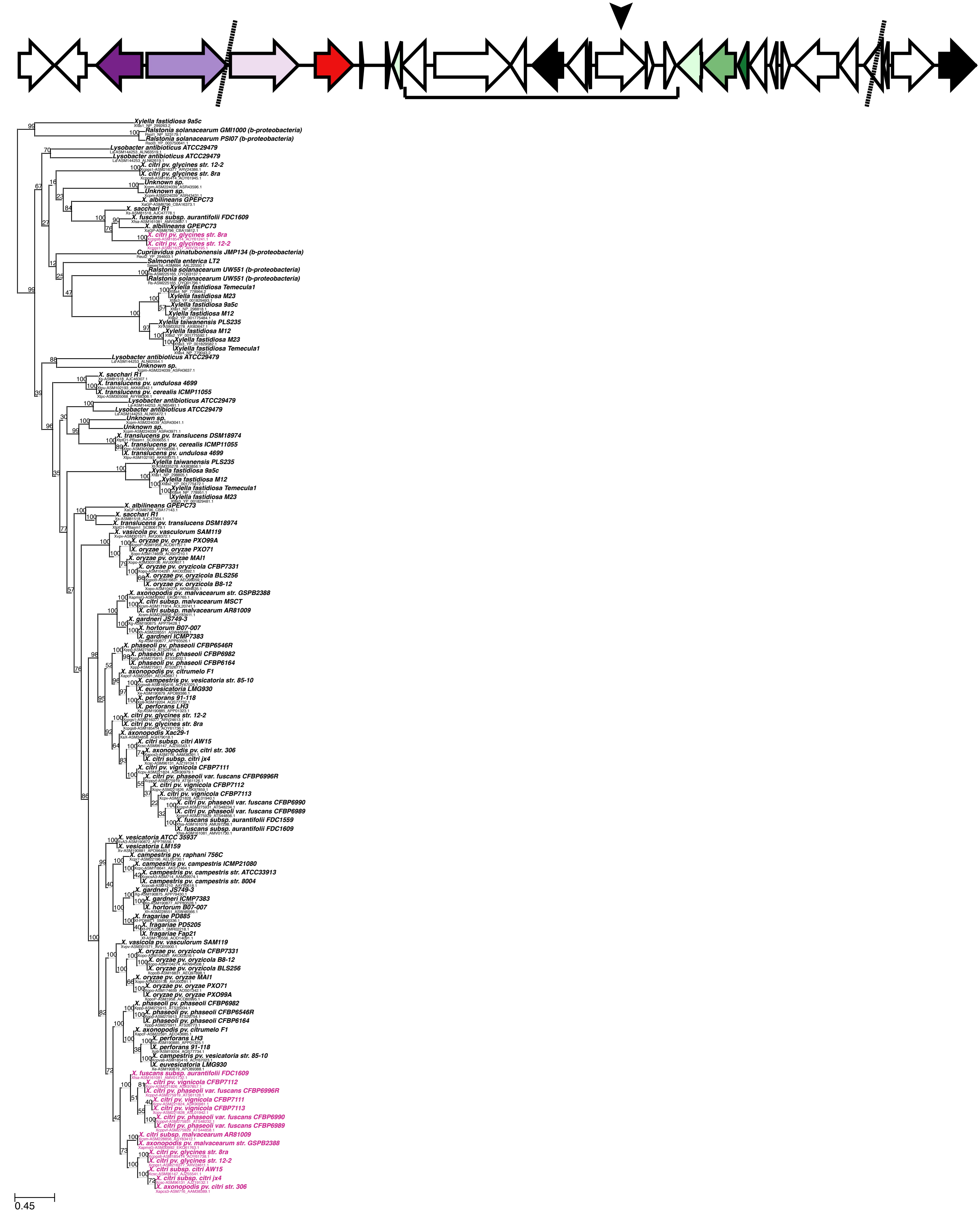

m) OG0004674 (function unknown)

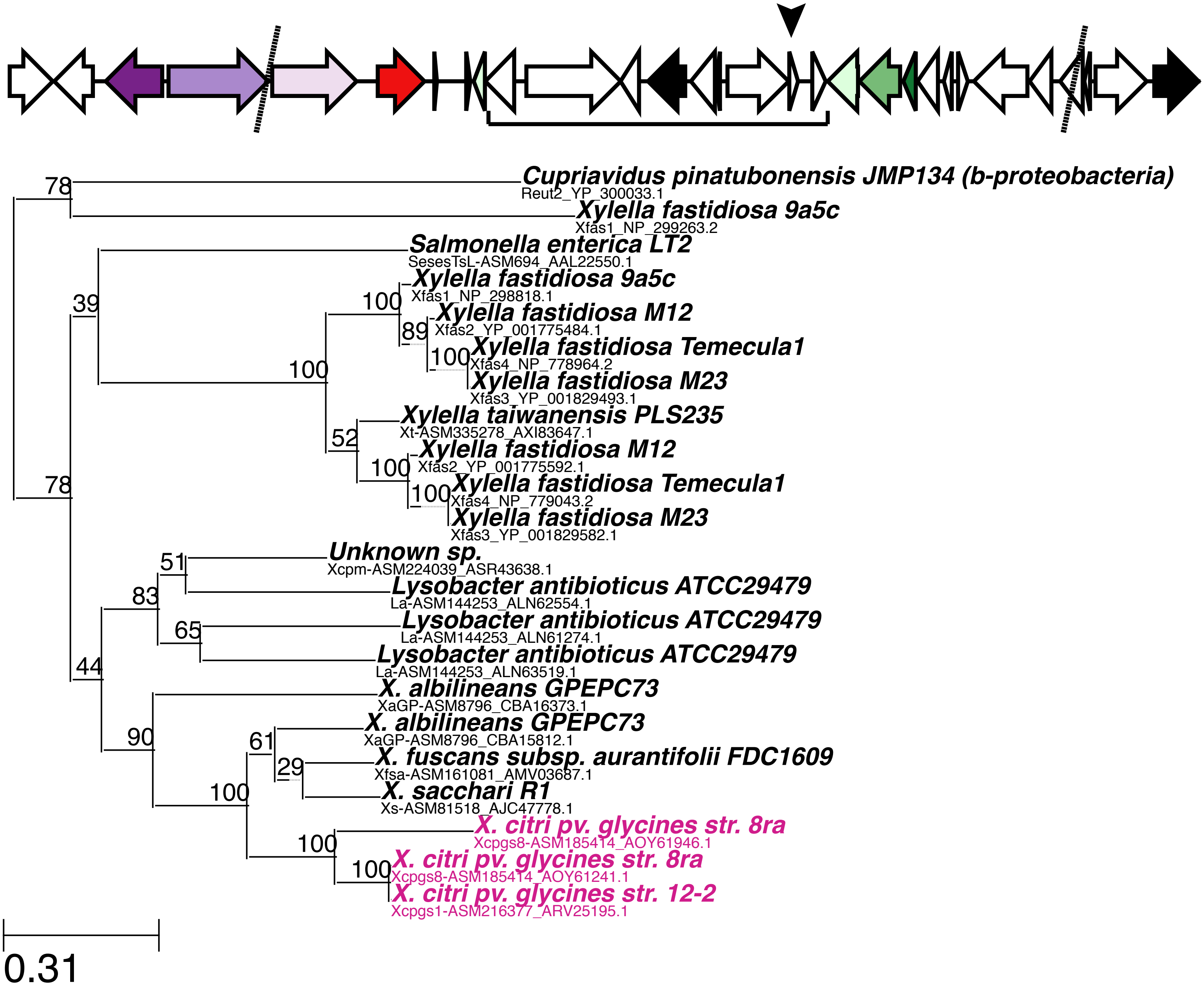

n) OG0005551 (CDP-diacylglycerol pyrophosphatase)

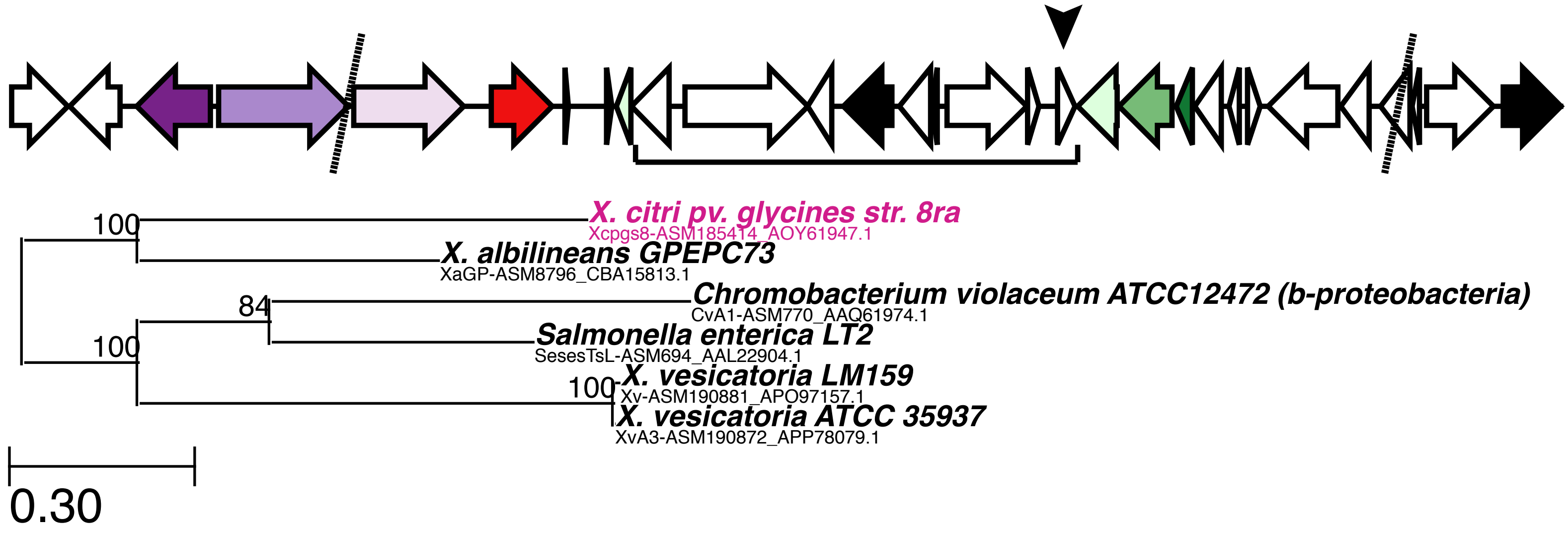

o) OG0003060 (peptidase m28)

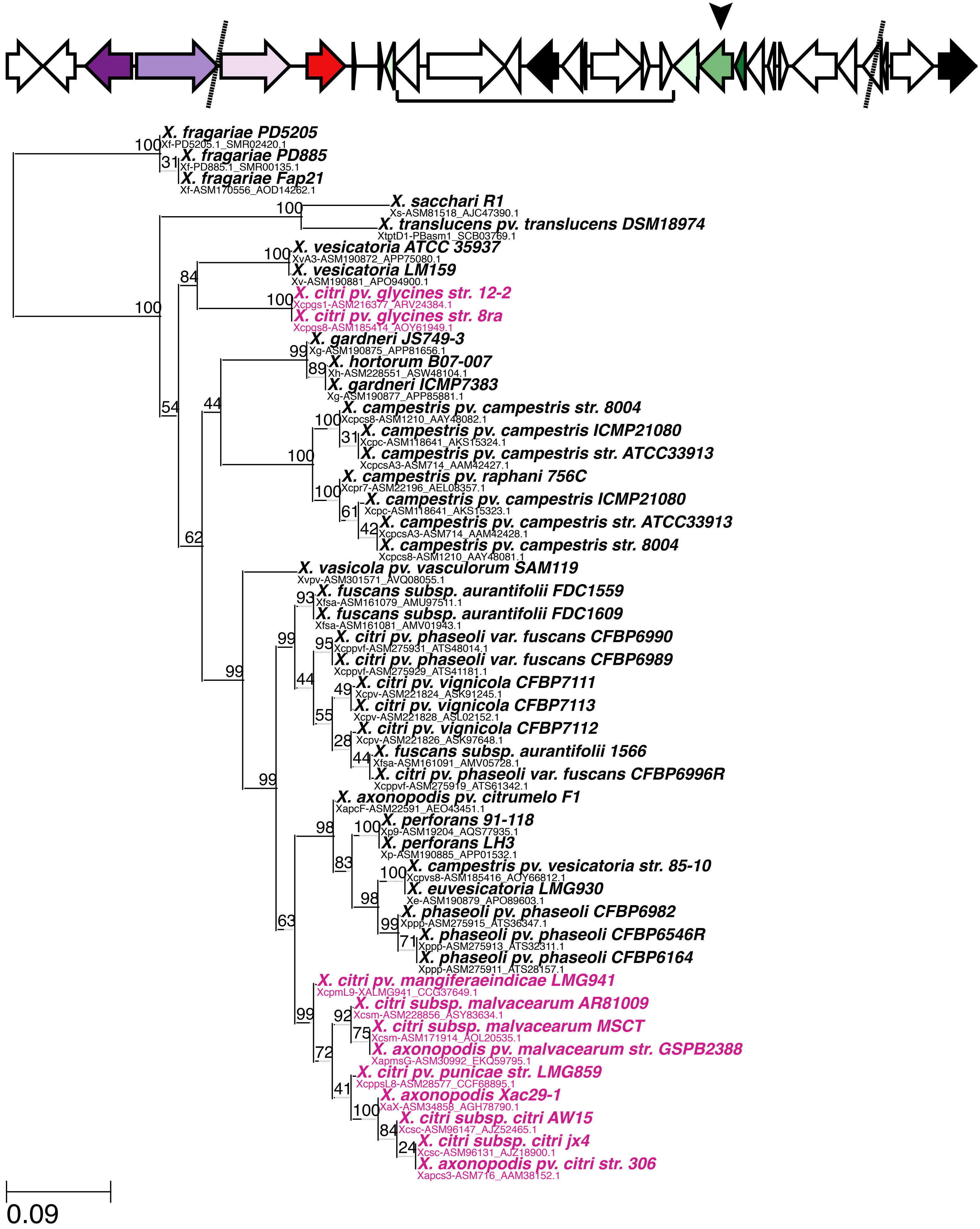

p) OG0001360 (large-conductance mechanosensitive ion channel)

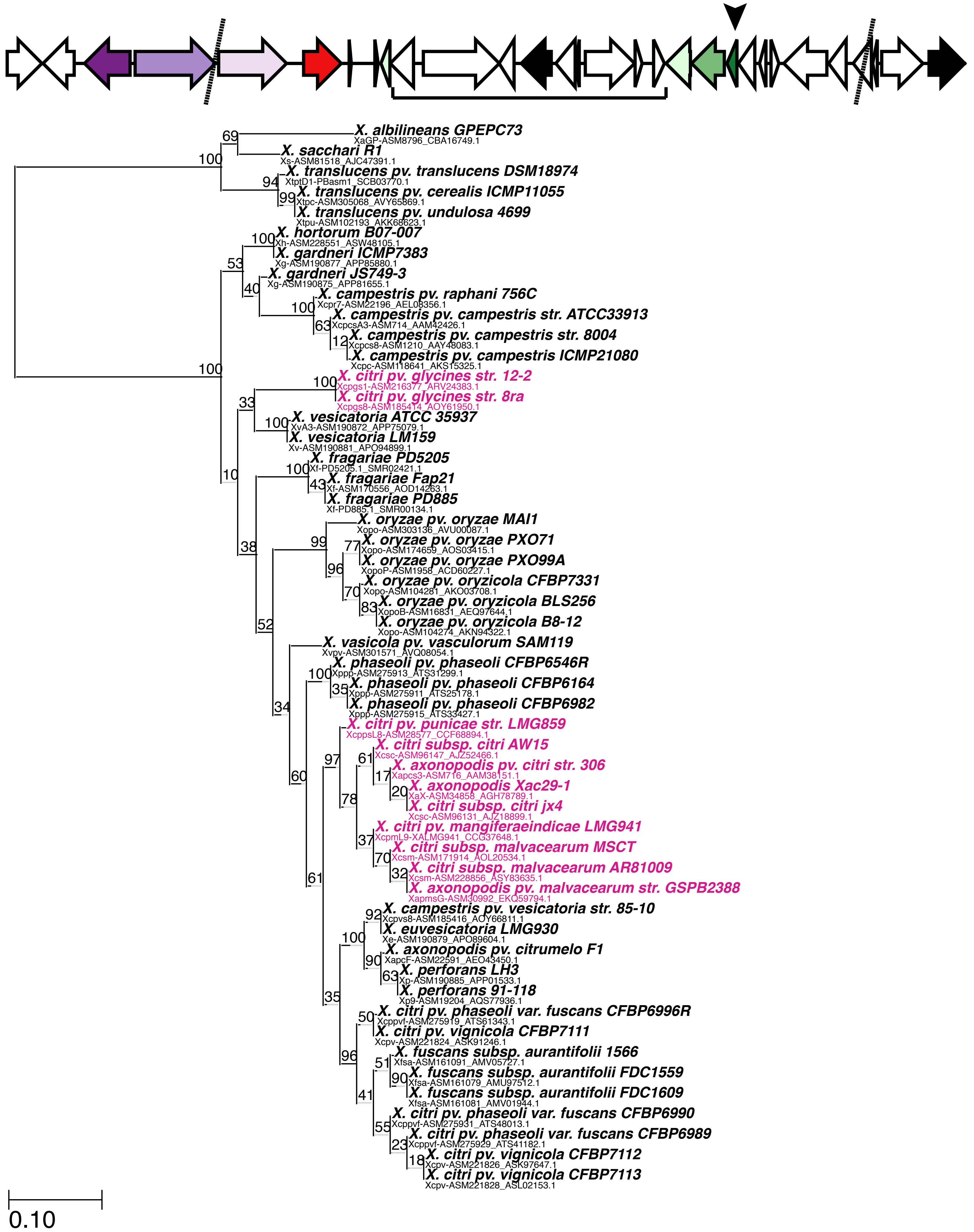

q) OG0000926 (fumarylacetoacetate hydrolase)

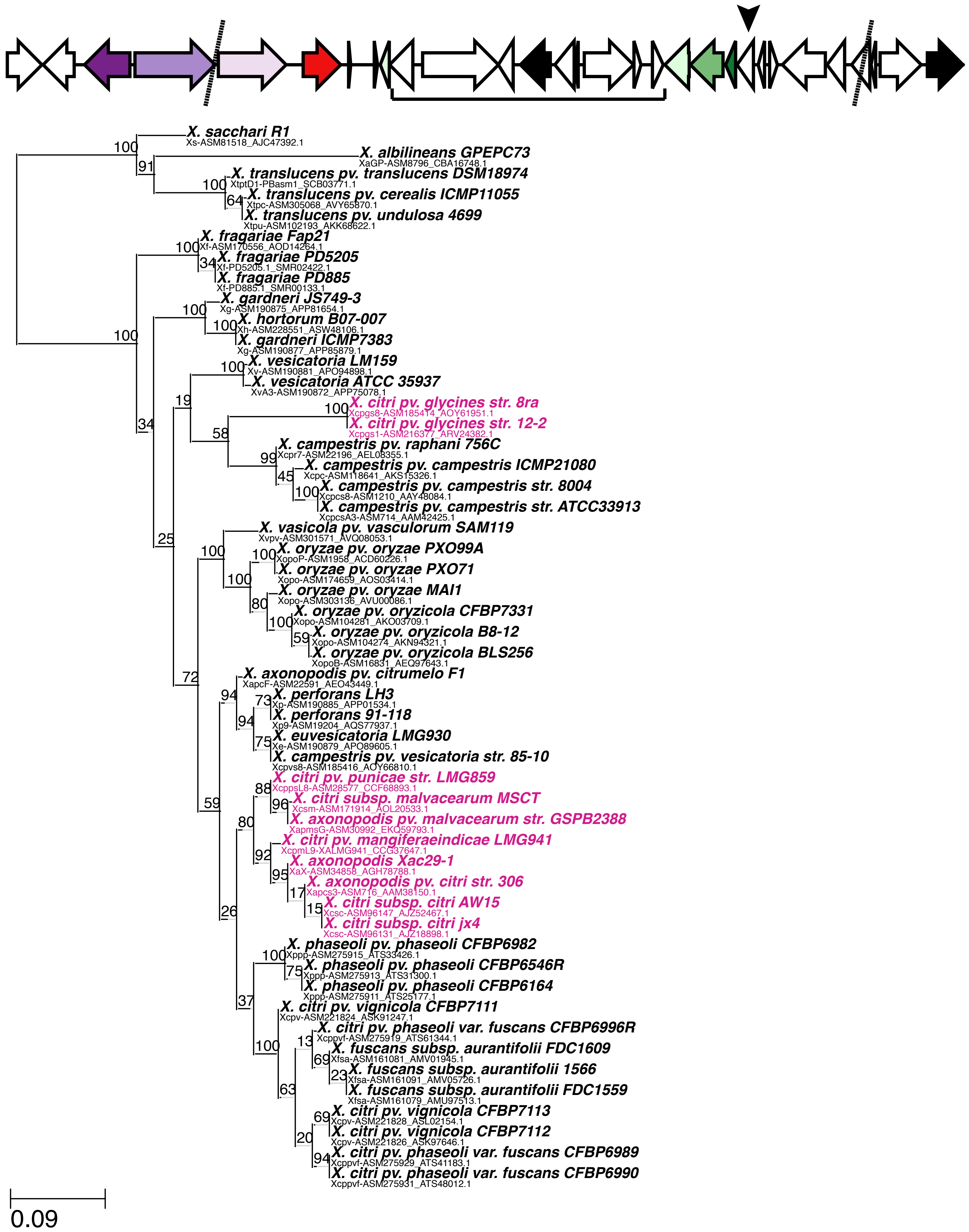

r) OG0002126 (function unknown)

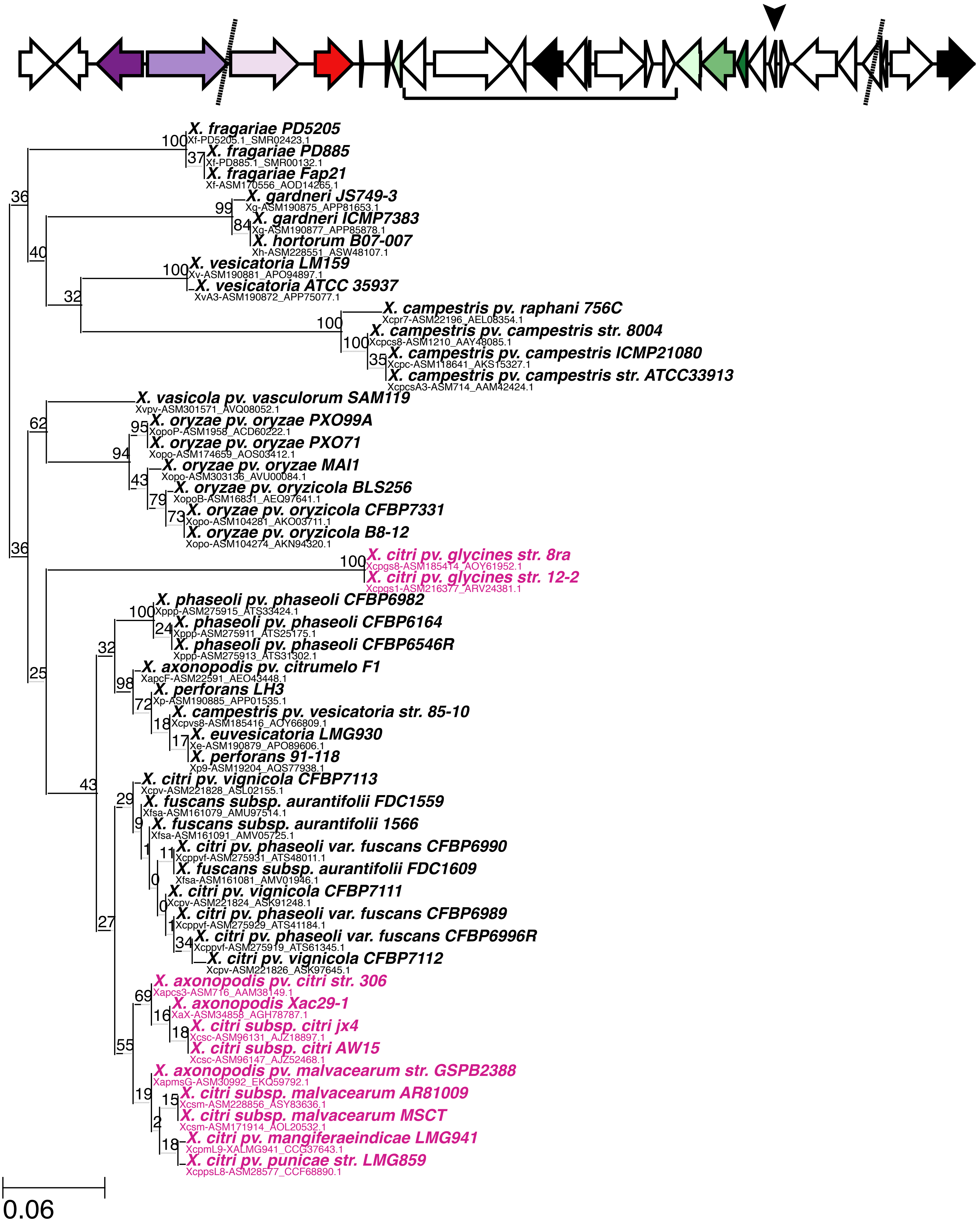

s) OG0001639 (Rieske [2Fe-2S] domain-containing protein)

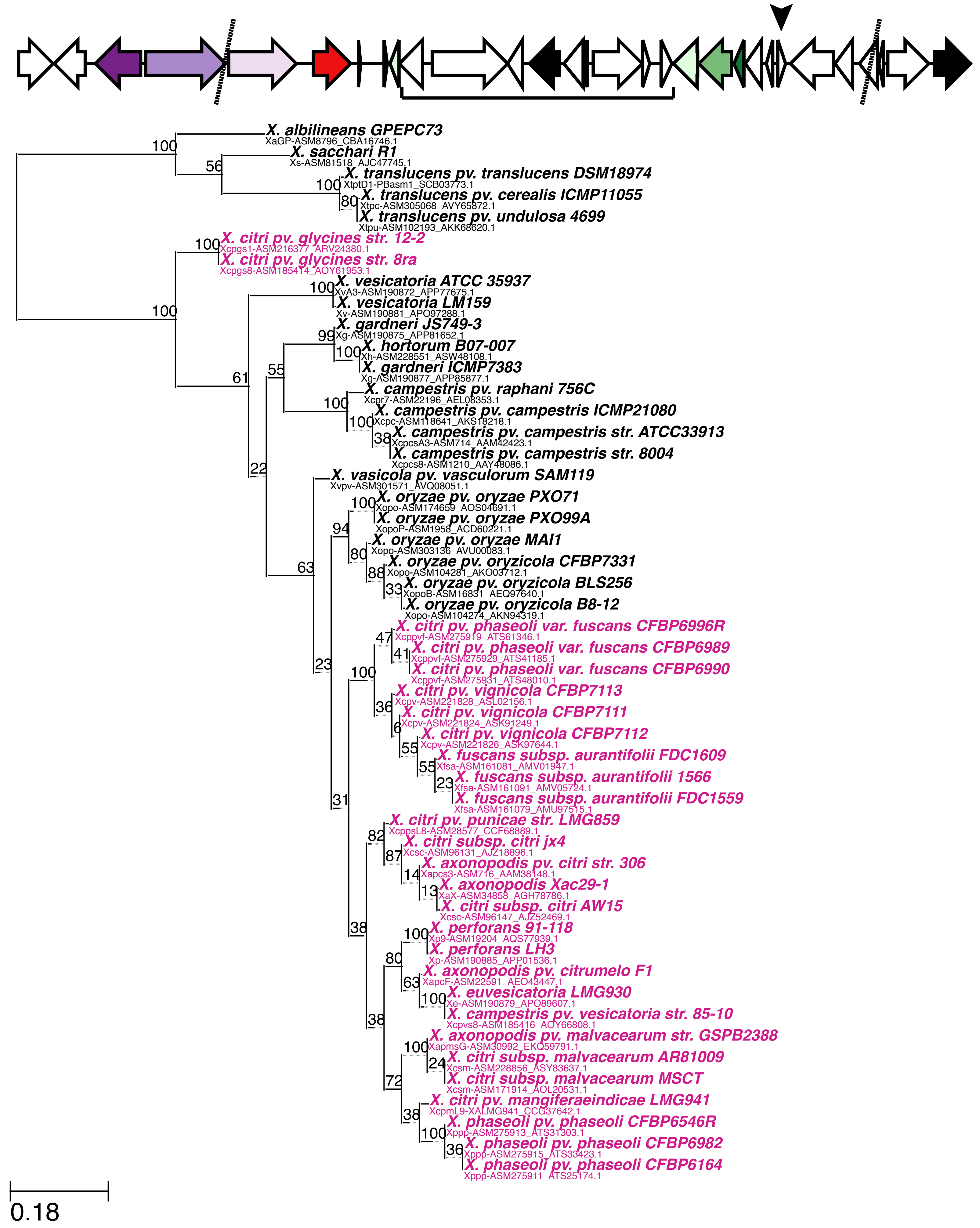

t) OG0001483 (citrate transporter)

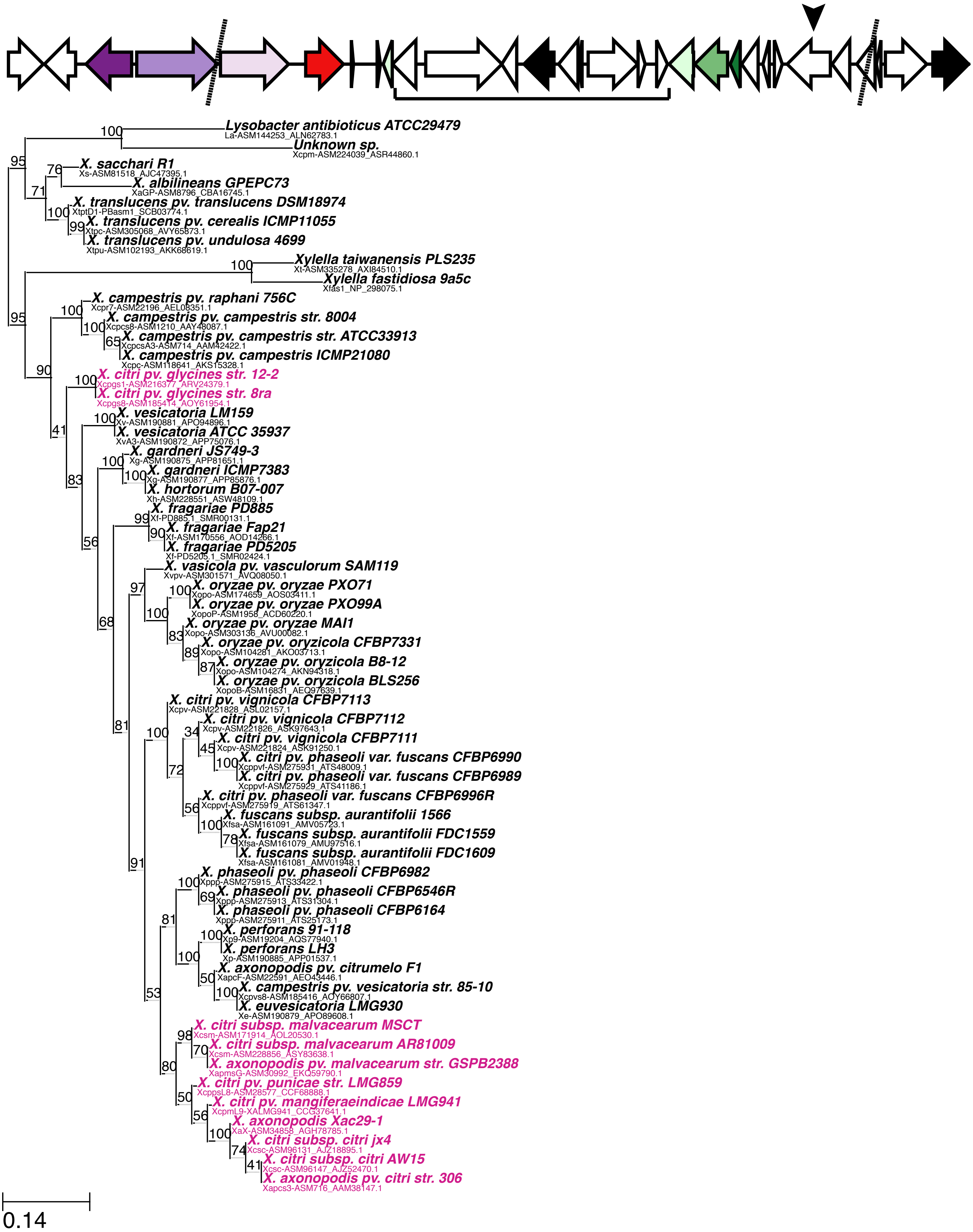

u) OG0001080 (putative methyltransferase)

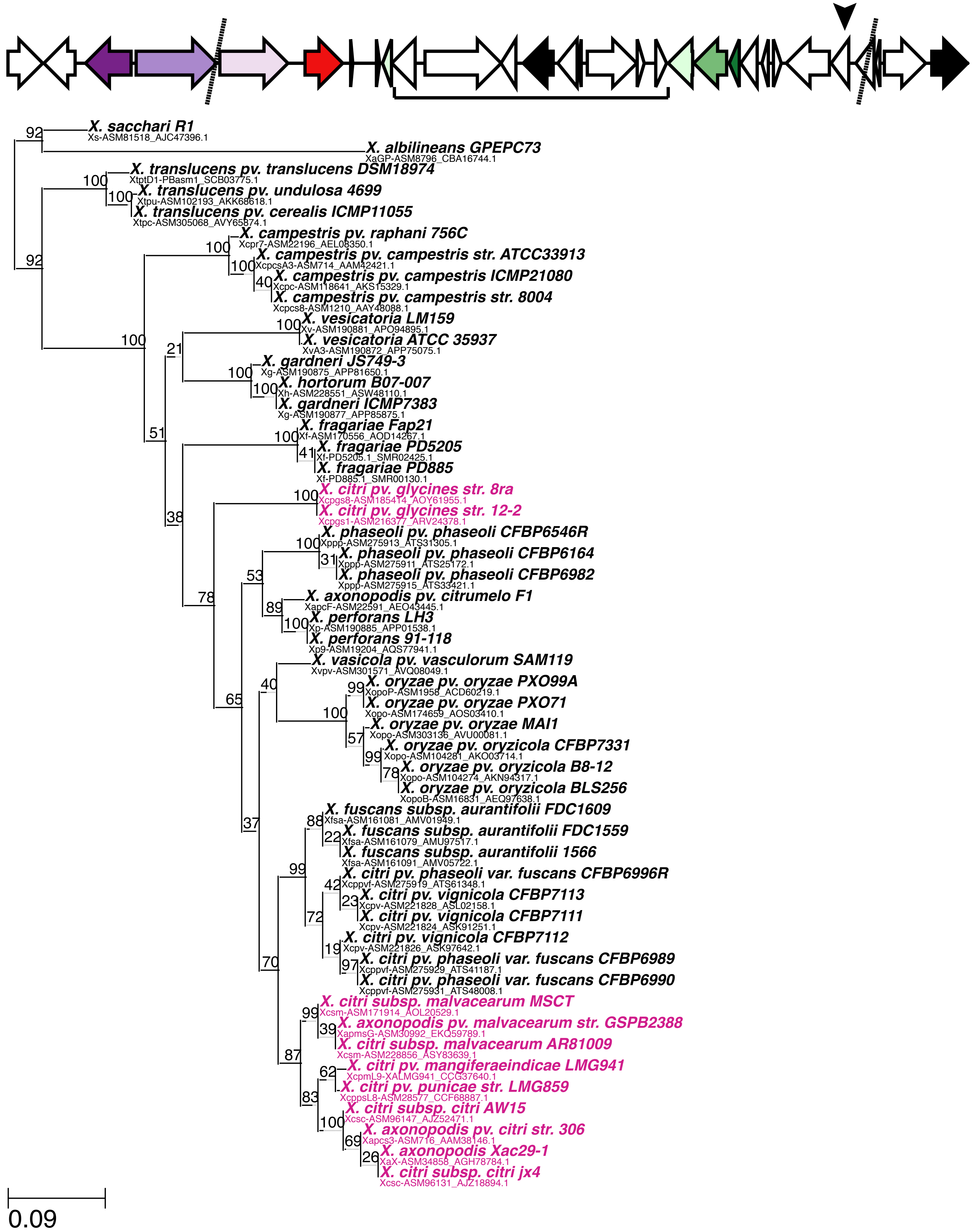

v) OG0000801, partition 2 (thiazole biosynthesis protein thiG)

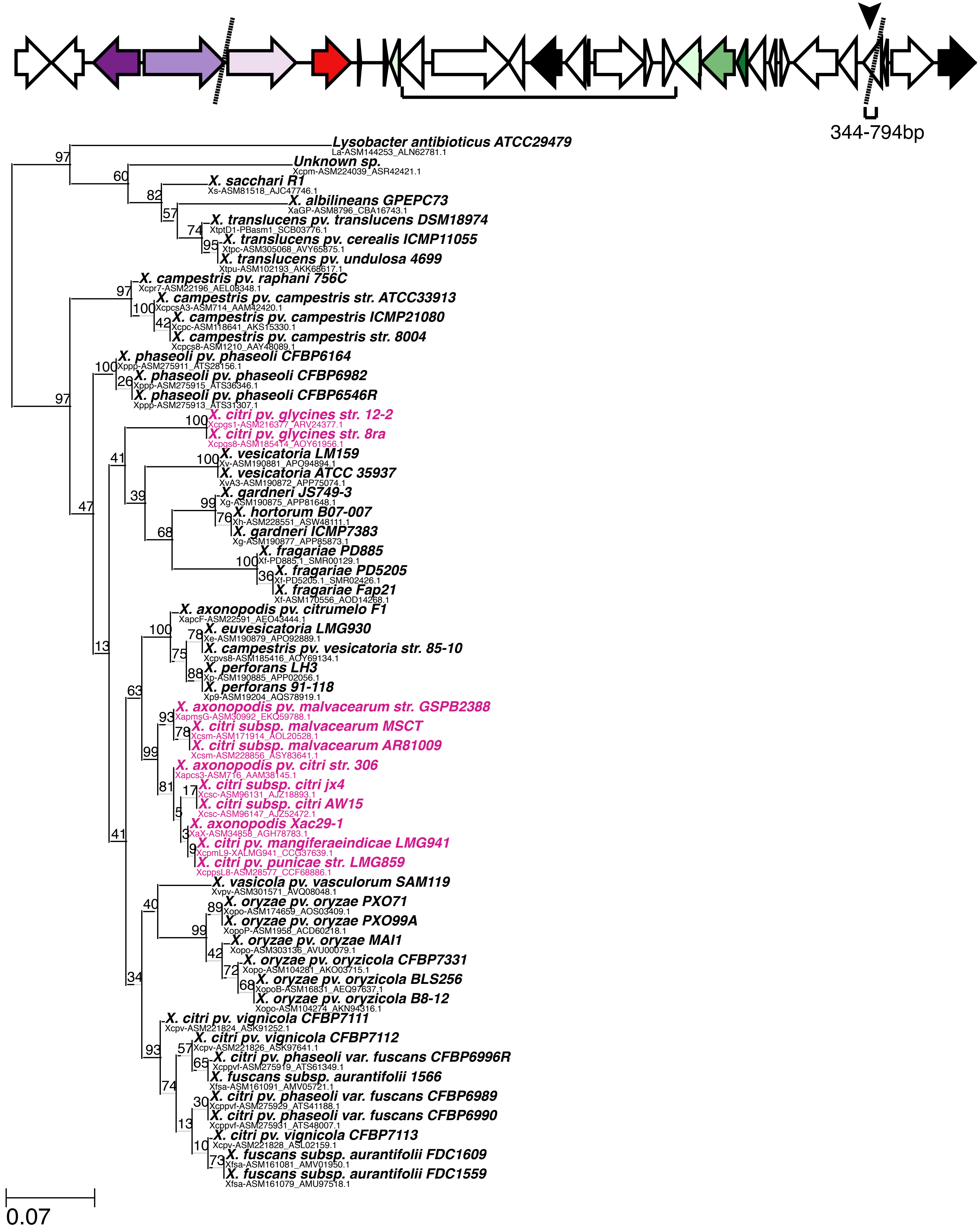

w) OG0000801, partition 1 (thiazole biosynthesis protein thiG)

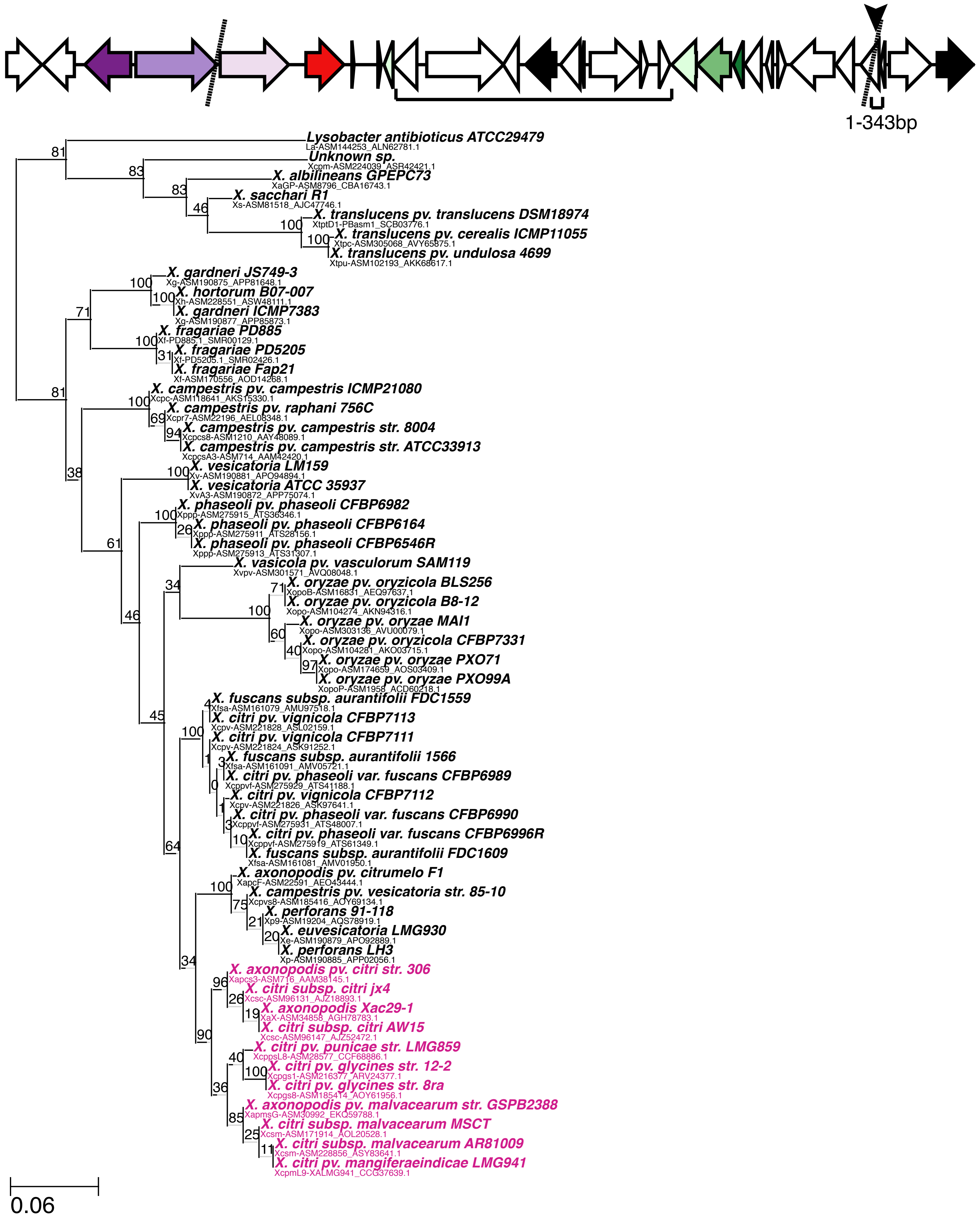

x) OG0001155 (thiazole biosynthesis protein thiS)

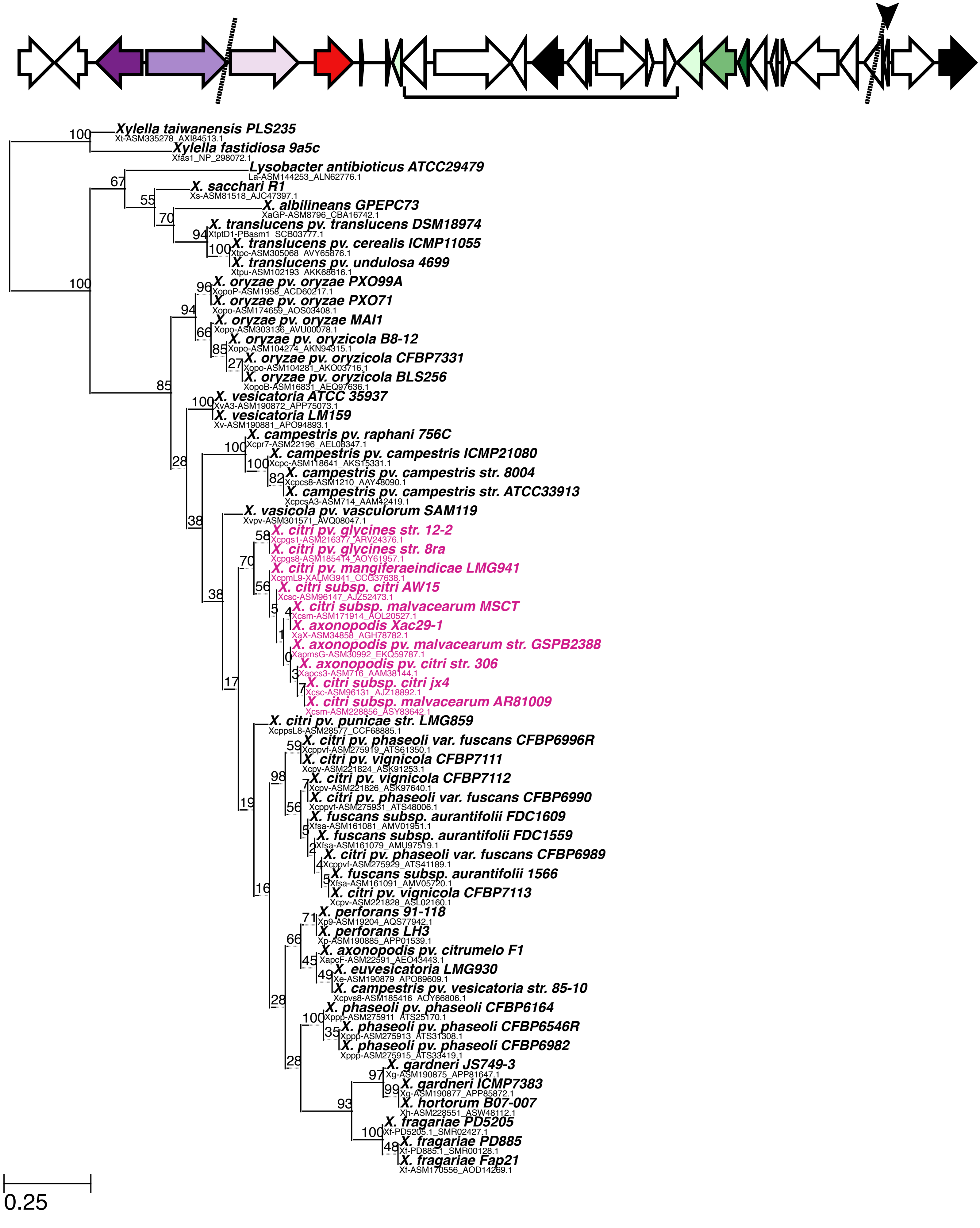

y) OG0001453 (esterase)

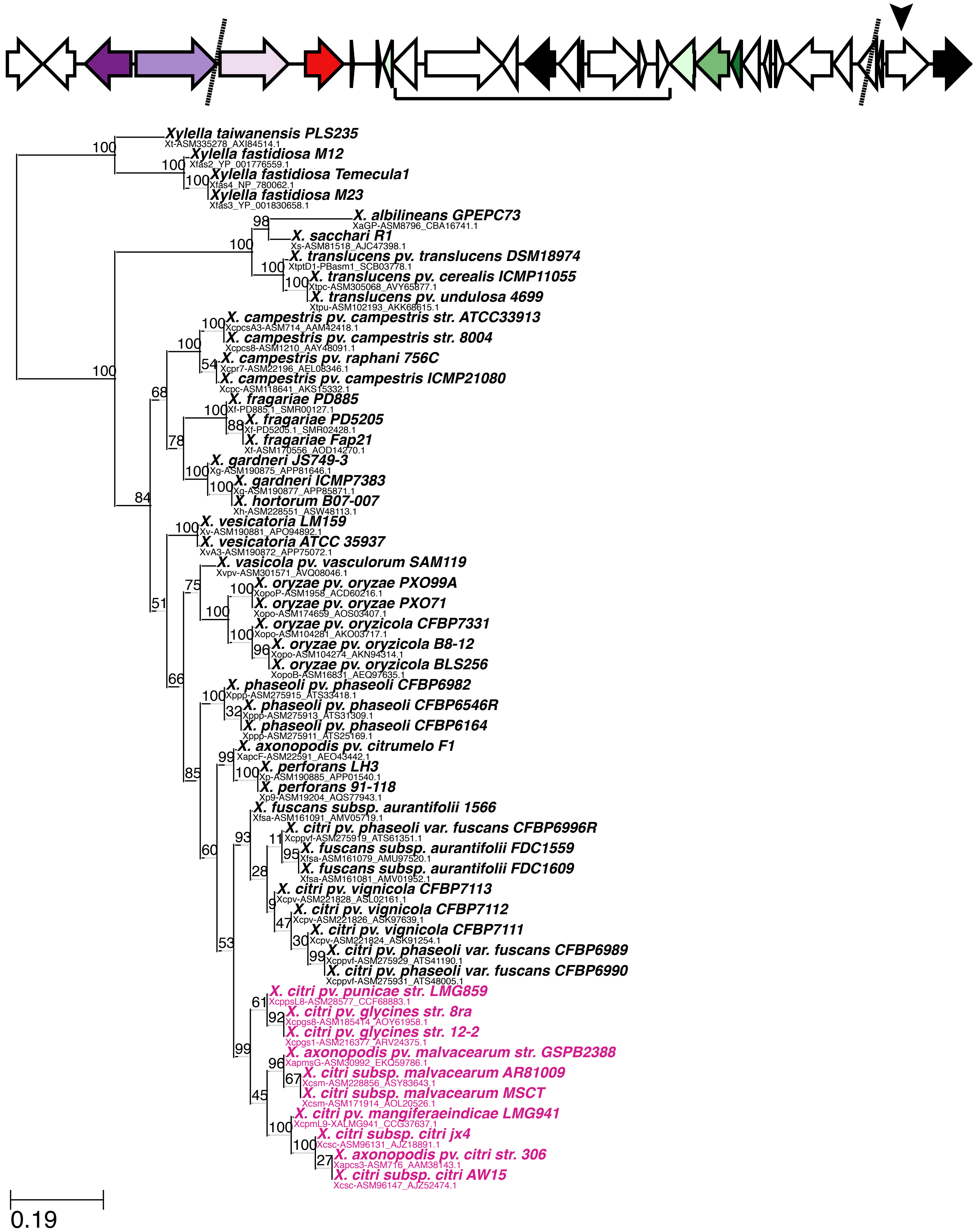

Supplement: Adobe PDF - abc4516_Figure_S7.pdf [file abc4516_Figure_S7.pdf]
