# Supplementary material for: Repeated gain and loss of a single gene modulates the evolution of vascular plant pathogen lifestyles
Source: Sci Adv. 2020 Nov 13;6(46):eabc4516. doi: 10.1126/sciadv.abc4516 (PMC7673761; doi:10.1126/sciadv.abc4516)

a) OG0001552 (magnesium and cobalt transport protein)

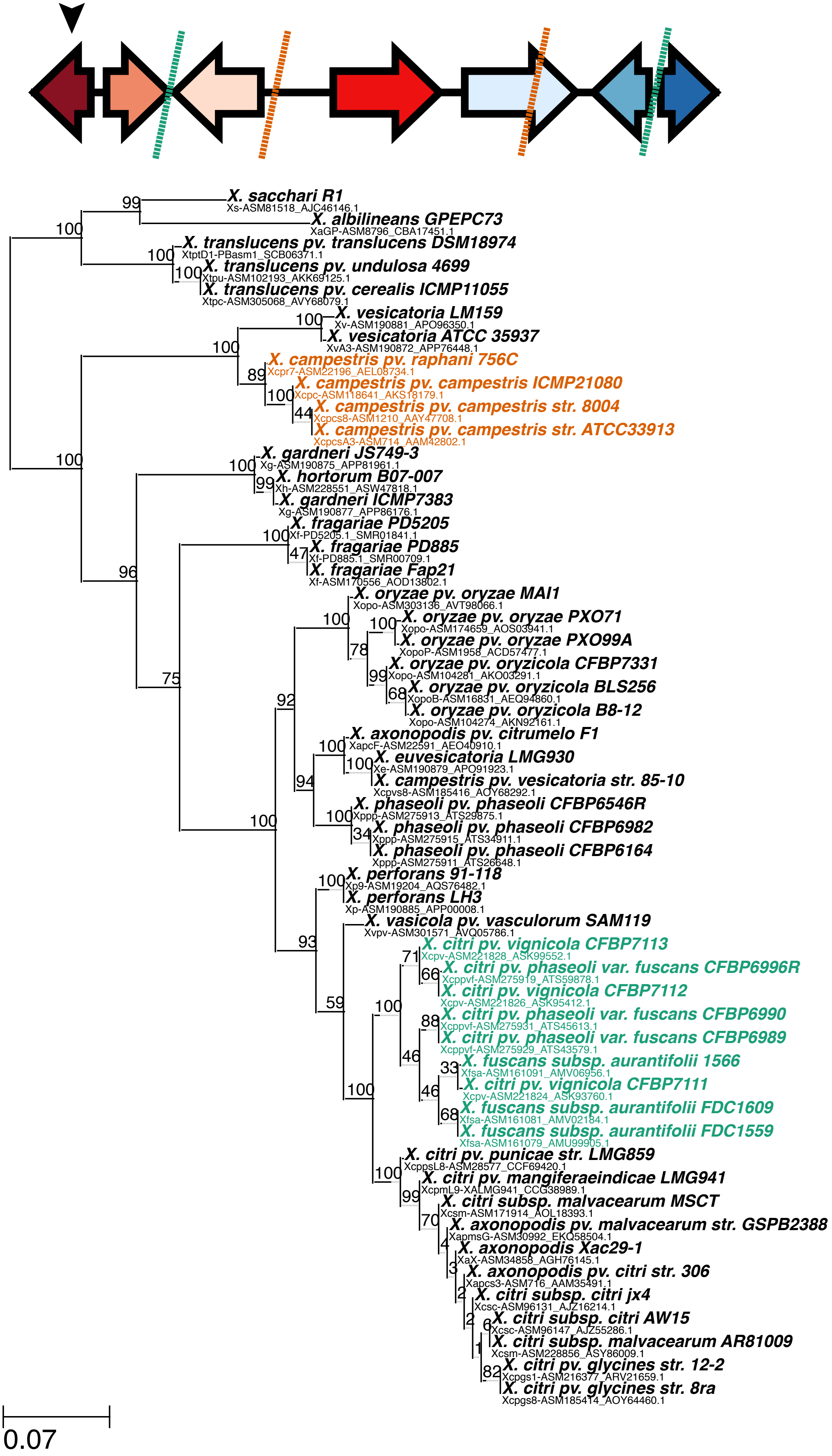

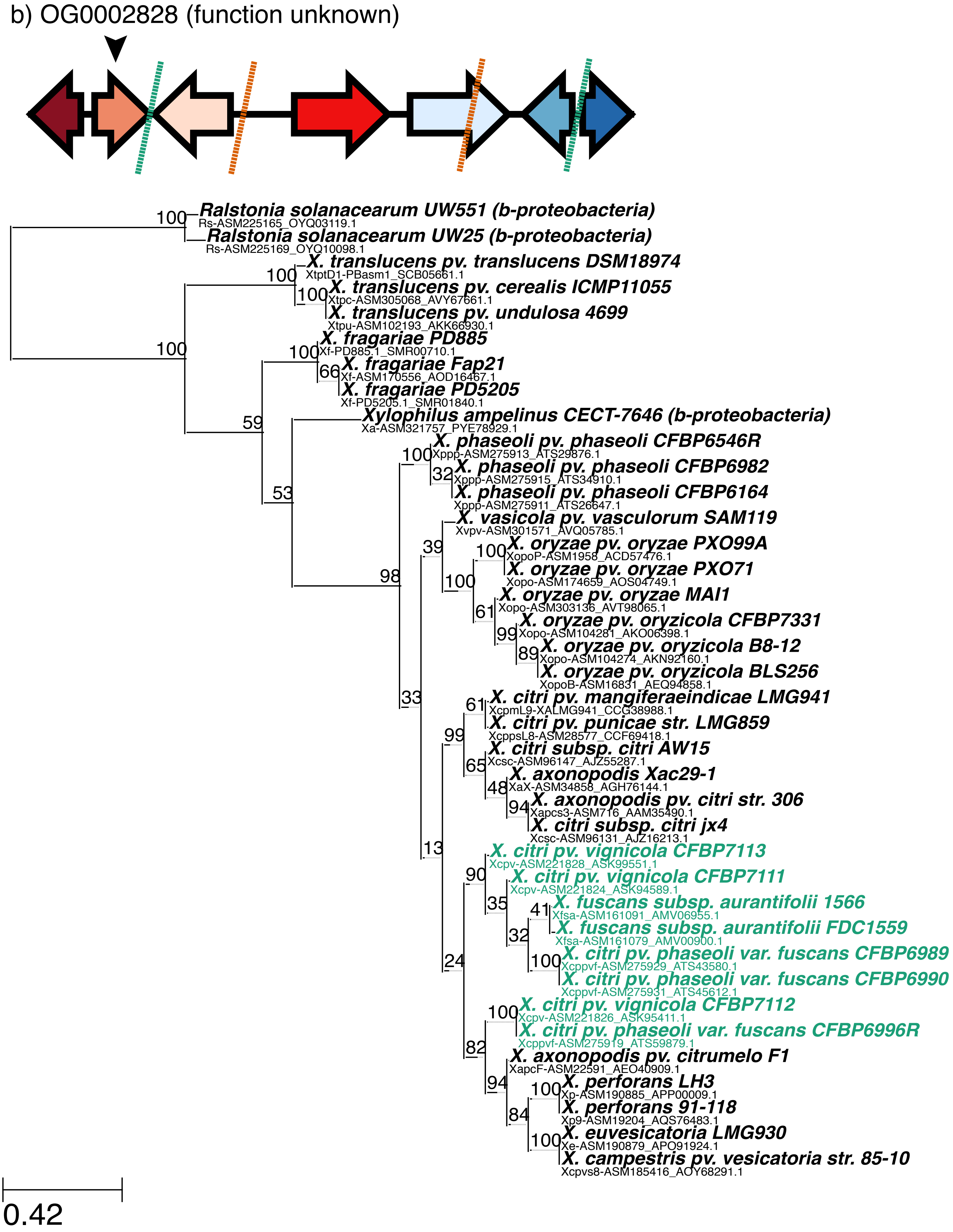

b) OG0002828 (function unknown)
Ralstonia solanacearum UW551 (b-proteobacteria)
Rs-ASM225165_OYQ03119.1
Ralstonia solanacearum UW25 (b-proteobacteria)
Rs-ASM225169_OYQ10098.1
X. translucens pv. translucens DSM18974
XtptD1-PBasm1_SCB05661.1
X. translucens pv. cerealis ICMP11055
Xtpc-ASM305068_AVY67661.1
X. translucens pv. undulosa 4699
Xtpu-ASM102193_AKK66930.1
X. fragariae PD885
Xf-PD885.1_SMR00710.1
X. fragariae Fap21
Xf-ASM170556_AOD16467.1
X. fragariae PD5205
Xf-PD5205.1_SMR01840.1
Xylophilus ampelinus CECT-7646 (b-proteobacteria)
Xa-ASM321757_PYE78929.1
X. phaseoli pv. phaseoli CFBP6546R
Xppp-ASM275913_ATS29876.1
X. phaseoli pv. phaseoli CFBP6982
Xppp-ASM275915_ATS34910.1
X. phaseoli pv. phaseoli CFBP6164
Xppp-ASM275911_ATS26647.1
X. vasicola pv. vasculorum SAM119
Xvpv-ASM301571_AVQ05785.1
X. oryzae pv. oryzae PXO99A
XopoP-ASM1958_ACD57476.1
X. oryzae pv. oryzae PXO71
Xopo-ASM174659_AOS04749.1
X. oryzae pv. oryzae MAI1
Xopo-ASM303136_AVT98065.1
X. oryzae pv. oryzicola CFBP7331
Xopo-ASM104281_AKO06398.1
X. oryzae pv. oryzicola B8-12
Xopo-ASM104274_AKN92160.1
X. oryzae pv. oryzicola BLS256
XopoB-ASM16831_AEQ94858.1
X. citri pv. mangiferaeindicae LMG941
XcpmL9-XALMG941_CCG38988.1
X. citri pv. punicae str. LMG859
XcppsL8-ASM28577_CCF69418.1
X. citri subsp. citri AW15
Xcsc-ASM96147_AJZ55287.1
X. axonopodis Xac29-1
XaX-ASM34858_AGH76144.1
X. axonopodis pv. citri str. 306
Xapcs3-ASM716_AAM35490.1
X. citri subsp. citri jx4
Xcsc-ASM96131_AJZ16213.1
X. citri pv. vignicola CFBP7113
Xcpv-ASM221828_ASK99551.1
X. citri pv. vignicola CFBP7111
Xcpv-ASM221824_ASK94589.1
X. fuscans subsp. aurantifolii 1566
Xfsa-ASM161091_AMV06955.1
X. fuscans subsp. aurantifolii FDC1559
Xfsa-ASM161079_AMV00900.1
X. citri pv. phaseoli var. fuscans CFBP6989
Xcppvf-ASM275929_ATS43580.1
X. citri pv. phaseoli var. fuscans CFBP6990
Xcppvf-ASM275931_ATS45612.1
X. citri pv. vignicola CFBP7112
Xcpv-ASM221826_ASK95411.1
X. citri pv. phaseoli var. fuscans CFBP6996R
Xcppvf-ASM275919_ATS59879.1
X. axonopodis pv. citrumelo F1
XapcF-ASM22591_AEO40909.1
X. perforans LH3
Xp-ASM190885_APP00009.1
X. perforans 91-118
Xp9-ASM19204_AQS76483.1
X. euvesicatoria LMG930
Xe-ASM190879_APO91924.1
X. campestris pv. vesicatoria str. 85-10
Xcpvs8-ASM185416_AOY68291.1
0.42

c) OG0000035 and 5’ UTR, partition 2 (amino acid permease)

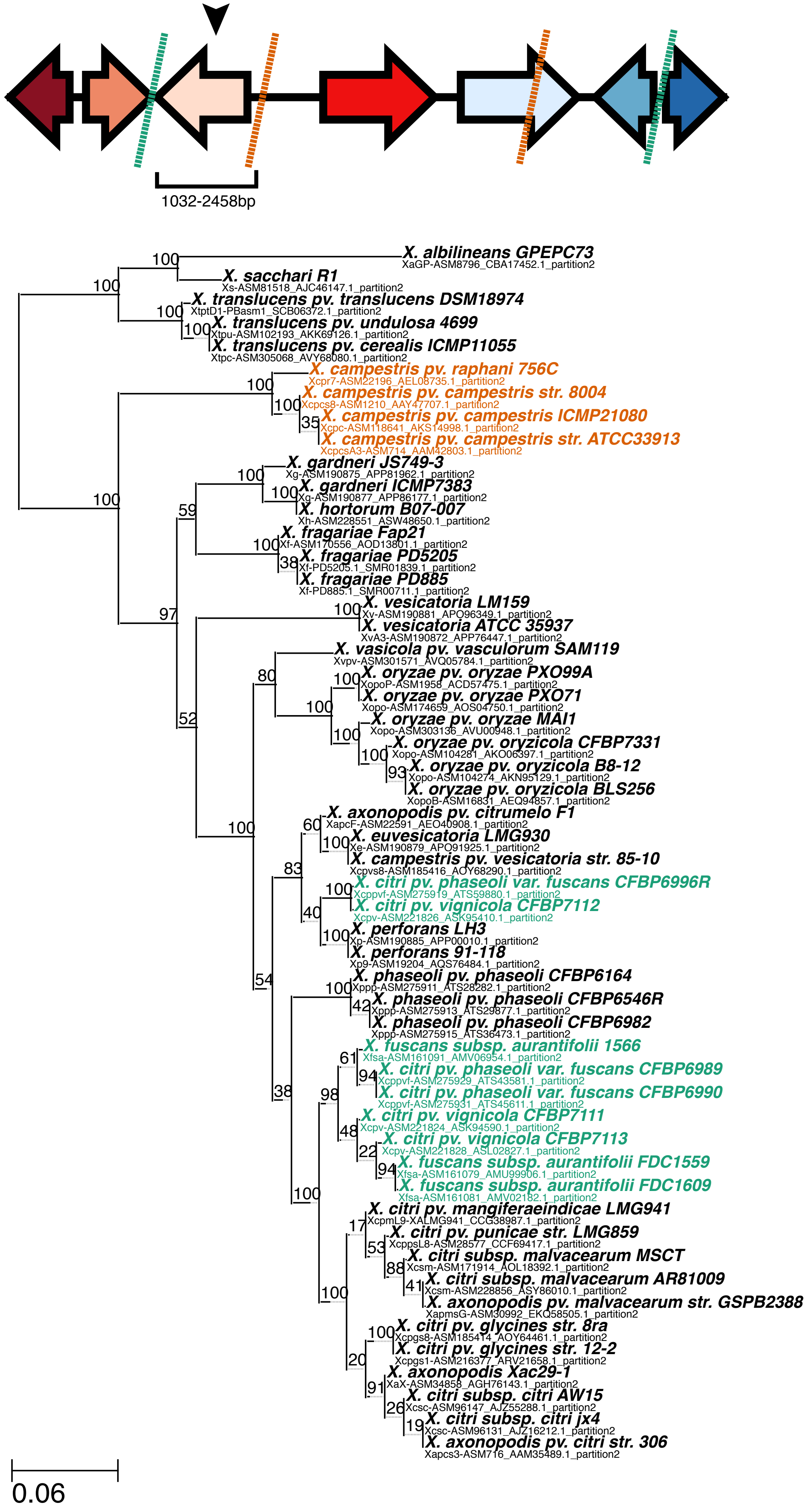

d) OG0000035 and 5' UTR, partition 1 (amino acid permease)

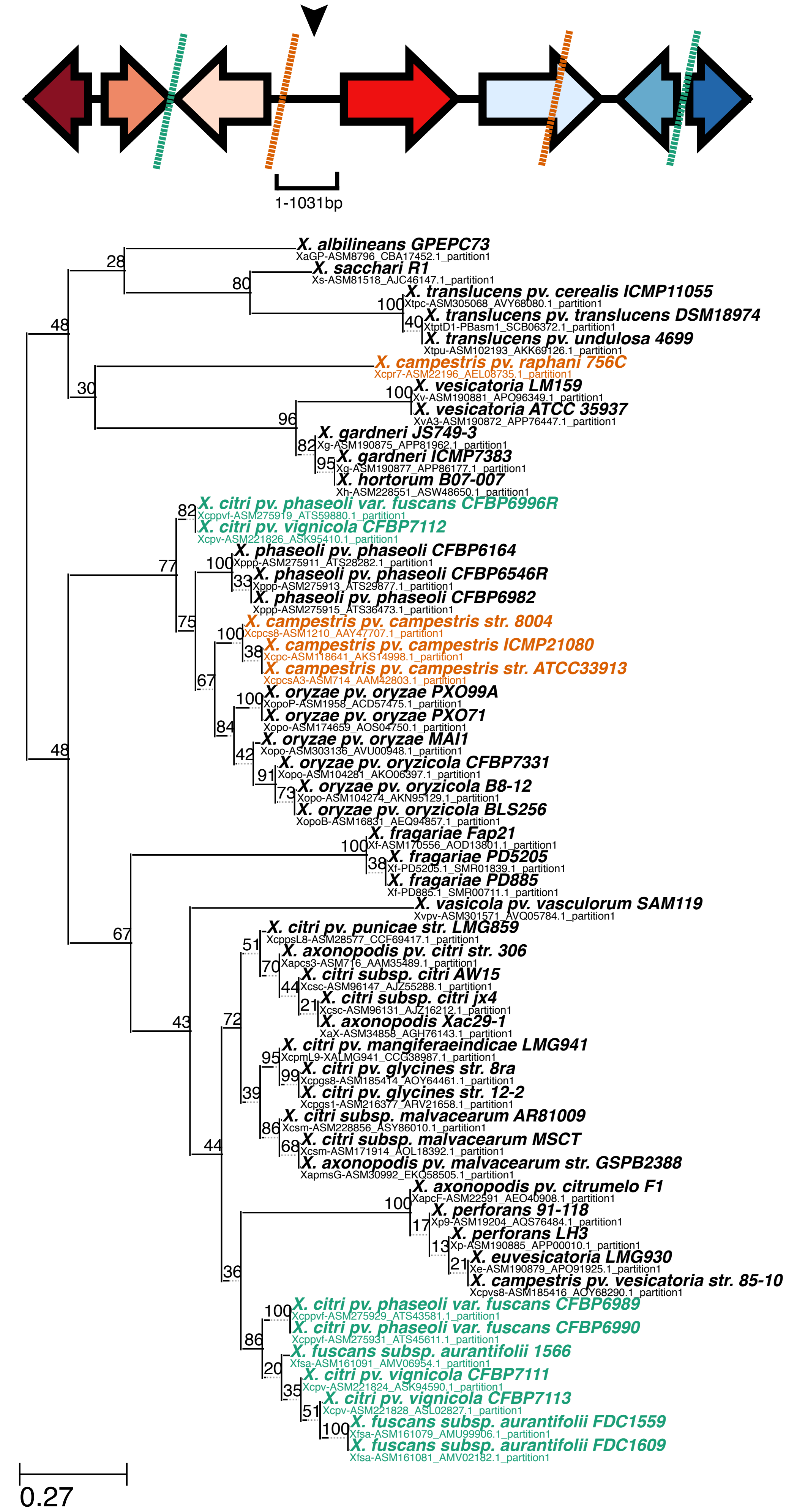

e) OG0000234, partition 1 (cellulase)

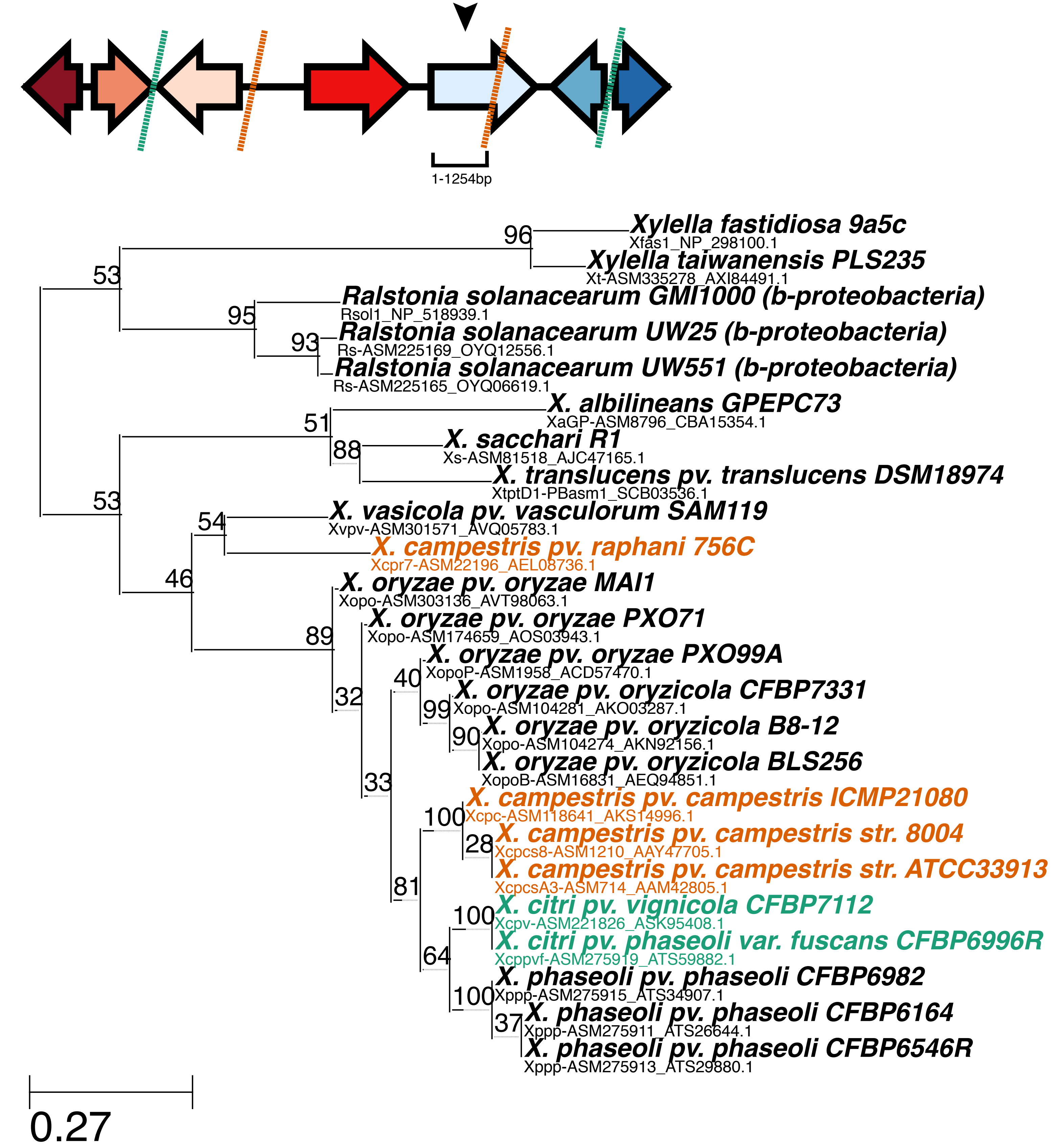

f) OG0000234, partition 2 (cellulase)

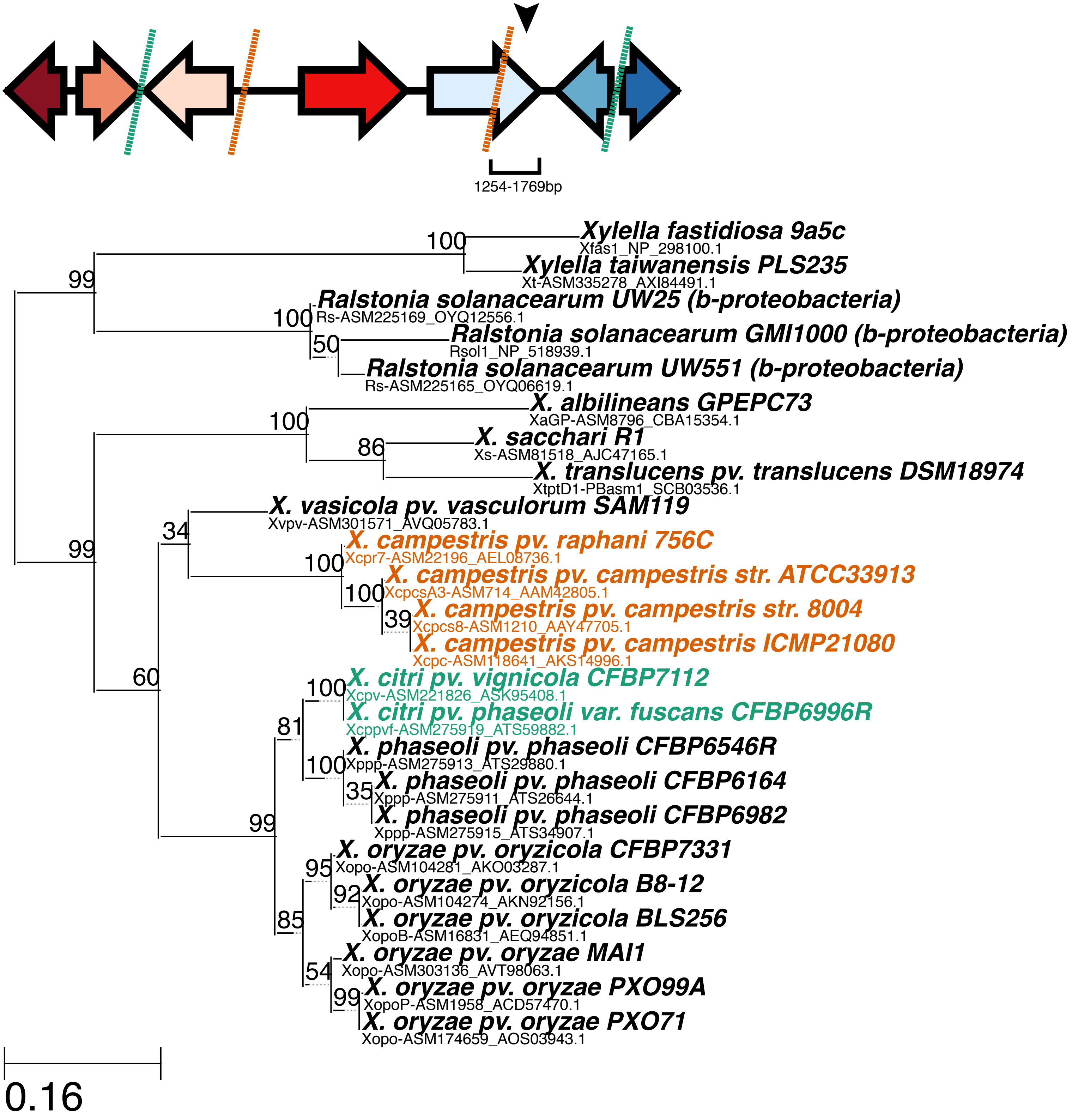

g) OG0002434 (integral membrane protein)

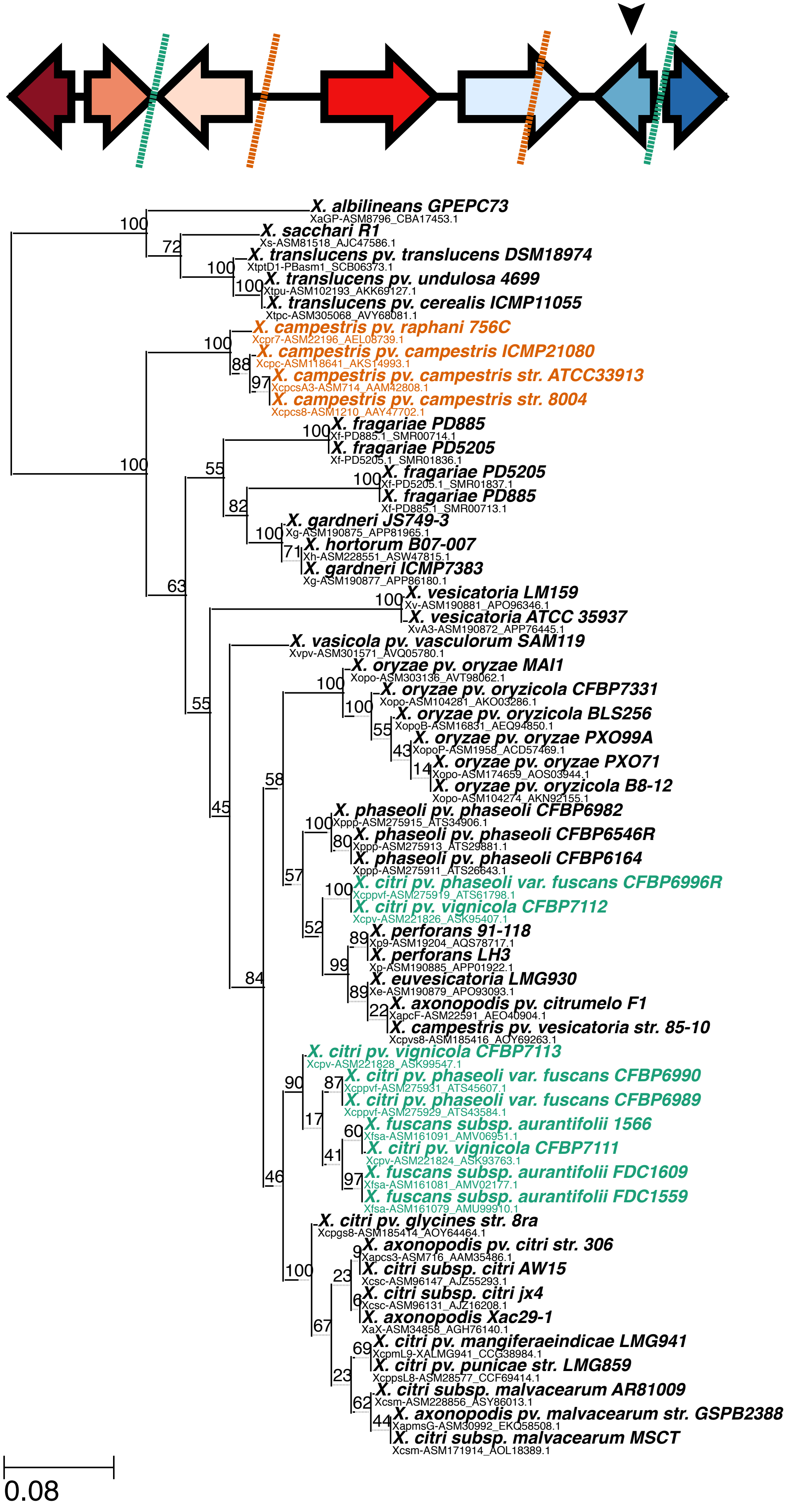

h) OG0002170 (methyltransferase)

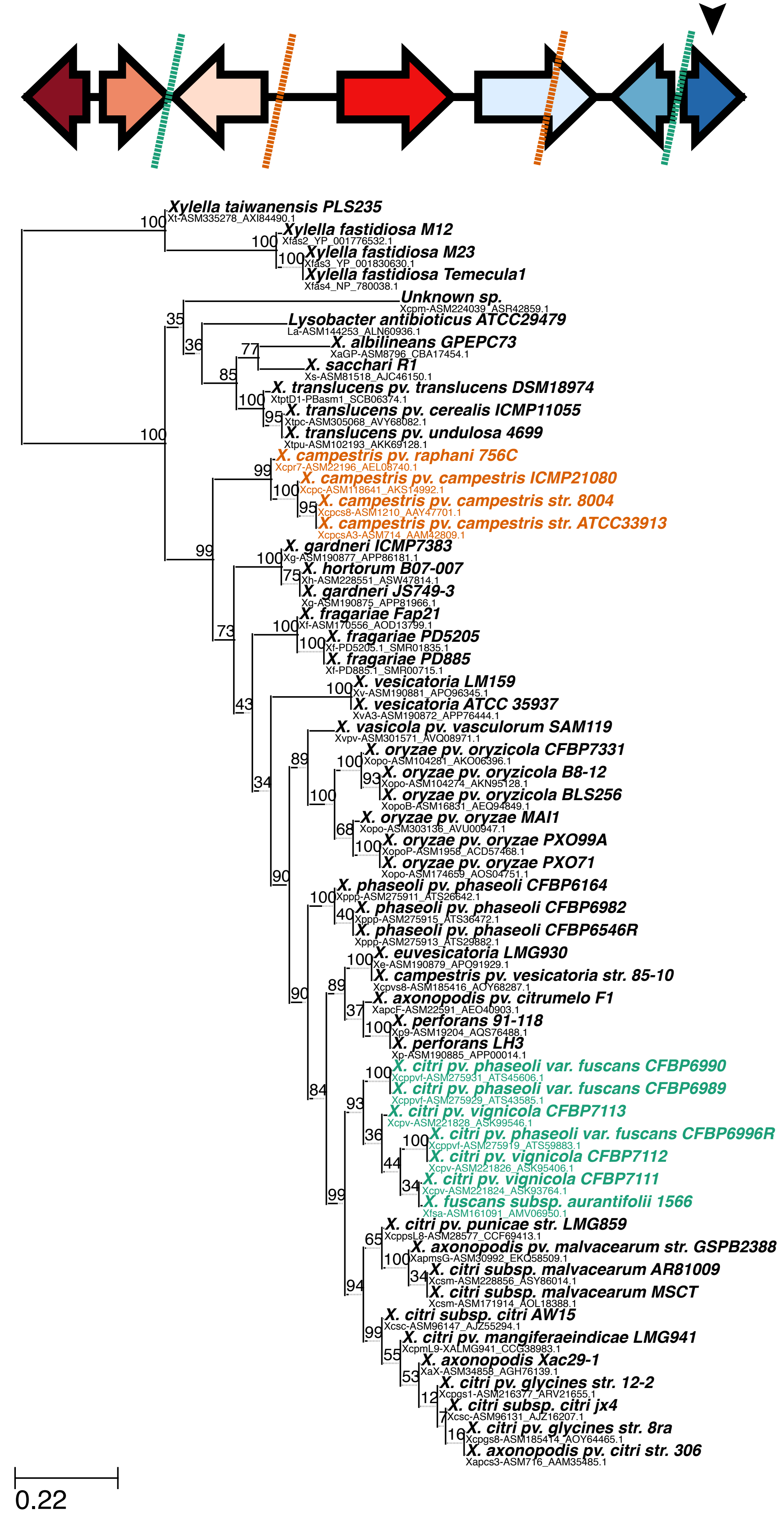

Supplement: Adobe PDF - abc4516_Figure_S8.pdf [file abc4516_Figure_S8.pdf]
